# Brønsted acid-catalyzed aromatic annulation of alkoxyallenes with naphthols: a reaction sequence to larger π-conjugated naphthopyrans with aggregation-induced emission characters†
†Electronic supplementary information (ESI) available. CCDC 1861979 and 1861980. For ESI and crystallographic data in CIF or other electronic format see DOI: 10.1039/c8sc03837f


**DOI:** 10.1039/c8sc03837f

**Published:** 2018-11-02

**Authors:** Jinlong Zhang, Lu Zhu, Kang Shen, Huameng Yang, Xiao-Chun Hang, Gaoxi Jiang

**Affiliations:** a State Key Laboratory for Oxo Synthesis and Selective Oxidation , Center for Excellence in Molecular Synthesis , Suzhou Research Institute of LICP , Lanzhou Institute of Chemical Physics (LICP) , Chinese Academy of Sciences , Lanzhou 730000 , China . Email: gxjiang@licp.cas.cn; b Key Laboratory of Flexible Electronics (KLOFE) , Institute of Advanced Materials (IAM) , Nanjing Tech University (NanjingTech) , 30 South Puzhu Road , Nanjing 211800 , China . Email: iamxchhang@njtech.edu.cn

## Abstract

A practical and readily scalable reaction sequence was developed for the straightforward synthesis of a new family of larger π-conjugated naphthopyrans by a Brønsted acid-catalyzed aromatic annulation of alkoxyallenes with inert naphthols. The cascade pathway involves allylation/cyclization/debenzyloxylation/isomerization/dehydration. The new class of solid state diphenylmethylene substituted naphthopyrans are fluorescent emissive and proved to have aggregation-induced emission (AIE) behavior.

## Abstract 2

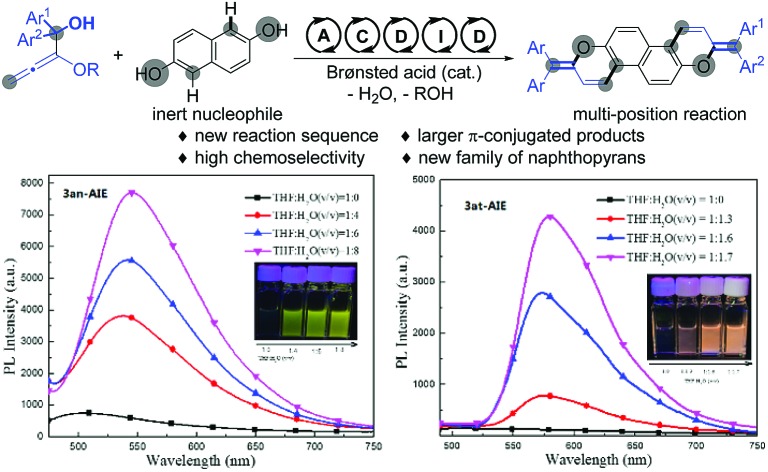
A practical and readily scalable reaction sequence was developed for the straightforward synthesis of a new family of larger π-conjugated naphthopyrans by a Brønsted acid-catalyzed aromatic annulation of alkoxyallenes with inert naphthols.

## 

Naphthopyran represents a unique unit in a wide family of natural products;^1^ it has been widely studied in photochromic chromenes (Scheme 1, **A**) and applied to commercially available photochromic lenses.^2^ For examples, the natural product **B** was isolated from the roots of *Pentas bussei*, a plant in Kenya that was used as a remedy for gonorrhea, syphilis, and dysentery by decoction of the roots. Conocurvone **C**, an extract from the endemic Australian shrub *Conospermum* sp. (Proteaceae), can inhibited the cytopathic effects of HIV-1 infection. Despite these important synthetic advances in strong acid-promoted cyclizations and transition metal-catalyzed annulations, the available methods are extensively limited to provide 3,3-disubstituted naphthopyrans (such as **A** and **B**).^3^ In principle, the extension of the conjugated π-system might endow materials with extraordinary properties in luminescence,^4^ organic electronics,^5^ and photovoltaic devices.^6^ Therefore, the development of efficient methods for the discovery of a new family of naphthopyrans with a larger conjugated π-system (Scheme 1, **D**) would be of great significance, yet remains challenging, probably owing to the unavoidable obstacles to compose these molecules.^7^ Recently, we realized a phosphoric acid-catalyzed exclusively linear allylation of alkoxyallenes with reactively enolated pyrazolones through a hydrogen-bonding interaction (Scheme 1, eqn (1)).^8^ Nevertheless, the transformation between alkoxyallenes and inert nucleophiles is still unknown reasonably on account of the limitation of their reactivity and reaction mode.^9^ Exploitation of straightforward and efficient access to achieve synthetic efficiency has always been a primary goal for the chemical sciences. Combining different kinds of reactions into cascades is one of the most effective strategies to enable expeditious synthesis of complex molecules.^10^ As is well documented, benzhydrol motifs are very versatile in Brønsted acid-catalyzed transformations by virtue of the high reactivity *via* hydroxyl release.^11^ We anticipate that implantation of such a reactive moiety into alkoxyallenes might lead to novel cascades by reaction with other nucleophiles bearing multiple reactive sites. The cascade reaction might provide practical and rapid access to larger π-conjugated aromatic compounds. Herein, we present an unprecedented allylation/cyclization/debenzyloxylation/isomerization/dehydration cascade reaction between benzhydrol alkoxyallenes and inert naphthols by a simple Brønsted acid catalysis under mild reaction conditions, affording a series of larger π-conjugated diphenylmethylene substituted naphthopyrans (Scheme 1, eqn (2)). The new class of naphthopyrans have aggregation-induced emission (AIE)^12^ and luminescence in the solid state ranging from the yellow to the near-infrared region. Most of those compounds are quite inert to UV light irradiation, which is promising for their potential utilization for long term and precise tracking.

**Scheme 1:**
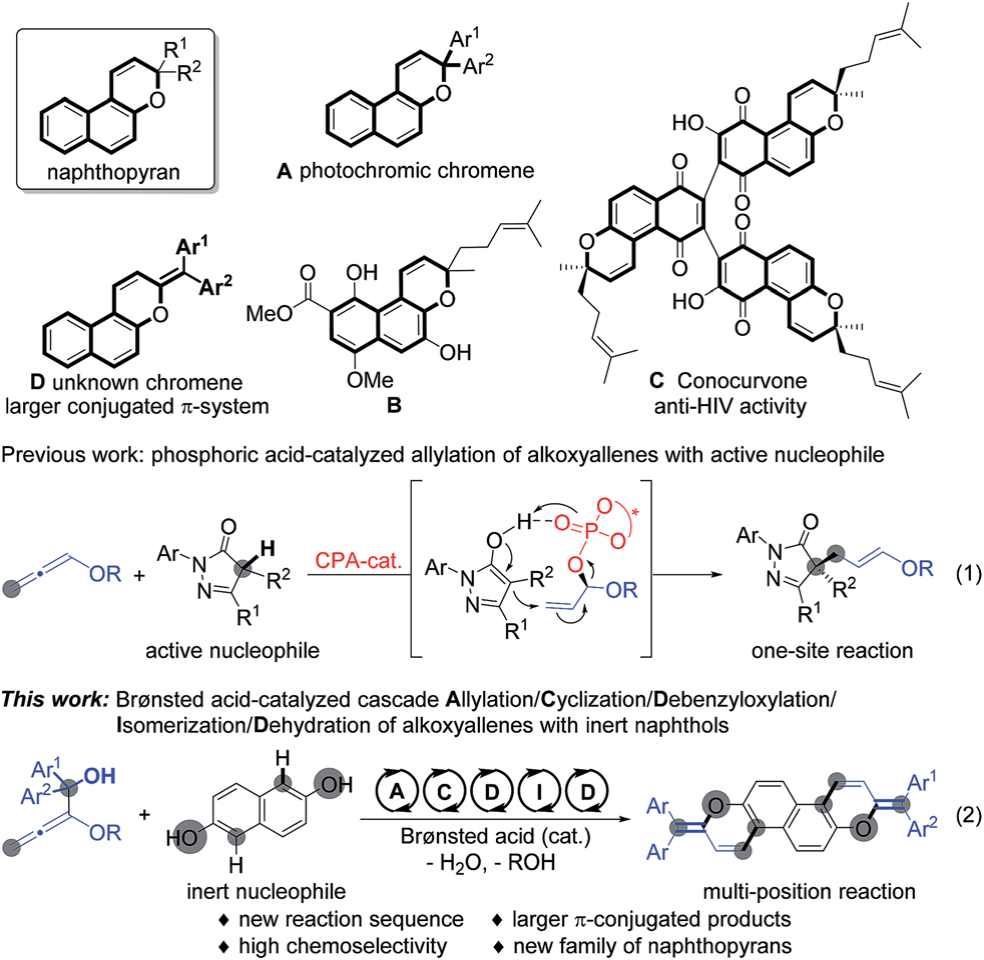
Typical naphthopyran structures and Brønsted acid-catalyzed addition of alkoxyallenes to nucleophiles.

Initially, alkoxyallene **1a** and naphthol **2a** were selected to optimize the reaction conditions (Table 1). Different Brønsted acids as the catalyst, including phosphoric acid, trifluoroacetic acid, and TsOH, gave disappointing results in 1,2-dichloroethane (DCE) at 25 °C (entries 1–3). Gratifyingly, the desired product **3aa** was isolated in 56% yield from an unidentifiable mixture in the presence of 5.0 mol% phosphoric acid by raising the reaction temperature up to 80 °C (entries 4, 5). Other solvents were detrimental to the reaction results (entries 6–9). We speculated that the temperature was too high for the allylation/cyclization steps, which led to the above unidentifiable mixture. The treatment of the reaction mixture at 25 °C for 30 minutes followed by heating at 80 °C for 8 h improved the yield up to 88% (entry 10). Increasing the loading of acid to 10.0 mol% furnished the product in almost quantitative yield (entry 11). Additionally, CF_3_CO_2_H and TsOH as the catalyst under the optimal reaction conditions gave complicated results, probably owing to their strong acidity leading to the decomposition of **1a** (entries 12, 13). As expected, no reaction occurred without an acid catalyst (entry 14).

**Table 1:** Optimization of the reaction conditions

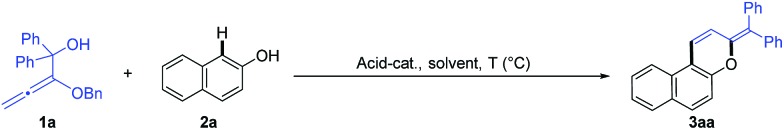				
Entry^*a*^	Acid-cat.	Solvent	*T* (°C)	Yield^*b*^
1	(PhO)_2_PO_2_H	DCE	25	Trace
2	CF_3_COOH	DCE	25	n.r.^*c*^
3	TsOH	DCE	25	n.r.^*c*^
4	(PhO)_2_PO_2_H	DCE	60	5%
5	(PhO)_2_PO_2_H	DCE	80	56%
6	(PhO)_2_PO_2_H	Toluene	80	Trace
7	(PhO)_2_PO_2_H	CH_3_CN	80	Trace
8	(PhO)_2_PO_2_H	THF	80	n.r.^*c*^
9	(PhO)_2_PO_2_H	EtOH	80	n.r.^*c*^
10^*d*^	(PhO)_2_PO_2_H	DCE	80	88%
11^*d*^ ^,^^*e*^	(PhO)_2_PO_2_H	DCE	80	98% (93%)
12^*d*^ ^,^^*e*^	CF_3_COOH	DCE	80	Complicated
13^*d*^ ^,^^*e*^	TsOH	DCE	80	Complicated
14		DCE	80	n.r.^*c*^

^*a*^Reaction conditions: to the reaction mixture of allene **1a** (0.15 mmol, 1.5 equiv.) and naphthol **2a** (0.1 mmol) in solvent (1.0 mL) was added acid (5.0 mol%), and continued to stir for 8 h.

^*b*^The yield was determined by ^1^H NMR spectroscopy.

^*c*^n.r. means no reaction.

^*d*^The reaction mixture was stirred at 25 °C for 30 min, then heated at 80 °C for 8 h.

^*e*^10.0 mol% of phosphoric acid was used; the isolated product yield is given in parentheses.

Under the optimized reaction conditions, the substrate scope with respect to both alkoxyallenes and naphthols was investigated to evaluate the generality of the reaction. As shown in Scheme 2, the reaction sequence could tolerate a wide range of naphthols. For reaction with **1a**, besides **2a**, both electron-donating and electron-withdrawing substituents at the aromatic rings are quite applicable to the optimal reaction conditions, leading to the corresponding products **3ab–an** in good yields of 50–90%. Notably, the cascade transformation can be easily scaled up to 2.0 gram scale without an appreciable decrease in product yields (**3ah**). Triphenylamine (TPA) is extensively utilized in organic electroluminescent materials, special dye synthesis, and organic solar cells.^13^ Inspiringly, compound **3ak** bearing a TPA moiety can be assembled in good yield with naphthol **2k** as the substrate. Widely studied photochromism molecule naphthopyran **2l** was also amenable to this reaction system, readily resulting in the corresponding product **3al** in 85% yield. Treatment of more challenging substrates indol-5-ol **2o** and 3,5-dimethoxyphenol **2p** into the reaction afforded the products **3ao** and **3ap** in 75% and 60% yields, respectively. The regioselectivity of **2o** took place exclusively at the 4-position. Dinaphthols could also be employed to deliver the double-annulation products **3aq–au** in acceptable yields of 58–80%. Notably, the chiral 1,1′-bi-2-naphthol (BINOL) derivative **3ar** could be obtained in 58% yield followed by “one-pot” demethylation with BBr_3_, which provided a new skeleton for the potential chiral ligand excavation.^14^ The weak disulfide bond was also compatible with the acid-catalyzed cascade process. Accordingly, the reaction of **2u** with **1a** facilely assembled the product **3au** in 76% yield. Compounds **3ae** and **3al** were characterized by X-ray crystallographic analysis.^15^ To our delight, the scope of this reaction was further extended with a series of alkoxyallenes **1b–g** and the desired products **3ba–ga** were obtained in high yields of 67–84%, no matter whether symmetric alkoxyallenes **1b–d** or asymmetric ones **1e–g** were used as the starting materials.

**Scheme 2:**
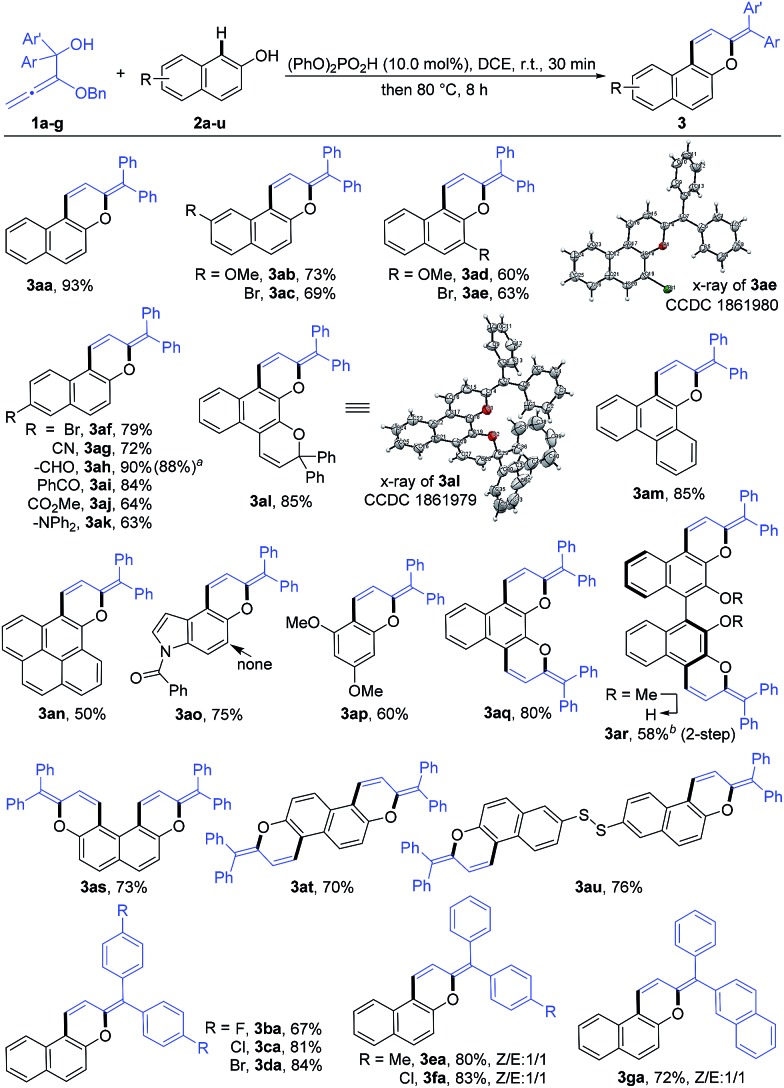
Reaction conditions: to the reaction mixture of **1** (0.75 mmol, 1.5 equiv.) and **2 **(0.5 mmol) in DCE (5 mL) was added phosphoric acid (10.0 mol%), stirred at 25 °C for 30 min, then heated at 80 °C for 8 h. Yield is that of the isolated product. ^a^2.0-gram scale. ^b^10 equiv. of **1a** was used and the yield is that of isolated 1,1′-bi-2-naphthol product after “one-pot” demethylation by BBr_3_ at room temperature.

In order to confirm the cascade reaction, a series of control reactions for the intermediates were executed. As demonstrated in Scheme 3, the initial allylation and cyclization were very fast and facile. Allylated product **4** was isolated in 63% yield by the reaction of **1a** and **2a** at 0 °C for only 5 minutes in the presence of 5.0 mol% phosphoric acid and **4** could be entirely cyclized just by warming to 25 °C for 30 minutes. Chromene **5** was also smoothly obtained in 95% yield by the direct reaction of **1a** and **2a** at the same conditions. Further increasing the temperature from 25 °C to 50 °C and heating for 20 minutes led to the reactive debenzyloxylated products **6a**/**6b** in 31% yield with a ratio of 2 : 1. Final product **3aa** was formed in quantitative yield by directly treating **5** to the optimized reaction conditions (Table 1, entry 11), and **6a**/**6b** were monitored by TLC during the reaction. The cascade reaction could be completely blocked before dehydration if the alkoxyallenes **1h**/**h′** bearing a cyclohexyl group instead of biaryl substitutes were employed.

**Scheme 3:**
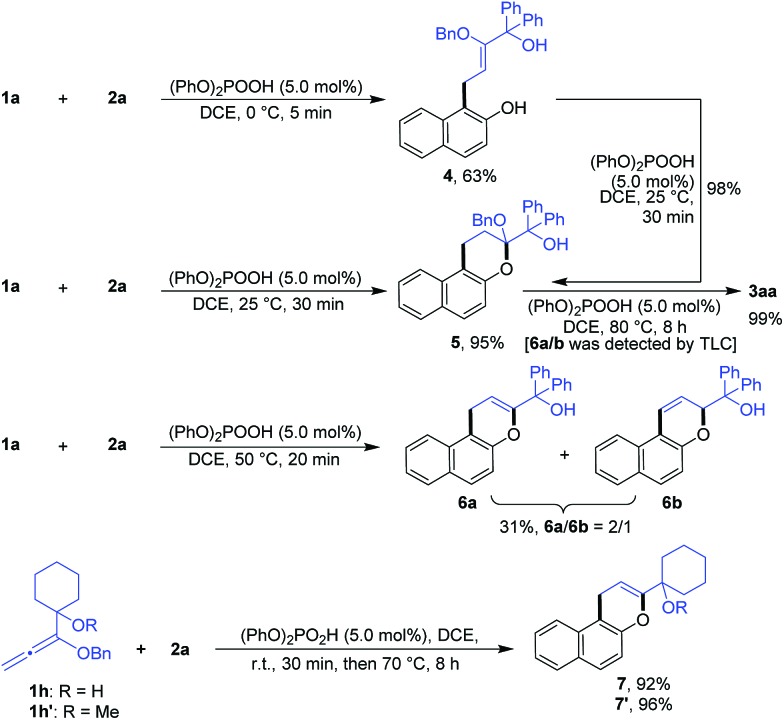
Investigation of intermediates.

These findings strongly support the reaction sequence *via* allylation/cyclization/debenzyloxylation/isomerization/dehydration with high efficiency (Scheme 4).

**Scheme 4:**
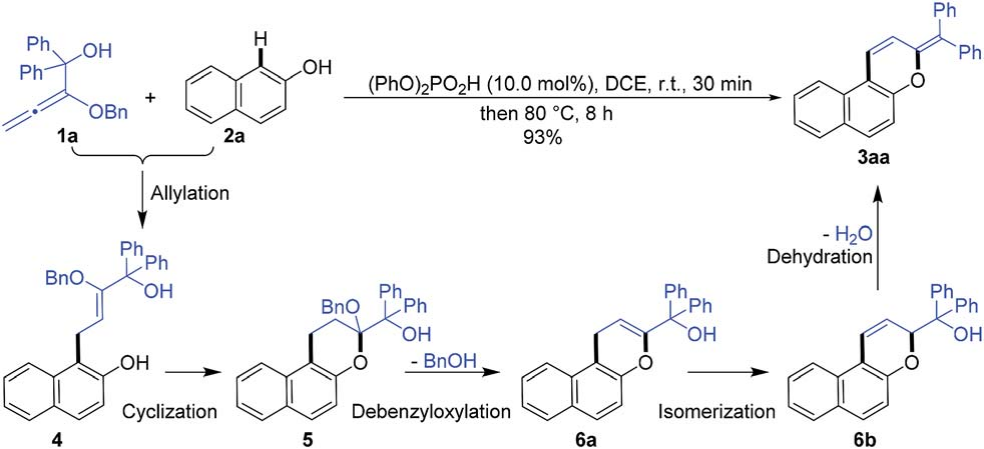
Proposed reaction sequence.

As a new family of larger π-conjugated naphthopyrans was obtained, we went on to investigate their photophysical properties. The absorptions and emissions were measured in the solid state under nitrogen at ambient temperature. With regard to the exemplified compounds **3ac**, **3ad**, **3ba** and **3da**, there are overall three absorption bands that are common in both solution and solid states (Table 2 and Fig. S1†). The strong absorption bands below 300 nm are normally assigned to local π–π* transitions of aryl rings. Bands in the 300–400 nm region are from the composited π systems corresponding to the naphthopyran^16^ or diphenylmethylene moiety,^17^ separately. The last absorption bands at 400–500 nm are assigned to the whole conjugated molecule system. Remarkably, those compounds exhibit solid-state photoluminescence in the visible region from yellow to red (Fig. 1). Compounds **3ae** and **3al**, involving similar half-side structure of 2-(diphenylmethylene)-2*H*-chromene with two pendent phenyls staggered with each other (Scheme 2, see crystal structures), have similar absorption and emission vibronic structures in their spectra (Fig. S1†). However, **3al** emits red light, whereas **3ae** gives red-color emission (Fig. 1). Those results showed that the emission colors can be well tuned *via* increasing π conjugation at the benzopyran site. Furthermore, compound **3aq** emits light in the near-infrared range with a peak at 660 nm, which indicates a distinct packing morphology (Fig. S1†).^18^ The decaying lifetimes, measured *via* transient spectrometer, were a few tenths of nanoseconds to a few nanoseconds, indicating a typical fluorescence feature rather than a phosphorescent decay process (Table 2 and Fig. S2†).

**Table 2:** Photophysical properties of selected compounds

Compound	*λ*_abs_ ^*a*^ (nm)	*λ*_em_ ^*b*^ (nm)	*τ* ^*c*^ (ns)
**3al**	252/290/366/464	604	3.1
**3an**	273/358/473	557	0.99
**3ao**	326/443	533	0.58
**3at**	220/290/372/498	603	1.37
**3ba**	273/349/432	587	2.53

^*a*^Absorption peaks (*λ*_abs_) were measured in neat film at room temperature.

^*b*^Emission peaks were measured in powdery samples at room temperature.

^*c*^Decaying lifetimes (*τ*) of these emitters in neat films.

**Fig. 1:**
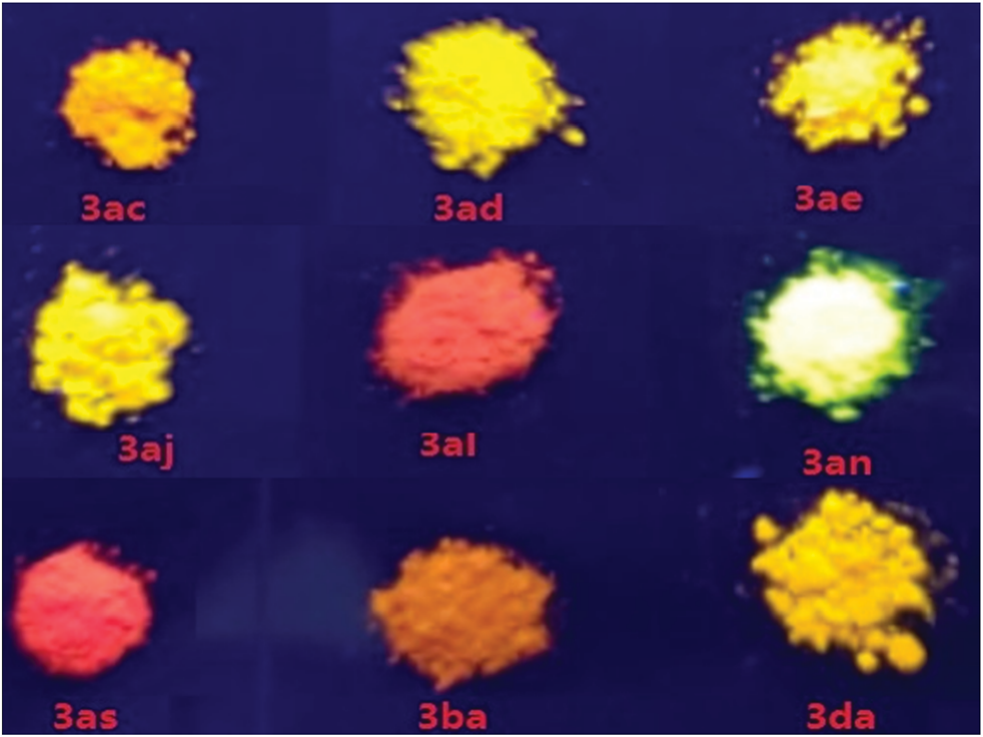
Selected illuminant compounds under UV irradiation at 365 nm.

Unlike the photochromic character of naphthopyrans,3*f* those compounds are inert to UV light irradiation and no structural or conformational variation was found on **3an** and **3at** when exposed under the intensity of 12 μW cm^2^ 365 nm UV light over 10 hours. This should be ascribed to the stabilization effects of p–π hyperconjugation from the neighbouring diphenylmethylene group to the photosensitive C–O bonds, since natural naphthopyran^3^ structures involving a non-conjugated quaternary carbon centre are highly sensitive to light and heat.^19^ Comparing to their almost non-luminance in homogeneous THF solution, we deemed that those light emissive compounds have the AIE feature in conformity with the mechanism of the AIE molecule tetraphenylethene (Fig. 2), since both have diarylmethylene AIEgens.^20^**3an** and **3at** shined bright yellow and orange light under lighting from a 365 UV lamp, and became brighter with more aggregates in well dispersed suspension. The solution-suspension luminescent efficiency *Φ*_F_s of **3an** in 1 : 8 THF : H_2_O (v/v) and **3at** in 1 : 1.7 THF : H_2_O (v/v) are 1.9% and 2.3%, respectively. The results exhibit distinct photophysical functions compared to the natural naphthopyran molecules.

**Fig. 2:**
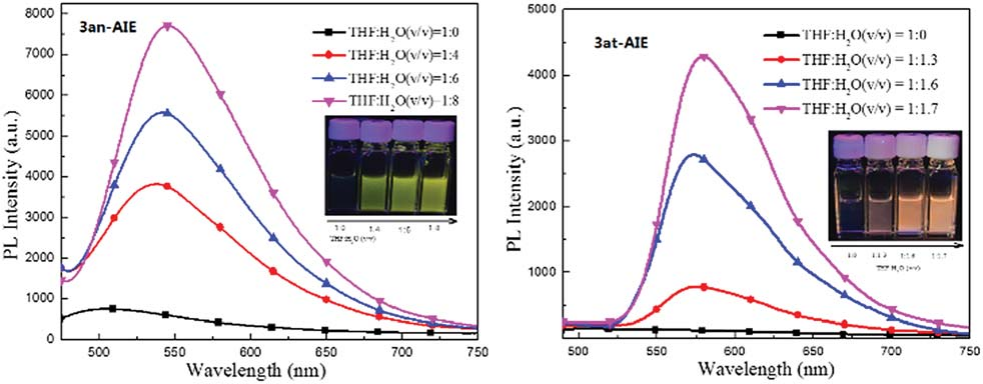
Photoluminescence spectra of **3an** (left) and **3at** (right) in THF/H_2_O mixtures.

## Conclusions

In summary, we developed a practical and readily scalable reaction sequence for the straightforward synthesis of a new family of larger π-conjugated naphthopyrans by a Brønsted acid-catalyzed aromatic annulation of alkoxyallenes with inert naphthols. The cascade pathway involves allylation/cyclization/debenzyloxylation/isomerization/dehydration. The new class of diphenylmethylene substituted naphthopyrans was demonstrated to be AIEgenic with luminescent behavior in solid state. Those compounds are quite stable to UV light irradiation, which is promising for their potential utilization for long term and precise tracking. Given the importance of naphthopyrans in natural products and biocompatible photochromic materials, the method reported herein is of high importance as it provides access to hitherto unknown larger π-conjugated naphthopyran compounds whose optical properties investigation and underlying exploitation are highly warranted.

## Conflicts of interest

There are no conflicts to declare.

## Supplementary Material

Supplementary information

Crystal structure data
