# Supplementary material for: Brønsted acid-catalyzed aromatic annulation of alkoxyallenes with naphthols: a reaction sequence to larger π-conjugated naphthopyrans with aggregation-induced emission characters
Source: Chem Sci. 2018 Nov 2;10(4):1070–4. doi: 10.1039/c8sc03837f (PMC6346396; doi:10.1039/c8sc03837f)

Electronic Supplementary Material (ESI) for Chemical Science.
This journal is © The Royal Society of Chemistry 2018

Supporting Information for

Brønsted Acid-Catalyzed Aromatic Annulation of Alkoxyallenens with Naphthols: A Reaction Sequence to Larger π-Conjugated Naphthopyrans with Aggregation-Induced Emission Characters

Jinlong Zhang,[a] Lu Zhu,[b] Kang Shen,[b] Huameng Yang,[a] Xiao-Chun Hang,[b]* Gaoxi Jiang[a]*

[a] *State Key Laboratory for Oxo Synthesis and Selective Oxidation, Center for Excellence in Molecular Synthesis, Suzhou Research Institute of LICP, Lanzhou Institute of Chemical Physics (LICP), Chinese Academy of Sciences, Lanzhou 730000, China*

[b] *Key Laboratory of Flexible Electronics (KLOFE) and Institute of Advanced Materials (IAM), Nanjing Tech University (NanjingTech), 30 South Puzhu Road, Nanjing 211800, China*

X.-C., H.: iamxchhang@njtech.edu.cn

G., J.: gxjiang@licp.cas.cn

Table of Contents

Materials and Methods……………………………...………..…………………………………………S1

Synthesis of Starting Materials…………………………………………………………….…………………S2

General procedure for Brønsted acid-catalyzed aromatic annulation………………………….…………..S10

Transient decay curves, Absorption and emission spectra of selected compounds……………..……..S11

Characterization of the products…………………………………...……………………..…………S13

Investigation of Intermediates……………………………………………..……………….…..…………S22

X-Ray crystallographic determination of compounds **3ae** and **3al**……………………………….………...S26

NMR spectra…………………...……………………………………………………..…………S28

Materials and Methods

Experimental: All non-aqueous manipulations were using standard Schlenk techniques. All reactions were set up under air. Reactions were monitored using thin-layer chromatography (TLC) on silica gel plates. Visualization of the developed plates were performed under UV light (254 nm) or $KMnO_4$ stain. Silica gel flash column chromatography was performed on SYNTHWARE 40-63 μm silica gel.

Instrumentation: All NMR spectra were run at 400 MHz (^1H NMR) or 100 MHz (^{13}C NMR) in $CDCl_3$. ^1H NMR spectra were internally referenced to TMS. ^{13}C NMR spectra were internally referenced to the residual solvent signal. Data for ^1H NMR are reported as follows: chemical shift (δ ppm), multiplicity (s = singlet, d = doublet, t = triplet, q = quartet, m= multiplet, br = broad), coupling constants (*J*) were reported in Hz. High resolution mass spectra (HRMS) were recorded on Bruker MicrOTOF-QII mass instrument (ESI).

Materials: Unless otherwise indicated, starting catalysts and commercially available reagents were used without additional purification.

Synthesis of Starting Materials

1. Preparation of 2-(Benzyloxy)-1,1-diarylbuta-2,3-dien-1-ol

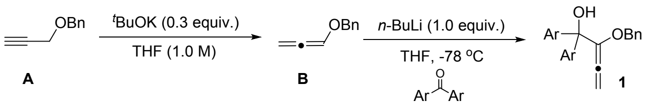

(1st step) To a solution of benzyl prop-2-yn-1-ol **A** (2.9 g, 20.0 mmol) in THF (20 mL) was added potassium *tert*-butanol (6.0 mmol) and stirred at room temperature for 4 h. After the completion of the reaction determined by TLC, the reaction mixture was filtered through a celite pad and washed with 60 ml of Et_2O. The combined solution was concentrated in vacuo and purified by silica gel chromatography (1% Et_2O in petroleum ether). Allene **B** (81%, 2.37 g, 16.2 mmol) was obtained as a light yellowish liquid.

(2nd step) Allene **B** (0.73g, 5.0 mmol) in THF (5 mL) was cooled down to -78 °C, *n*-BuLi (2.5 M in hexane, 2 mL, 5.0 mmol) was added dropwise and continue to stir for 1 h. Then ketone (3.4 mmol) solvent in THF (5 mL) was dropped into the reaction mixture and stirred for another 2 h. After the completion of the reaction determined by TLC. The reaction mixture was quenched with saturated NH_4Cl solution and extracted with Et_2O for three times. The combined organic layer was dried over Na_2SO_4, and the organic solvent was removed *in vacuo*. The residue was purified by flash column chromatography on silica gel to afford product **1** (60-80% yield).

2-(Benzyloxy)-1,1-bis(4-phenyl)buta-2,3-dien-1-ol (1a)

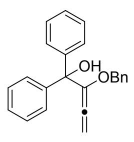

^{1}H NMR (400 MHz, $CDCl_3$) δ 7.45 – 7.35 (m, 4H), 7.35 – 7.14 (m, 11H), 5.32 (d, J = 1.2 Hz, 2H), 4.72 (s, 2H), 3.38 (d, J = 1.3 Hz, 1H); ^{13}C NMR (100 MHz, $CDCl_3$) δ 198.36, 144.06, 136.96, 128.27, 127.86, 127.86, 127.62, 127.58, 127.34, 92.99, 80.12, 71.18. HRMS (ESI+): m/z Calcd. for $C_{26}H_{21}O$ $[M+H]^+$: 329.1542. Found: 329.1545.

2-(Benzyloxy)-1,1-bis(4-fluorophenyl)buta-2,3-dien-1-ol (1b)

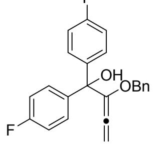

^{1}H NMR (400 MHz, $CDCl_3$) δ 7.39 – 7.27 (m, 7H), 7.26 – 7.18 (m, 2H), 7.03 – 6.83 (m, 4H), 5.33 (s, 2H), 4.71 (s, 2H), 3.40 (s, 1H); ^{13}C NMR (100 MHz, $CDCl_3$) δ 198.00, 163.36, 160.91, 139.71, 136.68, 135.78, 129.38, 129.30, 128.35, 128.02, 127.94,

114.54, 93.31, 79.40, 71.27. HRMS (ESI+): m/z Calcd. for $C_{26}H_{19}F_2O$ $[M+H]^+$: 365.1353. Found: 365.1357.

2-(Benzyloxy)-1,1-bis(4-chlorophenyl)buta-2,3-dien-1-ol (1c)

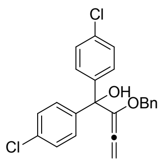

^1H NMR (400 MHz, $CDCl_3$) δ 7.33 – 7.27 (m, 7H), 7.26 – 7.18 (m, 6H), 5.35 (s, 2H), 4.70 (s, 2H), 3.42 (s, 1H); ^{13}C NMR (100 MHz, $CDCl_3$) δ 197.86, 142.21, 133.40, 128.97, 128.36, 128.29, 128.06, 127.96, 127.82, 127.40, 93.63, 79.39, 71.31. HRMS (ESI+): m/z Calcd. for $C_{26}H_{19}Cl_2O$ $[M+H]^+$: 397.0762. Found: 397.0768.

2-(Benzyloxy)-1,1-bis(4-bromophenyl)buta-2,3-dien-1-ol (1d)

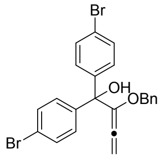

^1H NMR (400 MHz, $CDCl_3$) δ 7.45 – 7.36 (m, 4H), 7.33 – 7.27 (m, 3H), 7.25 – 7.10 (m, 6H), 5.35 (s, 2H), 4.69 (s, 2H), 3.43 (s, 1H); ^{13}C NMR (100 MHz, $CDCl_3$) δ 197.78, 142.63, 136.48, 135.04, 130.77, 129.30, 128.35, 128.06, 127.97, 121.67, 93.70, 79.46, 71.29. HRMS (ESI+): m/z Calcd. for $C_{26}H_{19}Br_2O$ $[M+H]^+$: 484.9752. Found: 484.9756.

2-(benzyloxy)-1-phenyl-1-(p-tolyl)buta-2,3-dien-1-ol (1e)

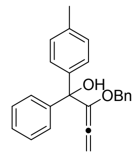

^1H NMR (400 MHz, $CDCl_3$) δ 7.46 – 7.35 (m, 2H), 7.32 – 7.18 (m, 10H), 7.12 – 7.03 (m, 2H), 5.32 (d, *J* = 1.1 Hz, 2H), 4.71 (s, 2H), 3.35 (d, *J* = 1.6 Hz, 1H), 2.32 (s, 3H); ^{13}C NMR (100 MHz, $CDCl_3$) δ 198.32, 144.20, 141.15, 136.99, 136.94, 128.30, 128.24, 127.84, 127.80, 127.52, 127.25, 92.94, 79.95, 71.14, 21.09. HRMS (ESI+): m/z Calcd. for $C_{26}H_{27}O_2$ $[M+H]^+$: 371.2011, Found: 371.2019.

2-(benzyloxy)-1-(4-chlorophenyl)-1-phenylbuta-2,3-dien-1-ol (1f)

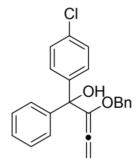

^1H NMR (400 MHz, $CDCl_3$) δ 7.42 – 7.35 (m, 2H), 7.35 – 7.08 (m, 12H), 5.31 (s, 2H), 4.69 (s, 2H), 3.44 (s, 1H); ^{13}C NMR (100 MHz, $CDCl_3$) δ 198.06, 143.65, 142.57, 136.70, 135.67, 133.10, 129.06, 128.26, 127.90, 127.87, 127.69, 127.62, 127.51, 127.45, 93.27, 79.71, 71.19. HRMS (ESI+): m/z Calcd. for $C_{25}H_{25}ClO_2$ $[M+H]^+$: 391.1464, Found: 391.1470.

2-(Benzyloxy)-1-(naphthalen-2-yl)-1-phenylbuta-2,3-dien-1-ol (1g)

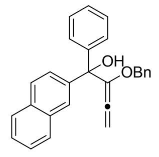

^{1}H NMR (400 MHz, $CDCl_3$) δ 7.88 – 7.71 (m, 4H), 7.54 – 7.49 (m, 1H), 7.47 – 7.41 (m, 4H), 7.31 – 7.19 (m, 8H), 5.33 (s, 2H), 4.75 (s, 2H), 3.51 (s, 1H); ^{13}C NMR (100 MHz, $CDCl_3$) δ 198.29, 143.87, 141.45, 136.88, 135.93, 132.73, 132.68, 128.37, 128.28, 127.94, 127.87, 127.69, 127.62, 127.42, 127.15, 126.18, 126.12, 125.93, 125.78, 93.24, 80.24, 71.18. HRMS (ESI+): m/z Calcd. for $C_{27}H_{23}O_2$ $[M+H]^+$: 379.1698. Found: 379.1698.

1-(1-(benzyloxy)propa-1,2-dien-1-yl)cyclohexan-1-ol (1h)

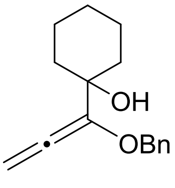

^{1}H NMR (400 MHz, $CDCl_3$) δ 7.42 – 7.21 (m, 5H), 5.53 (d, J = 2.2 Hz, 2H), 4.62 (d, J = 2.2 Hz, 2H), 2.19 (s, 1H), 1.74 – 1.59 (m, 10H). ^{13}C NMR (100 MHz, $CDCl_3$) δ 197.02, 137.54, 128.25, 128.19, 127.63, 127.52, 126.81, 92.38, 70.51, 35.45, 25.57, 22.04. HRMS (ESI+): m/z Calcd. for $C_{16}H_{21}O_2$ $[M+H]^+$: 245.1542, Found: 245.1548.

(((1-(1-methoxycyclohexyl)propa-1,2-dien-1-yl)oxy)methyl)benzene (1h')

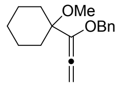

^{1}H NMR (400 MHz, $CDCl_3$) δ 7.41 – 7.10 (m, 5H), 5.47 (s, 2H), 4.65 (s, 2H), 3.15 (s, 3H), 1.84 – 1.70 (m, 2H), 1.67 – 1.53 (m, 4H), 1.50 – 1.21 (m, 4H); ^{13}C NMR (100 MHz, $CDCl_3$) δ 199.95, 137.88, 128.12, 127.49, 127.41, 91.21, 70.20, 49.56, 32.61, 25.74, 21.99. HRMS (ESI+): m/z Calcd. for $C_{17}H_{23}O_2$ $[M+H]^+$: 259.1698, Found: 259.1695.

2. *Preparation of (5-Hydroxy-1H-indol-1-yl)(phenyl)methanone*

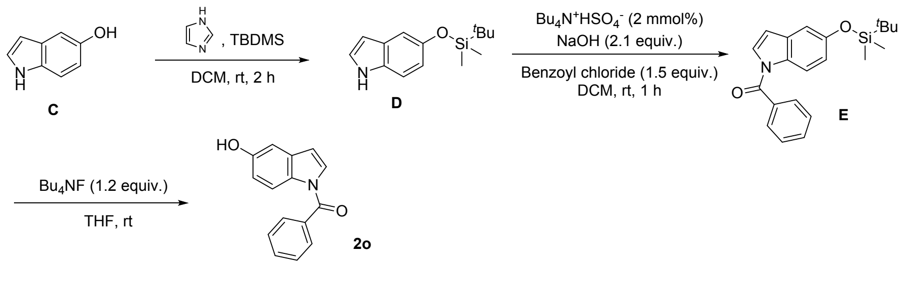

([1st] step) The reaction mixture of indol-5-ol **C** (1.33 g, 10.0 mmol), imidazole (1.64 g, 24.0 mmol) and tertbutyldimethylchlorosilane (1.74 g, 14.4 mmol) in DCM (40 mL) was stirred at room temperature for 2 h. After the completion of the reaction determined by TLC, the reaction mixture was added 10 mL of water and extracted with EtOAc for three times. The combined organic layer was dried over Na_2SO_4,

and the organic solvent was removed *in vacuo*. the residue was purified by flash column chromatography using hexane as eluents to afford protected indol-5-ol **D** (89%, 2.20 g, 8.9 mmol) as a brown liquid.

(2nd step) Indol-5-ol **D** (1.24 g, 5.0 mmol), *n*-$Bu_4N^+HSO_4^-$ (13.8 mg, 0.01 mmol) and powered NaOH (0.42 g, 10.5 mmol) were dissolved in DCM (10 mL). Then benzoyl chloride (1.05 g, 7.5 mmol) in DCM (5 mL) was added dropwise to the vigorously stirring solution. After 2 h at room temperature, TLC showed complete consumption of the starting indole. The reaction mixture was added 40 mL of water and extracted with DCM for three times. The combined organic layers were washed with brine, dried over Na_2SO_4, and concentrated under reduced pressure. The resulting material was subjected to flash column chromatography on silica gel to afford silicon protected benzoyl indol-5-ol **E** (78%, 1.37 g, 3.9 mmol) as a solid.

(3rd step) To a solution of indole **E** (1.05 g, 3.0 mmol) in THF (5 mL) was added dropwise tetrabutylammonium fluoride (1.0 M in THF, 3.6 mL, 3.6 mmol) solution, and the reaction mixture was stirred at ambient temperature for 1 h. The solvent was removed *in vacuo*, the residue was purified by flash column chromatography to afford benzoyl indol-5-ol **2o** (97%, 0.69 g, 2.9 mmol) as a white solid. ^1H NMR (400 MHz, $CDCl_3$) δ 9.36 (d, *J* = 1.6 Hz, 1H), 8.09 (d, *J* = 8.8 Hz, 1H), 7.76 – 7.67 (m, 2H), 7.66 – 7.60 (m, 1H), 7.59 – 7.50 (m, 2H), 7.23 (d, *J* = 3.7 Hz, 1H), 6.96 (d, *J* = 2.2 Hz, 1H), 6.81 (dt, *J* = 8.9, 1.9 Hz, 1H), 6.58 (d, *J* = 3.7 Hz, 1H); ^{13}C NMR (100 MHz, $CDCl_3$) δ 167.77, 154.20, 134.24, 131.87, 131.79, 129.17, 128.84, 128.69, 128.48, 116.57, 113.51, 108.48, 105.77, 40.13, 39.92, 39.71, 39.50, 39.29, 39.08, 38.87. HRMS (ESI+): m/z Calcd. for $C_{15}H_{12}NO_2$ $[M+H]^+$: 238.0868, Found: 238.0866.

3. *Preparation of 6-(Diphenylamino)naphthalen-2-ol*

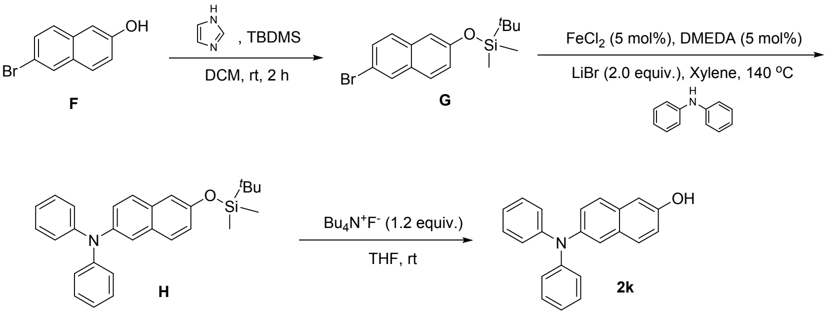

(1st step) The reaction mixture of 6-bromonaphthalen-2-ol **F** (1.33 g, 6.0 mmol), imidazole (0.82 g, 12.0 mmol) and tertbutyldimethylchlorosilane (0.87 g, 7.2 mmol) in DCM (20 mL) was stirred at room

temperature for 2 h. After the completion of the reaction determined by TLC, the reaction mixture was added 10 mL of water and extracted with EtOAc for three times. The combined organic layer was dried over Na_2SO_4, and the organic solvent was removed *in vacuo*. the residue was purified by flash column chromatography using hexane as eluents to afford protected 6-bromonaphthalen-2-ol **G** (92%, 1.86 g, 5.5 mmol) as a solid.

(2nd step)[1] To a solution of diphenylamine (1.69 g, 10 mmol) in Et_2O (10 mL) was added a solution of MeMgBr (0.5 M in Et_2O, 20 mL,10 mmol) at 0 ^oC. After stirring at 40 ^oC for 2 h, the solvent removed under reduced pressure. A flame dried Schlenk flask was charged with $FeCl_2$ (6.4 mg, 0.05 mmol), LiBr (1.7 g, 20 mmol), protected 6-bromonaphthalen-2-ol **G** (1.68 g, 5 mmol), dimethylethanediamine (DMEDA, 4.4 mg, 0.05 mmol) magnesium amide prepared above and xylene (20 mL), and the resulting mixture was stirred at 140 ^oC for 30 h. The reaction mixture was quenched with saturated NH_4Cl solution and extracted with Et_2O for three times. The combined organic layer was dried over Na_2SO_4, and the organic solvent was removed under reduced pressure. The residue was purified by flash column chromatography on silica gel with hexane / EtOAc (v / v = 100 / 1) as eluents to afford silicon protected 6-diphenylaminonaphthalen-2-ol **H** (74%, 1.57 g, 3.7 mmol) as a white solid.

(3rd step) To a solution of silicon protected 6-diphenylaminonaphthalen-2-ol **H** (1.28 g, 3.0 mmol) in THF (5 mL) was added dropwise tetrabutylammonium fluoride (1.0 M in THF, 3.6 mL, 3.6 mmol) solution, and the reaction mixture was stirred at ambient temperature for 1 h. The solvent was removed *in vacuo*, the residue was purified by flash column chromatography using hexane / EtOAc (v / v = 20 / 1) as eluents to afford 6-(diphenylamino)naphthalen-2-ol **2k** (98%, 0.91 g, 2.9 mmol) as a slurry solid. ^1H NMR (400 MHz, $CDCl_3$) δ 7.56 (d, J = 8.8 Hz, 1H), 7.50 (d, J = 8.8 Hz, 1H), 7.40 (s, 1H), 7.29 – 7.18 (m, 5H), 7.15 – 7.06 (m, 5H), 7.05 – 6.96 (m, 3H), 5.10 (s, 1H). HRMS (ESI+): m/z Calcd. for $C_{22}H_{18}NO$ $[M+H]^+$: 312.1388, Found: 312.1388.

4. *Preparation of 3,3-Diphenyl-3H-benzo[f]chromen-5-ol*

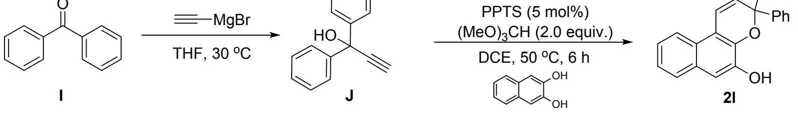

(1st step)[2] Ethynylmagnesium bromide (0.5 mol/L in THF, 24 mL, 1.2 equiv) was added dropwise into a stirred solution of benzophenone **I** (10 mmol, 1.82 g) in THF (20 mL) under argon at 0 ^oC. Then

the mixture was allowed to stir for 4h at 30 °C. After the completion of the reaction determined by TLC, the reaction mixture was quenched by addition of an aqueous saturated solution of NH_4Cl (30 mL) and extracted with ethyl acetate (3×50 mL). The combined organic layers were washed with brine, dried over Na_2SO_4, and concentrated under reduced pressure. The resulting material was subjected to flash column chromatography on silica gel to afford 1,1-diphenylprop-2-yn-1-ol **J** (91%, 1.89 g, 9.1 mmol) as a gray solid.

(2nd step) Naphthalene-2,3-diol (320 mg, 2.0 mmol) and 1,1-diphenylprop-2-yn-1-ol **J** (416 mg, 2 mmol, 1.0 equiv.) was stirred in the presence of PPTS (0.1 mmol, 5 mol%) and trimethyl orthoformate (4 mmol, 2.0 equiv.) in 1,2-dichloroethane (10 ml) at 50 °C for 6 h. After the completion of the reaction determined by TLC, solvent was removed in vacuo, the residue was purified by chromatography to afford the desired compound 3,3-diphenyl-3H-benzo[*f*]chromen-5-ol **2l** (75%, 525 mg, 1.5 mmol) as a solid. (W. Zhao and E. M. Carreira, *Org. Lett.* **2003**, *5*, 4153-4154.) ^{1}H NMR (400 MHz, $CDCl_3$) δ 7.86 (d, *J* = 8.0 Hz, 1H), 7.62 – 7.55 (m, 1H), 7.45 – 7.38 (m, 4H), 7.35 – 7.26 (m, 9H), 7.20 (s, 1H), 6.22 (dd, *J* = 10.0, 1.4 Hz, 1H), 5.90 (d, *J* = 1.7 Hz, 1H); ^{13}C NMR (100 MHz, $CDCl_3$) δ 144.20, 143.79, 139.78, 129.75, 128.23, 127.94, 127.78, 127.19, 126.99, 124.67, 124.39, 124.18, 121.17, 119.48, 114.36, 110.81, 83.87.

5. *Preparation of (S)-2,2'-dimethoxy-[1,1'-binaphthalene]-3,3'-diol*

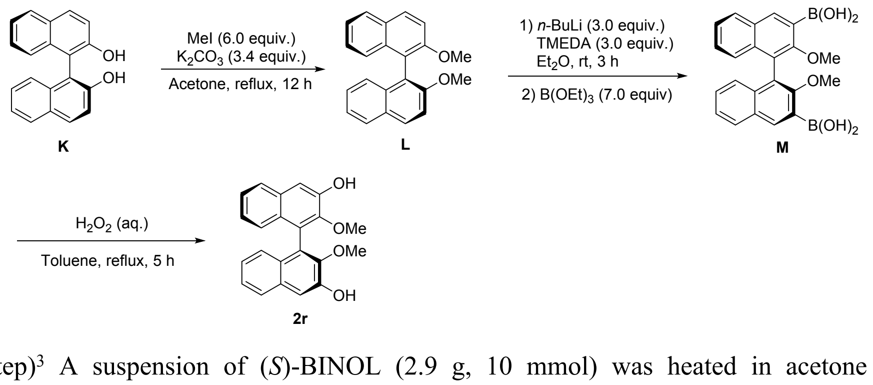

(1st step)[3] A suspension of (*S*)-BINOL (2.9 g, 10 mmol) was heated in acetone to give a homogeneous solution. To this solution was added potassium carbonate (4.7 g, 34 mmol) and methyl iodide (3.7 mL, 60 mmol), and the mixture was heated under reflux conditions for 12 h. The solvent was evaporated to leave a volume of 50 mL, which was cooled to room temperature and treated with 30 mL of water. The mixture was stirred for 8 h, and the resulting solid was washed with water and dried to afford (R)-2,2'-dimethoxy-1,1'-dinaphthyl **L** (3.0 g, 95%) as a white powder.

(2nd step) To a solution of tetramethylethylenediamine (1.8 mL, 12 mmol) in ether (60 mL) was added *n*-BuLi (2.5 M in hexane, 8.8 mL, 12 mmol) at room temperature. The solution was stirred for 30 min, solid (*S*)-2,2'-dimethoxy-1,1'-dinaphthyl **L** (1.3 g, 4 mmol) was added in one portion, and the reaction mixture was stirred for 3 h. The resulting light brown suspension was cooled to –78 °C, and triethyl borate (4.8 mL, 28 mmol) was added over a period of 10 min. The solution was allowed to warm to room temperature and stirred for 12 h. The reaction mixture was cooled to 0 °C, and 1 M HCl solution (300 mL) was added, and the resulting solution was stirred at room temperature for 2 h. The organic layer was washed with 1M HCl solution and brine, dried over Na_2SO_4, and concentrated in vacuo. The resulting pale yellow solid was purified by chromatography to afford the desired (*S*)-3,3'-bis(dihydroxyborane)-2,2'-dimethoxy-1,1'-dinaphthyl **M** (1.5 g, 91%).

(3rd step) To a solution of (*S*)-3,3'-bis(dihydroxyborane)-2,2'-dimethoxy-1,1'-dinaphthyl **M** (0.8 g, 2.0 mmol) in toluene (20 mL) was added 30% H_2O_2 aq (0.6 mL) at 0 °C. The mixture was refluxed for 5 h. After allowed to room temperature, the mixture was poured into sat. Na_2SO_3 aq. (ca. 15 mL), and extracted by EtOAc three times. The combined organic layers were washed with brine, and dried over Na_2SO_4. After evaporation, the residue was purified by chromatography to afford the desired compound **2r** (86%, 0.6 g, 1.7 mmol) as a purple solid. ^1H NMR (400 MHz, $CDCl_3$) δ 7.75 (d, *J* = 8.2 Hz, 2H), 7.52 – 7.45 (m, 2H), 7.39 – 7.29 (m, 2H), 7.18 – 7.04 (m, 4H), 6.39 (s, 2H), 3.32 (d, *J* = 1.4 Hz, 6H); ^{13}C NMR (100 MHz, $CDCl_3$) δ 147.71, 146.17, 131.37, 128.76, 126.68, 125.55, 125.43, 124.16, 122.96, 110.68, 60.91. HRMS (ESI+): m/z Calcd. for $C_{22}H_{19}O_4$ $[M+H]^+$: 347.1283. Found: 347.1286.

1. Hatakeyama, T.; Imayoshi, R.; Yoshimoto, Y.; Ghorai, S. K.; Jin, M.; Takaya, H.; Norisuye, K.; Sohrin, Y.; Nakamura, M. *J. Am. Chem. Soc.* **2012**, *134*, 20262-20265.
2. Qiu, Y.-F.; Song, X.-R.; Li, M.; X.-Y. Zhu, A.-Q. Wang, F. Yang, Y.-P. Han, H.-R. Zhang, D.-P. Jin, Y.-X. Li and Y.-M. Liang, *Org. Lett.* **2016**, *18*, 1514-1517.
3. (a) Hatano, M.; Horibe, T.; Ishihara, K. *J. Am. Chem. Soc.* **2010**, *132*, 56-57; (b) Danjo, H.; Hirata, K.; Yoshigai, S.; Azumaya, I.; Yamaguchi, K. *J. Am. Chem. Soc.* **2009**, *131*, 1638-1639.

General procedure for Brønsted acid-catalyzed aromatic annulation

General procedure for compounds **3aa-ap**, **3ba-ga**

To the solution of allene **1** (0.75 mmol, 1.5 equiv.) and naphthol **2** (0.5 mmol) in DCE solvent (5.0 mL) was added phosphoric acid (10.0 mol%). After stirring at 25 °C for 30 min, the reaction mixture was heated to 80 °C and the stirring continued for another 8 h. Then the organic solvent was removed *in vacuo*. the residue was purified by flash column chromatography using hexane/EtOAc as eluents to afford the desired products **3aa-ap**, **3ba-ga** as a colored solid covering from yellow to purple.

General procedure for compounds **3aq**, **3as-au**

To the solution of allene **1** (1.5 mmol, 3.0 equiv.) and naphthol **2** (0.5 mmol) in DCE solvent (5.0 mL) was added phosphoric acid (10.0 mol%). After stirring at 25 °C for 30 min, the reaction mixture was heated to 80 °C and the stirring continued for another 8 h. Then the organic solvent was removed *in vacuo*. the residue was purified by flash column chromatography using hexane/EtOAc or hexane/EtOAc/DCM to afford the desired products**3aq**, **3as-au** as a colored solid. The desired product can be further purified through crystallization in mixture solvent of methanol and DCM via the volatilization of DCM.

General procedure for compound **3ar**

To the solution of allene **1** (2.0 mmol, 10.0 equiv.) and naphthol **2r** (0.2 mmol) in DCE solvent (2.0 mL) was added phosphoric acid (10.0 mol%). After stirring at 25 °C for 30 min and at 80 °C for 8 h, TsOH (10.0 mol%) was added to the reaction mixture and stirred for another 30 min. Then organic solvent was removed *in vacuo*. the residue was purified by flash column chromatography using EtOAc/DCM (50:1) to afford the methyl protected **3ar** containing some un-separable byproduct. The crude product was used directly to the next step. The methyl protected **3ar** in DCE was added 1.0 M DCE solution of BBr_3 (0.4 mL, 0.4 mmol) at 0 °C, After stirring at 25 °C for 6 h, the reaction mixture was quenched with saturated NH_4Cl solution and extracted with Et_2O for three times. The combined organic layer was dried over $MgSO_4$, and the organic solvent was removed under reduced pressure. The residue was purified by flash column chromatography on silica gel with EtOAc/DCM (50:1 to 20:1) to afford the desired product **3ar**

as a red solid in a yield of 58%.

Transient decay curves, Absorption and emission spectra of selected compounds

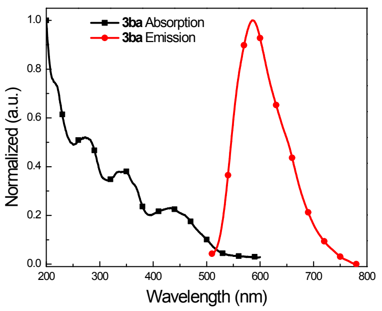

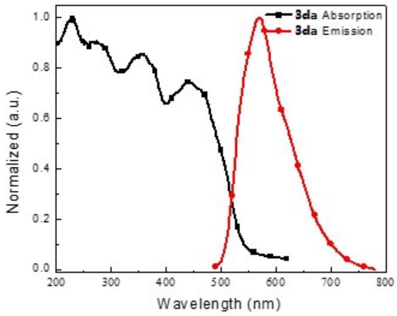

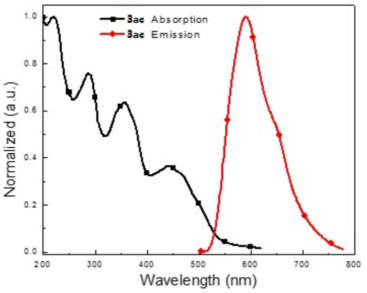

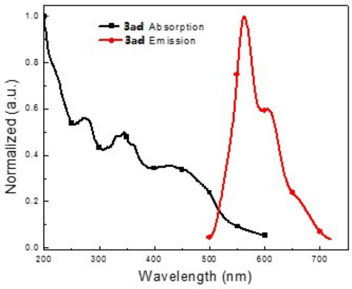

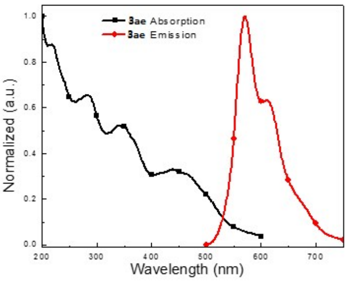

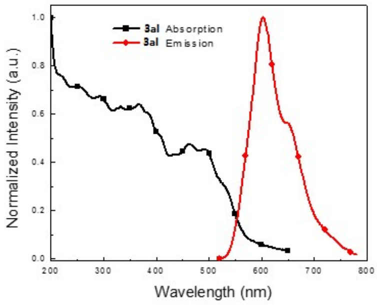

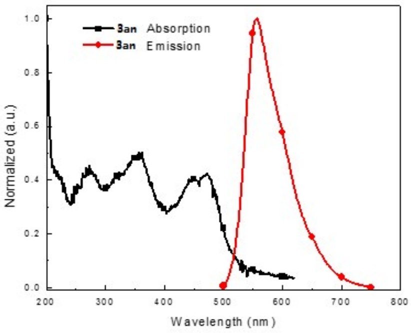

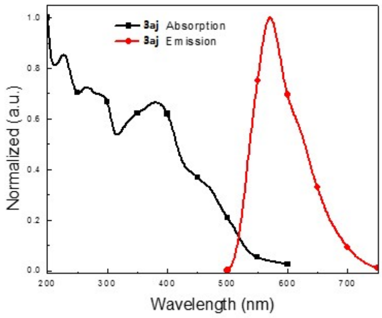

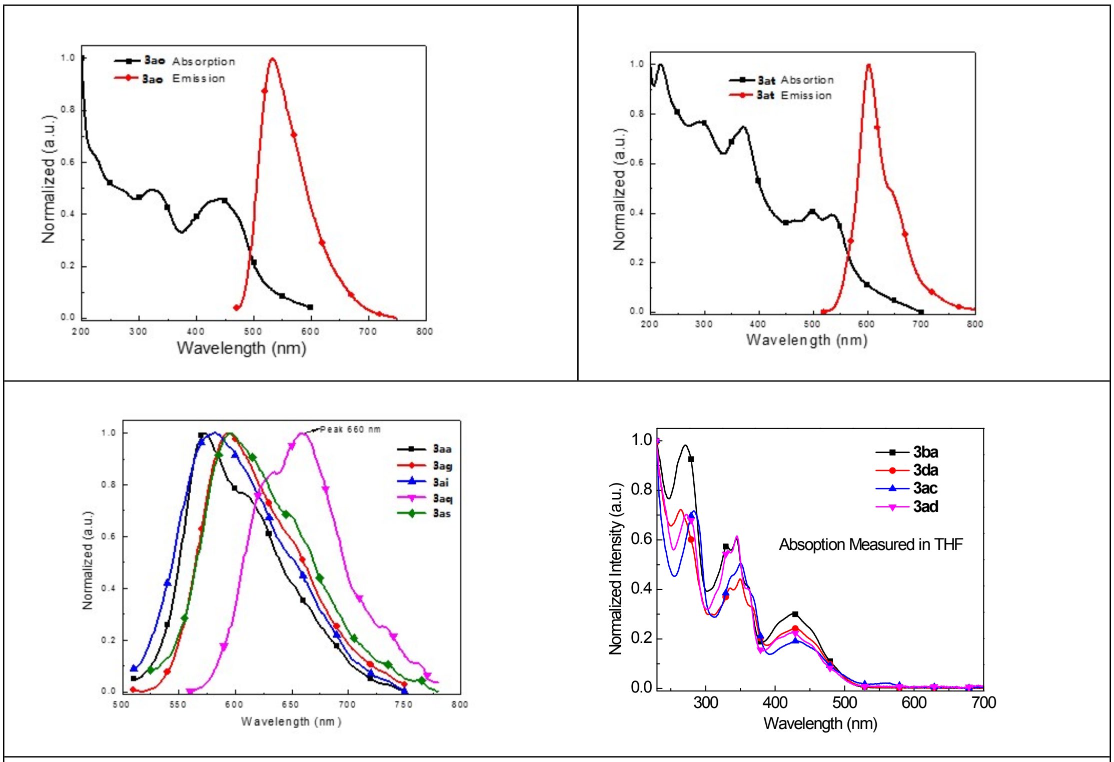

Figure S1. Absorption and emission spectra of selected compounds in solid state and solution.

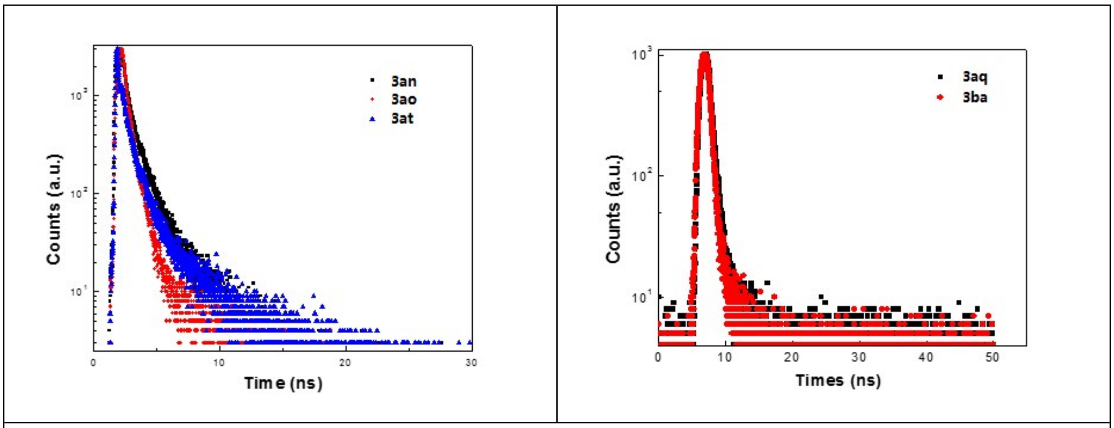

Figure S2. Transient decay curves of the selected compounds.

Characterization of the products

3-(Diphenylmethylene)-3H-benzo[f]chromene (3aa)

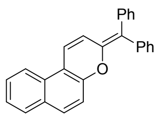

160.7 mg, 93% yield (PE/EA=100:1, R_f = 0.6), red solid: m.p. 108-110 °C. ^{1}H NMR (400 MHz, $CDCl_3$) δ 7.90 (d, *J* = 8.5 Hz, 1H), 7.72 (dd, *J* = 8.2, 1.3 Hz, 1H), 7.64 (d, *J* = 8.9 Hz, 1H), 7.51 – 7.43 (m, 3H), 7.41 – 7.25 (m, 8H), 7.23 – 7.16 (m, 1H), 7.11 – 7.05 (m, 1H), 7.02 (d, *J* = 10.3 Hz, 1H), 6.46 (d, *J* = 10.3 Hz, 1H). ^{13}C NMR (100 MHz, $CDCl_3$) δ 151.7, 146.9, 140.3, 139.4, 131.3, 130.03, 129.9, 129.7, 128.8, 128.6, 128.6, 127.7, 127.0, 126.9, 126.0, 124.3, 121.8, 121.1, 120.4, 116.8, 116.3, 114.3. HRMS (ESI+): m/z Calcd. for $C_{26}H_{19}O$ $[M+H]^+$: 347.1436, Found: 347.1421.

3-(diphenylmethylene)-9-methoxy-3H-benzo[f]chromene (3ab)

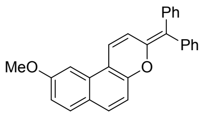

137.2 mg, 73% yield (PE/EA=20:1, R_f = 0.5), salmon pink solid: m.p. 128-129 °C. ^{1}H NMR (400 MHz, $CDCl_3$) δ 7.60 (d, *J* = 8.9 Hz, 1H), 7.55 (d, *J* = 8.8 Hz, 1H), 7.50 – 7.43 (m, 2H), 7.40 – 7.33 (m, 2H), 7.29 (tdd, *J* = 7.9, 5.2, 2.4 Hz, 6H), 7.23 – 7.15 (m, 2H), 7.01 (dd, *J* = 8.9, 2.4 Hz, 1H), 6.94 (t, *J* = 8.9 Hz, 2H), 6.44 (d, *J* = 10.3 Hz, 1H), 3.88 (s, 3H). ^{13}C NMR (100 MHz, $CDCl_3$) δ 158.7, 152.4, 146.8, 140.4, 139.4, 131.3, 130.2, 130.1, 129.8, 129.7, 128.5, 127.7, 126.8, 126.0, 125.2, 121.2, 120.5, 116.3, 116.1, 114.3, 113.4, 100.4, 55.2. HRMS (ESI+): m/z Calcd. for $C_{27}H_{21}O_2$ $[M+H]^+$: 377.1542, Found: 377.1526.

9-bromo-3-(diphenylmethylene)-3H-benzo[f]chromene (3ac)

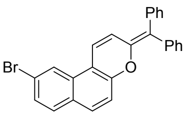

146.5 mg, 69% yield (PE/EA=100:1, R_f = 0.4), a salmon pink solid: m.p. 199-201 °C. ^{1}H NMR (400 MHz, $CDCl_3$) δ 8.04 (d, *J* = 1.9 Hz, 1H), 7.58 (t, *J* = 8.5 Hz, 2H), 7.49 – 7.42 (m, 2H), 7.42 – 7.34 (m, 2H), 7.34 – 7.25 (m, 5H), 7.23 – 7.16 (m, 1H), 7.07 (d, *J* = 8.9 Hz, 1H), 6.90 (d, *J* = 10.3 Hz, 1H), 6.48 (d, *J* = 10.3 Hz, 1H). ^{13}C NMR (100 MHz, $CDCl_3$) δ 152.4, 146.5, 140.1, 139.2, 131.2, 130.2, 130.0, 129.9, 129.7, 128.6, 128.3, 127.7, 127.7, 127.0, 126.2, 123.8, 117.0, 122.3, 121.6, 119.8, 117.2, 113.7. HRMS (ESI+): m/z Calcd. for $C_{26}H_{17}BrNaO$ $[M+Na]^+$: 447.0360, Found: 447.0334.

3-(diphenylmethylene)-5-methoxy-3H-benzo[f]chromene (3ad)

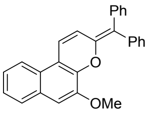

112.7 mg, 60% yield (PE/EA=20:1, R_f = 0.5), yellow solid: m.p. 153-155 ^oC. ^1H NMR (400 MHz, $CDCl_3$) δ 7.90 – 7.81 (m, 1H), 7.73 – 7.62 (m, 3H), 7.44 – 7.38 (m, 2H), 7.38 – 7.33 (m, 3H), 7.33 – 7.28 (m, 4H), 7.20 – 7.14 (m, 1H), 7.08 (s, 1H), 6.99 (d, J = 10.3 Hz, 1H), 6.40 (d, J = 10.3 Hz, 1H), 3.97 (s, 3H). ^{13}C NMR (100 MHz, $CDCl_3$) δ 147.3, 146.7, 140.1, 138.7, 131.6, 130.1, 129.5, 128.7, 128.2, 127.8, 127.6, 127.2, 127.0, 125.9, 124.9, 124.7, 124.1, 122.4, 121.0, 120.2, 115.0, 108.0, 56.0. HRMS (ESI+): m/z Calcd. for $C_{27}H_{20}NaO_2$ $[M+Na]^+$: 399.1361, Found: 399.1354.

5-bromo-3-(diphenylmethylene)-3H-benzo[f]chromene (3ae)

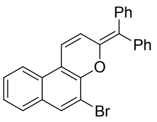

133.8 mg, 63% yield (PE/EA=100:1, R_f = 0.5), red solid: m.p. 172-174 ^oC. ^1H NMR (400 MHz, $CDCl_3$) δ 7.91 (s, 1H), 7.86 (d, J = 8.5 Hz, 1H), 7.67 – 7.54 (m, 3H), 7.53 – 7.44 (m, 1H), 7.42 – 7.35 (m, 3H), 7.35 – 7.26 (m, 6H), 7.25 – 7.16 (m, 1H), 6.95 (d, J = 10.3 Hz, 1H), 6.47 (d, J = 10.3 Hz, 1H). ^{13}C NMR (100 MHz, $CDCl_3$) δ 148.3, 146.4, 140.0, 138.4, 132.5, 131.3, 130.4, 130.2, 128.6, 127.8, 127.7, 127.6, 127.2, 127.1, 126.4, 125.2, 122.8, 121.3, 119.7, 118.0, 116.1, 110.4. HRMS (ESI+): m/z Calcd. for $C_{26}H_{17}BrNaO$ $[M+Na]^+$: 447.0360, Found: 447.0364. x-ray.

8-bromo-3-(diphenylmethylene)-3H-benzo[f]chromene (3af)

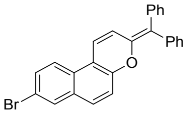

167.4 mg, 79% yield (PE/EA=100:1, R_f = 0.4), red solid: m.p. 152-153 ^oC. ^1H NMR (400 MHz, $CDCl_3$) δ 7.82 (d, J = 2.1 Hz, 1H), 7.69 (d, J = 9.0 Hz, 1H), 7.52 – 7.40 (m, 4H), 7.40 – 7.33 (m, 2H), 7.33 – 7.23 (m, 5H), 7.22 – 7.16 (m, 1H), 7.03 (d, J = 8.9 Hz, 1H), 6.88 (d, J = 10.3 Hz, 1H), 6.44 (d, J = 10.3 Hz, 1H). ^{13}C NMR (100 MHz, $CDCl_3$) δ 151.8, 146.5, 140.1, 139.2, 131.2, 130.9, 130.4, 130.1, 129.7, 128.9, 128.6, 127.7, 127.3, 127.0, 126.2, 122.9, 122.4, 119.8, 118.1, 117.8, 116.9, 114.6. HRMS (ESI+): m/z Calcd. for $C_{26}H_{17}BrNaO$ $[M+Na]^+$: 447.0360, Found: 447.0365.

3-(diphenylmethylene)-3H-benzo[f]chromene-8-carbonitrile (3ag)

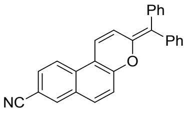

133.6 mg, 72% yield (PE/DCM/EA=100:20:1, R_f = 0.4), a red solid: m.p. 227-229 ^oC. ^1H NMR (400 MHz, $CDCl_3$) δ 8.05 (d, J = 1.7 Hz, 1H), 7.92 (d, J = 8.8 Hz, 1H), 7.65 (d, J = 8.9 Hz, 1H), 7.59 – 7.52 (m, 1H), 7.48 – 7.40 (m, 2H), 7.41 –

7.34 (m, 2H), 7.32 (t, J = 7.5 Hz, 3H), 7.29 – 7.19 (m, 4H), 7.12 (d, J = 8.9 Hz, 1H), 6.91 (d, J = 10.4 Hz, 1H), 6.50 (d, J = 10.3 Hz, 1H). ^{13}C NMR (100 MHz, $CDCl_3$) δ 153.9, 146.0, 139.7, 138.9, 134.5, 131.1, 130.4, 130.2, 129.7, 128.7, 128.6, 127.8, 127.5, 127.2, 126.5, 122.9, 122.5, 119.3, 119.1, 118.6, 118.0, 114.8, 107.6. HRMS (ESI+): m/z Calcd. for $C_{27}H_{17}NNaO$ $[M+Na]^+$: 394.1208, Found: 394.1202.

3-(diphenylmethylene)-3H-benzo[f]chromene-8-carbaldehyde (3ah)

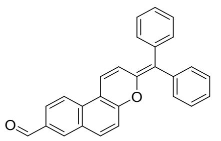

168.2 mg, 90% yield (PE/DCM/EA=100:10:1, R_f = 0.2), a red solid: m.p. 138-140°C. ^{1}H NMR (400 MHz, Chloroform-*d*) δ 10.07 (s, 1H), 8.18 (s, 1H), 8.01 – 7.88 (m, 2H), 7.78 (d, J = 8.9 Hz, 1H), 7.50 – 7.43 (m, 2H), 7.40 – 7.36 (m, 2H), 7.34 – 7.19 (m, 6H), 7.14 (d, J = 8.9 Hz, 1H), 6.99 (d, J = 10.3 Hz, 1H), 6.50 (d, J = 10.2 Hz, 1H). ^{13}C NMR (100 MHz, $CDCl_3$) δ 191.62, 154.19, 146.20, 139.86, 139.01, 134.80, 132.70, 131.99, 131.63, 131.12, 129.71, 129.02, 128.62, 127.77, 127.14, 126.41, 124.07, 122.61, 122.26, 119.77, 117.98, 114.98. HRMS (ESI+): m/z Calcd. for $C_{27}H_{19}O_2$ $[M+H]^+$: 375.1385, Found: 375.1374.

(3-(diphenylmethylene)-3H-benzo[f]chromen-8-yl)(phenyl)methanone (3ai)

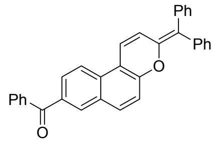

189.1 mg, 84% yield (PE/DCM/EA=100:10:1, R_f = 0.2), a red solid: m.p. 173-175 °C. ^{1}H NMR (400 MHz, $CDCl_3$) δ 8.15 (d, J = 1.6 Hz, 1H), 8.03 – 7.92 (m, 2H), 7.87 – 7.80 (m, 2H), 7.71 (d, J = 8.8 Hz, 1H), 7.64 – 7.57 (m, 1H), 7.52 – 7.44 (m, 4H), 7.42 – 7.35 (m, 2H), 7.34 – 7.26 (m, 5H), 7.24 – 7.17 (m, 1H), 7.12 (d, J = 8.9 Hz, 1H), 7.02 (d, J = 10.3 Hz, 1H), 6.51 (d, J = 10.3 Hz, 1H). ^{13}C NMR (100 MHz, $CDCl_3$) δ 196.6, 153.6, 146.4, 140.0, 139.1, 137.8, 133.2, 132.6, 132.3, 131.6, 131.2, 130.8, 129.9, 129.7, 128.6, 128.6, 128.3, 127.7, 127.1, 127.0, 126.3, 122.4, 121.6, 120.0, 117.7, 117.3, 114.6. HRMS (ESI+): m/z Calcd. for $C_{33}H_{23}O_2$ $[M+H]^+$: 451.1698, Found: 451.1690.

methyl 3-(diphenylmethylene)-3H-benzo[f]chromene-8-carboxylate (3aj)

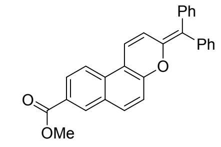

129.3 mg, 64% yield (PE/EA=20:1, R_f = 0.3), a orange solid: m.p.177-179 °C. ^{1}H NMR (400 MHz, $CDCl_3$) δ 8.46 (d, J = 1.7 Hz, 1H), 8.04 (dd, J = 8.9, 1.7 Hz, 1H), 7.91 (dd, J = 8.8, 2.4 Hz, 1H), 7.77 – 7.68 (m, 1H), 7.45 (dd, J = 8.3, 1.4 Hz, 2H), 7.40 – 7.34 (m, 2H), 7.34 – 7.25 (m, 5H), 7.25 – 7.18 (m, 1H), 7.14 – 7.09 (m, 1H), 7.05 – 6.95 (m, 1H), 6.48 (d, J = 10.3 Hz, 1H), 3.95 (s, 3H). ^{13}C NMR (100 MHz, $CDCl_3$) δ 167.0, 153.5, 146.4, 140.0, 139.1, 131.6, 131.4, 131.2, 131.1, 129.7, 128.9, 128.6, 127.8, 127.0, 126.4, 126.3, 125.9, 122.3, 121.4,

120.0, 117.7, 114.6, 52.2. HRMS (ESI+): m/z Calcd. for $C_{28}H_{21}O_3$ [M+H]$^+$: 405.1491, Found: 405.1479.

3-(diphenylmethylene)-N,N-diphenyl-3H-benzo[f]chromen-8-amine (3ak)

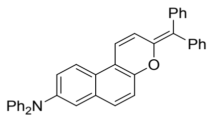

164.7 mg, 63% yield (PE/EA=50:1, R_f = 0.4), a red solid: m.p. 202-204 °C. ^{1}H NMR (400 MHz, $CDCl_3$) δ 7.74 (d, *J* = 9.1 Hz, 1H), 7.50 – 7.42 (m, 2H), 7.37 – 7.30 (m, 4H), 7.29 – 7.23 (m, 7H), 7.23 – 7.19 (m, 3H), 7.18 – 7.12 (m, 1H), 7.12 – 7.06 (m, 4H), 7.02 – 6.96 (m, 3H), 6.92 (d, *J* = 10.3 Hz, 1H), 6.44 (d, *J* = 10.2 Hz, 1H). ^{13}C NMR (100 MHz, $CDCl_3$) δ 150.9, 147.6, 146.8, 144.2, 140.3, 139.4, 131.3, 130.9, 129.6, 129.3, 128.9, 128.5, 127.7, 126.8, 126.0, 125.4, 125.1, 124.1, 122.8, 122.2, 121.9, 121.1, 120.4, 117.0, 116.1, 114.4. HRMS (ESI+): m/z Calcd. for $C_{38}H_{28}NO$ [M+H]$^+$: 514.2171, Found: 514.2150.

11-(diphenylmethylene)-2,2-diphenyl-2,11-dihydrobenzo[f]pyrano[3,2-h]chromene (3al)

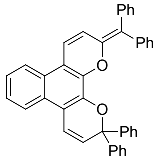

234.3 mg, 85% yield (PE/EA=100:1, R_f = 0.3), a red solid: m.p.197-199 °C. ^{1}H NMR (400 MHz, $CDCl_3$) δ 7.85 (d, *J* = 7.9 Hz, 1H), 7.80 – 7.72 (m, 3H), 7.53 – 7.45 (m, 4H), 7.42 – 7.36 (m, 2H), 7.35 – 7.17 (m, 12H), 7.12 – 7.02 (m, 3H), 6.91 (d, *J* = 10.3 Hz, 1H), 6.43 – 6.12 (m, 2H). ^{13}C NMR (100 MHz, $CDCl_3$) δ 146.7, 144.6, 142.5, 140.0, 139.6, 138.8, 131.5, 129.5, 128.8, 128.7, 128.1, 127.7, 127.5, 126.9, 126.8, 125.8, 125.1, 124.8, 124.2, 122.4, 121.9, 121.5, 120.1, 119.8, 116.6, 116.5, 116.0, 82.4. HRMS (ESI+): m/z Calcd. for $C_{41}H_{29}O_2$ [M+H]$^+$: 553.2168, Found: 553.2161. x-ray.

2-(diphenylmethylene)-2H-dibenzo[f,h]chromene (3am)

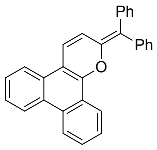

168.4 mg, 85% yield (PE/DCM=10:1, R_f = 0.5), a red solid: m.p. 209-211 °C. ^{1}H NMR (400 MHz, $CDCl_3$) δ 8.60 – 8.45 (m, 2H), 8.17 (dd, *J* = 8.2, 1.4 Hz, 1H), 7.94 (dd, *J* = 8.2, 1.5 Hz, 1H), 7.66 – 7.45 (m, 6H), 7.44 – 7.23 (m, 8H), 7.04 (d, *J* = 10.3 Hz, 1H), 6.58 (d, *J* = 10.3 Hz, 1H). ^{13}C NMR (100 MHz, $CDCl_3$) δ 148.0, 146.8, 140.4, 139.7, 131.2, 131.0, 129.7, 128.5, 127.9, 127.7, 127.7, 127.2, 127.0, 126.9, 126.8, 126.4, 124.9, 123.7, 123.0, 122.5, 122.2, 121.7, 121.6, 121.0, 116.4, 110.9. HRMS (ESI+): m/z Calcd. for $C_{30}H_{21}O$ [M+H]$^+$: 397.1592, Found: 397.1572.

10-(diphenylmethylene)-10H-phenanthro[4,5-fgh]chromene (3an)

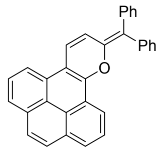

104.9 mg, 50% yield (PE/DCM=10:1, R_f = 0.5), a orange solid: m.p. 216-218 °C. ^{1}H NMR (400 MHz, $CDCl_3$) δ 8.13 (d, *J* = 9.2 Hz, 1H), 8.06 – 7.95 (m, 2H), 7.93 – 7.85 (m, 2H), 7.82 (d, *J* = 1.4 Hz, 2H), 7.66 (s, 1H), 7.64 – 7.59 (m, 2H), 7.49 – 7.37 (m, 5H), 7.37 – 7.30 (m, 4H), 6.70 (d, *J* = 10.0 Hz, 1H), 6.57 (d, *J* = 10.0 Hz, 1H). ^{13}C NMR (100 MHz, $CDCl_3$) δ 147.1, 146.7, 140.1, 139.5, 131.7, 131.5, 131.19, 129.9, 128.5, 127.9, 127.3, 127.1, 126.8, 126.6, 126.5, 126.3, 125.9, 125.7, 125.7, 124.9, 124.8, 124.5, 122.4, 121.8, 120.4, 118.9, 118.6, 117.9. HRMS (ESI+): m/z Calcd. for $C_{30}H_{21}O$ $[M+H]^+$: 397.1592, Found: 397.1572.

(7-(diphenylmethylene)pyrano[3,2-e]indol-3(7H)-yl)(phenyl)methanone (3ao)

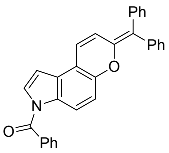

164.5 mg, 75% yield (PE/EA=20:1, R_f = 0.3), a orange solid: m.p. 223-225 °C. ^{1}H NMR (400 MHz, $CDCl_3$) δ 8.21 (d, *J* = 8.9 Hz, 1H), 7.75 – 7.68 (m, 2H), 7.64 – 7.57 (m, 1H), 7.56 – 7.46 (m, 4H), 7.43 – 7.34 (m, 2H), 7.34 – 7.24 (m, 6H), 7.23 – 7.15 (m, 1H), 6.92 (d, *J* = 8.9 Hz, 1H), 6.66 (d, *J* = 10.1 Hz, 1H), 6.61 (d, *J* = 3.8 Hz, 1H), 6.44 (d, *J* = 10.1 Hz, 1H). ^{13}C NMR (100 MHz, $CDCl_3$) δ 168.5, 150.1, 146.9, 140.4, 139.4, 134.3, 132.0, 131.7, 131.4, 129.7, 129.1, 128.8, 128.6, 128.5, 127.7, 127.0, 126.9, 126.1, 122.2, 121.2, 117.2, 117.0, 113.0, 112.9, 105.5. HRMS (ESI+): m/z Calcd. for $C_{31}H_{22}NO_2$ $[M+H]^+$: 440.1651, Found: 440.1638.

2-(diphenylmethylene)-5,7-dimethoxy-2H-chromene (3ap)

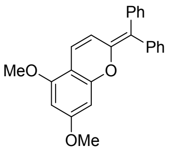

106.8 mg, 60% yield (PE/EA=20:1, R_f = 0.4), a orange solid: m.p. 136-137 °C. ^{1}H NMR (400 MHz, $CDCl_3$) δ 7.44 – 7.38 (m, 2H), 7.36 – 7.30 (m, 2H), 7.30 – 7.20 (m, 5H), 6.64 (d, *J* = 10.2 Hz, 1H), 6.15 (d, *J* = 10.2 Hz, 1H), 6.05 (q, *J* = 2.3 Hz, 2H), 3.77 (s, 3H), 3.76 (s, 3H). ^{13}C NMR (100 MHz, $CDCl_3$) δ 161.6, 155.6, 154.9, 146.9, 140.6, 139.7, 131.3, 129.7, 128.4, 127.7, 126.6, 125.8, 119.6, 117.3, 115.6, 104.8, 92.9, 92.8, 55.6, 55.5. HRMS (ESI+): m/z Calcd. for $C_{24}H_{21}O_3$ $[M+H]^+$: 357.1491, Found: 357.1483.

2,11-bis(diphenylmethylene)-2,11-dihydrobenzo[f]pyrano[3,2-h]chromene (3aq)

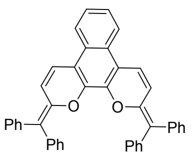

225.8 mg, 80% yield (PE/DCM/EA=100:10:1, R_f = 0.2), a purple solid: m.p. 223-225 °C. ^{1}H NMR (400 MHz, $CDCl_3$) δ 7.88 – 7.82 (m, 2H), 7.48 – 7.33 (m, 12H), 7.31 – 7.27 (m, 4H), 7.02 – 6.92 (m, 2H), 6.89 – 6.71 (m, 6H), 6.46 (d, *J* = 10.3

Hz, 2H). ^{13}C NMR (100 MHz, $CDCl_3$) δ 146.3, 140.8, 140.7, 138.1, 131.6, 129.5, 128.7, 127.3, 127.0, 126.4, 125.5, 124.9, 123.7, 121.7, 119.3, 118.6, 116.8. HRMS (ESI+): m/z Calcd. for $C_{42}H_{29}O_2$ $[M+H]^+$: 565.2168, Found: 565.2155.

(S)-3,3'-bis(diphenylmethylene)-3H,3'H-[6,6'-bibenzo[f]chromene]-5,5'-diol (3ar)

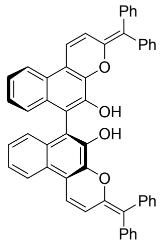

209.4 mg, 58% yield (PE/EA=20:1, R_f = 0.2), a red solid: m.p. 181-183 °C. ^{1}H NMR (400 MHz, Chloroform-*d*) δ 7.97 (d, *J* = 8.5 Hz, 2H), 7.44 – 7.34 (m, 10H), 7.33 – 7.29 (m, 6H), 7.27 – 7.20 (m, 4H), 7.21 – 7.15 (m, 6H), 7.14 – 7.08 (m, 2H), 6.66 (d, *J* = 10.3 Hz, 2H), 5.59 (s, 2H). ^{13}C NMR (100 MHz, $CDCl_3$) δ 145.4, 141.1, 140.8, 139.5, 139.3, 130.9, 129.5, 129.3, 128.5, 128.2, 127.2, 126.7, 125.4, 125.4, 124.9, 124.0, 121.8, 121.4, 120.6, 118.0, 116.1, 115.1. HRMS (ESI+): m/z Calcd. for $C_{52}H_{35}O_4$$[M+H]^+$: 723.2535, Found: 723.2531.

3,10-bis(diphenylmethylene)-3H,10H-chromeno[5,6-f]chromene (3as)

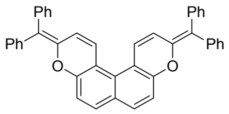

205.7 mg, 73% yield (PE/DCM=8:1, R_f = 0.3), a red solid: m.p. 224-226 °C. ^{1}H NMR (400 MHz, $CDCl_3$) δ 7.55 – 7.41 (m, 3H), 7.39 – 7.23 (m, 7H), 7.23 – 7.15 (m, 1H), 7.02 (d, *J* = 10.4 Hz, 1H), 6.93 (d, *J* = 8.7 Hz, 1H), 6.29 (d, *J* = 10.3 Hz, 1H). ^{13}C NMR (100 MHz, $CDCl_3$) δ 154.3, 146.1, 140.2, 139.2, 131.2, 131.0, 129.8, 128.5, 127.7, 127.1, 126.9, 126.1, 124.0, 119.8, 116.6, 115.3, 114.6. HRMS (ESI+): m/z Calcd. for $C_{42}H_{29}O_2$ $[M+H]^+$: 565.2168, Found: 565.2145.

2,8-bis(diphenylmethylene)-2,8-dihydrochromeno[6,5-f]chromene (3at)

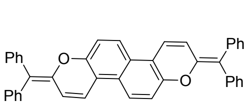

197.4 mg, 70% yield (PE/DCM=8:1, R_f = 0.3), a red solid: m.p. 309-311 °C. ^{1}H NMR (400 MHz, $CDCl_3$) δ 7.78 (d, *J* = 9.2 Hz, 1H), 7.47 (d, *J* = 7.7 Hz, 2H), 7.42 – 7.35 (m, 2H), 7.35 – 7.26 (m, 6H), 7.24 – 7.17 (m, 1H), 7.11 (d, *J* = 9.1 Hz, 1H), 6.98 (d, *J* = 10.3 Hz, 1H), 6.49 (d, *J* = 10.3 Hz, 1H). ^{13}C NMR (100 MHz, $CDCl_3$) δ 150.8, 146.7, 140.2, 139.3, 131.3, 129.7, 128.6, 127.7, 127.0, 126.1, 125.2, 123.0, 122.5, 120.2, 117.6, 116.7, 115.1. HRMS (ESI+): m/z Calcd. for $C_{42}H_{29}O_2$ $[M+H]^+$: 565.2168, Found: 565.2163.

1,2-bis(3-(diphenylmethylene)-3H-benzo[f]chromen-8-yl)disulfane (3au)

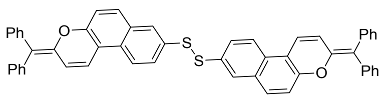

286.4 mg, 76% yield (PE/DCM /EA=100:10:1, R_f = 0.3), a red solid: m.p. 145-147 °C. ^{1}H NMR (400 MHz, $CDCl_3$) δ 7.90 – 7.78 (m, 4H), 7.59 (dd, *J* = 8.8, 2.0 Hz, 2H), 7.51 (d, *J* = 8.9 Hz, 2H), 7.47 – 7.42 (m, 4H), 7.40 – 7.33 (m, 4H), 7.33 – 7.23 (m, 10H), 7.21 – 7.15 (m, 2H), 7.04 (d, *J* = 8.9 Hz, 2H), 6.92 (d, *J* = 10.3 Hz, 2H), 6.45 (d, *J* = 10.3 Hz, 2H). ^{13}C NMR (100 MHz, $CDCl_3$) δ 152.1, 146.6, 140.1, 139.2, 132.6, 131.2, 129.9, 129.7, 129.5, 128.6, 128.0, 128.0, 127.7, 127.3, 127.0, 126.2, 122.4, 122.3, 120.0, 117.6, 116.8, 114.6. HRMS (ESI+): m/z Calcd. for $C_{52}H_{35}O_2S_2$ [M+H]$^+$: 755.2078, Found: 755.2076.

3-(bis(4-fluorophenyl)methylene)-3H-benzo[f]chromene (3ba)

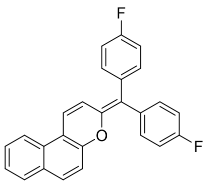

127.8 mg, 67% yield (PE/EA=100:1, R_f = 0.6), a red solid: m.p. 111-113 °C. ^{1}H NMR (400 MHz, $CDCl_3$) δ 7.87 (d, *J* = 8.5 Hz, 1H), 7.73 – 7.66 (m, 1H), 7.61 (d, *J* = 8.9 Hz, 1H), 7.50 – 7.29 (m, 4H), 7.22 – 7.15 (m, 2H), 7.08 – 6.93 (m, 6H), 6.36 (d, *J* = 10.3 Hz, 1H). ^{13}C NMR (100 MHz, $CDCl_3$) δ 162.7 (d, *J* = 64 Hz), 160.2 (d, *J* = 64 Hz), 151.6, 146.8, 136.0 (d, *J* = 12 Hz), 135.3 (d, *J* = 12 Hz), 132.8, 132.7, 131.2, 131.1, 130.2, 129.9, 128.8, 128.6, 127.1, 124.4, 121.2, 121.1, 120.7, 116.6, 115.6 (d, *J* = 84 Hz), 114.6 (d, *J* = 84 Hz), 114.2, 114.0. ^{19}F NMR (377 MHz, $CDCl_3$) δ -115.04, -115.69. HRMS (ESI+): m/z Calcd. for $C_{26}H_{17}F_2O$ [M+H]$^+$: 383.1247, Found: 383.1241.

3-(bis(4-chlorophenyl)methylene)-3H-benzo[f]chromene (3ca)

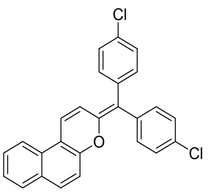

168.1 mg, 81% yield (PE/EA=100:1, R_f = 0.4), a red solid: m.p. 117-119 °C. ^{1}H NMR (400 MHz, $CDCl_3$) δ 7.87 (d, *J* = 8.5 Hz, 1H), 7.69 (d, *J* = 8.1 Hz, 1H), 7.62 (d, *J* = 8.9 Hz, 1H), 7.49 – 7.42 (m, 1H), 7.37 – 7.29 (m, 5H), 7.27 – 7.21 (m, 2H), 7.17 – 7.09 (m, 2H), 7.06 – 6.96 (m, 2H), 6.37 (d, *J* = 10.2 Hz, 1H). ^{13}C NMR (100 MHz, $CDCl_3$) δ 151.5, 147.3, 138.3, 137.5, 132.8, 132.5, 131.5, 130.8, 130.4, 129.9, 128.9, 128.8, 128.6, 127.9, 127.1, 124.5, 121.3, 121.1, 120.9, 116.5, 114.1, 113.7. HRMS (ESI+): m/z Calcd. for $C_{26}H_{17}Cl_2O$ [M+H]$^+$: 415.0656, Found: 415.0653.

3-(bis(4-bromophenyl)methylene)-3H-benzo[f]chromene (3da)

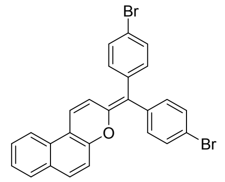

211.7 mg, 84% yield (PE/EA=100:1, R_f = 0.6), an orange solid: m.p. 155-157 °C. ^{1}H NMR (400 MHz, $CDCl_3$) δ 7.83 (d, *J* = 8.4 Hz, 1H), 7.67 (d, *J* = 8.1 Hz, 1H), 7.59 (d, *J* = 8.3 Hz, 1H), 7.51 – 7.14 (m, 9H), 7.09 – 6.89 (m, 4H), 6.34 (d, *J* = 10.3 Hz, 1H). ^{13}C NMR (100 MHz, $CDCl_3$) δ 151.4, 147.3, 138.7, 137.9, 132.9, 131.9, 131.2, 130.9, 130.4, 129.9, 128.7, 128.6, 127.2, 124.6, 121.4, 121.1, 121.0, 120.8, 119.8, 116.4, 114.1, 113.6. HRMS (ESI+): m/z Calcd. for $C_{26}H_{17}Br_2O$ $[M+H]^+$: 504.9626, Found: 504.9616.

3-(phenyl(p-tolyl)methylene)-3H-benzo[f]chromene (3ea)

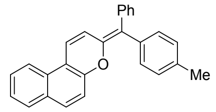

144.1 mg, 80% yield (Z/E = 1:1, PE/EA=100:1, R_f = 0.5), a red solid: m.p. 96-98 °C. ^{1}H NMR (400 MHz, $CDCl_3$) δ 7.95 – 7.80 (m, 1H), 7.71 – 7.63 (m, 1H), 7.65 – 7.59 (m, 1H), 7.50 – 7.41 (m, 2H), 7.40 – 7.26 (m, 6H), 7.19 – 7.14 (m, 2H), 7.14 – 7.10 (m, 1H), 7.09 – 7.03 (m, 1H), 7.00 – 6.90 (m, 1H), 6.50 – 6.40 (m, 1H), 2.37 (s, 2H), 2.34 (s, 1H). ^{13}C NMR (100 MHz, $CDCl_3$) δ 151.7, 146.7, 146.5, 140.4, 139.5, 137.3, 136.5, 135.7, 131.3, 131.1, 129., 129.81, 129.6, 129.5, 129.3, 128.8, 128.5, 128.5, 128.4, 127.7, 126.9, 126.8, 126.0, 124.2, 121.9, 122.0, 121.1, 120.1, 120.0, 116.7, 116.2, 116.1, 114.3, 21.2. HRMS (ESI+): m/z Calcd. for $C_{27}H_{21}O$ $[M+H]^+$: 361.1592, Found: 361.1591.

3-((4-chlorophenyl)(phenyl)methylene)-3H-benzo[f]chromene (3fa)

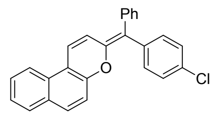

157.7 mg, 83% yield (Z/E = 1:1, PE/EA=100:1, R_f = 0.6), a red solid: m.p. 119-121 °C. ^{1}H NMR (400 MHz, $CDCl_3$) δ 7.94 – 7.82 (m, 1H), 7.74 – 7.66 (m, 1H), 7.66 – 7.58 (m, 1H), 7.50 – 7.41 (m, 2H), 7.40 – 7.28 (m, 5H), 7.27 – 7.16 (m, 4H), 7.08 – 6.97 (m, 2H), 6.48 – 6.37 (m, 1H). ^{13}C NMR (100 MHz, $CDCl_3$) δ 151.5, 147.2, 139.8, 137.9, 132.6, 131.3, 131.2, 130.9, 130.2, 129.9, 129.6, 128.8, 128.7, 128.6, 127.8, 127.9, 127.1, 126.2, 124.4, 121.5, 121.2, 121.1, 120.9, 120.7, 116.7, 116.5, 115.0, 114.2. HRMS (ESI+): m/z Calcd. for $C_{26}H_{18}ClO$ $[M+H]^+$: 381.1046, Found: 381.1045.

(Z)-3-(naphthalen-2-yl(phenyl)methylene)-3H-benzo[f]chromene (3ga)

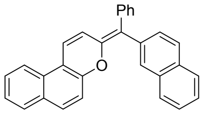

142.6 mg, 72% yield (Z/E = 1:1, PE/EA=100:1, R_f = 0.5), a red solid: m.p. 83-85 °C. ^{1}H NMR (400 MHz, $CDCl_3$) δ 7.91 – 7.74 (m, 5H), 7.73 – 7.63 (m, 2H), 7.63 – 7.54 (m, 1H), 7.52 – 7.24 (m, 10H), 7.23 – 6.88 (m, 3H), 6.55 – 6.44 (m, 1H). ^{13}C NMR (100 MHz, $CDCl_3$) δ 151.7, 147.2, 140.3, 139.3, 137.7, 137.1, 133.5, 133.3, 132.4, 131.9, 131.3, 130.1, 129.8, 129.6, 128.7, 128.6, 128.5, 128.2, 128.1, 128.0, 127.7, 127.6, 127.4, 127.0, 126.9, 126.8, 126.1, 126.1, 125.8, 125.7, 125.5, 124.3, 121.7, 121.1, 120.6, 120.6, 116.7, 116.6, 116.2, 116.0, 114.3, 114.3. HRMS (ESI+): m/z Calcd. for $C_{30}H_{21}O$ $[M+H]^+$: 397.1592, Found: 397.1587.

Investigation of Intermediates

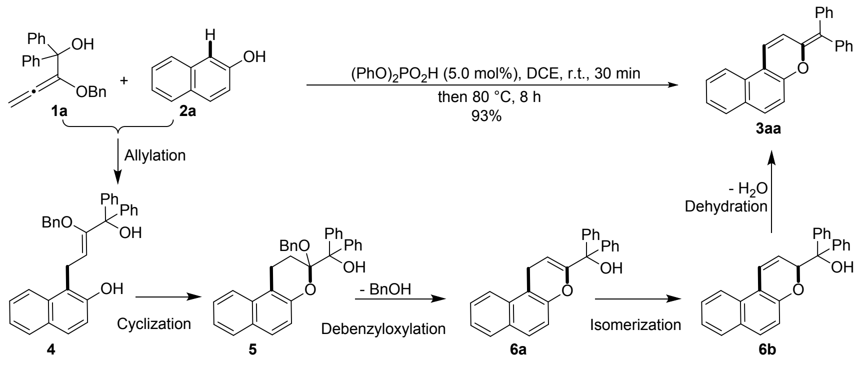

Proposed Reaction Sequence

(Z)-1-(3-(benzyloxy)-4-hydroxy-4,4-diphenylbut-2-en-1-yl)naphthalen-2-ol (4)

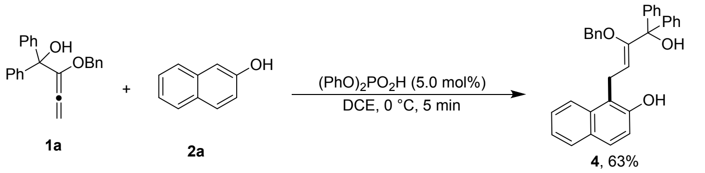

The reaction mixture of **1a** (49.2 mg, 0.15 mmol) and **2a** (14.4 mg, 0.1 mmol) in DCE solvent (0.5 mL) was cooled to 0 °C, catalyst phosphoric acid (1.3 mg, 5.0 mol%) was added and stirred for 5 min. After the completion of the reaction determined by TLC. The reaction mixture was purified by flash column chromatography on silica gel directly to afford product **4** in 63% yield (29.7 mg) as a slurry liquid. ^{1}H NMR (400 MHz, Chloroform-*d*) δ 7.95 – 7.83 (m, 2H), 7.77 (d, *J* = 8.1 Hz, 1H), 7.63 (d, *J* = 8.8 Hz, 1H), 7.49 – 7.44 (m, 5H), 7.38 – 7.30 (m, 7H), 7.16 – 7.11 (m, 3H), 7.08 (d, *J* = 8.8 Hz, 1H), 6.86 – 6.64 (m, 2H), 4.91 (t, *J* = 8.0 Hz, 1H), 4.69 (d, *J* = 2.8 Hz, 0.6H), 4.59 (s, 1H), 4.04 (s, 0.7H), 3.75 (d, *J* = 8.0 Hz, 2H). ^{13}C NMR (100 MHz, $CDCl_3$) δ 156.24, 152.32, 144.71, 136.84, 132.85, 129.26, 128.72, 128.57, 128.07, 127.73, 127.61, 127.25, 126.99, 126.46, 126.30, 122.66, 122.45, 118.60, 117.70, 101.08, 83.45, 69.30, 22.19. HRMS (ESI+): m/z Calcd. for $C_{33}H_{28}NaO_3$ $[M+Na]^+$: 495.1936, Found: 495.1931.

(3-(benzyloxy)-2,3-dihydro-1H-benzo[f]chromen-3-yl)diphenylmethanol (5)

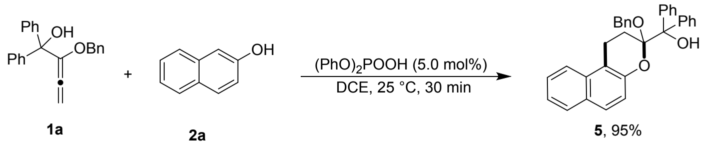

Method 1: The reaction mixture of **1a** (32.8 mg, 0.10 mmol) and **2a** (21.6 mg, 0.15 mmol) in DCE solvent (1.0 mL) was added catalyst phosphoric acid (1.3 mg, 5.0 mol%) and stirred for 30 min. After the completion of the reaction determined by TLC. The organic solvent was removed *in vacuo*. The residue

was purified by flash column chromatography on silica gel to afford product **5** in 95% yield (44.8 mg) as a slurry liquid.

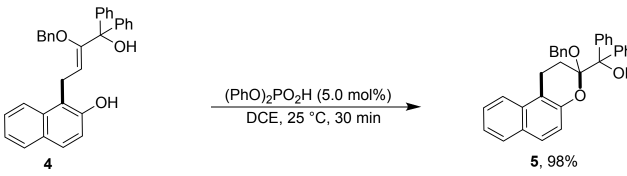

Method 2: To the solution of **4** (23.6 mg, 0.05 mmol) in DCE solvent (0.5 mL) was added catalyst phosphoric acid (0.7 mg, 5.0 mol%) and stirred for 30 min. After the completion of the reaction determined by TLC. The organic solvent was removed *in vacuo*. The residue was purified by flash column chromatography on silica gel to afford product **5** in 98% yield (23.1 mg) as a slurry liquid. ^{1}H NMR (400 MHz, Chloroform-*d*) δ 8.01 (d, J = 7.8 Hz, 2H), 7.91 – 7.66 (m, 5H), 7.51 – 7.43 (m, 1H), 7.39 – 7.32 (m, 4H), 7.29 – 7.21 (m, 3H), 7.21 – 7.13 (m, 1H), 7.09 – 7.00 (m, 3H), 6.81 – 6.69 (m, 2H), 4.01 (dd, J = 62.8, 11.1 Hz, 2H), 3.81 (s, 1H), 3.12 – 2.93 (m, 2H), 2.55 – 2.39 (m, 1H), 2.09 – 1.94 (m, 1H). ^{13}C NMR (100 MHz, $CDCl_3$) δ 149.0, 144.0, 143.4, 137.6, 132.5, 129.5, 128.4, 128.1, 128.0, 127.9, 127.8, 127.7, 127.6, 127.3, 127.1, 127.0, 126.9, 126.5, 123.9, 122.2, 118.2, 115.8, 102.9, 80.3, 66.8, 26.6, 17.9. HRMS (ESI+): m/z Calcd. for $C_{33}H_{28}NaO_3$ $[M+Na]^+$: 495.1936, Found: 495.1932.

(1H-benzo[f]chromen-3-yl)diphenylmethanol (6a), (1H-benzo[f]chromen-3-yl)diphenylmethanol (6b)

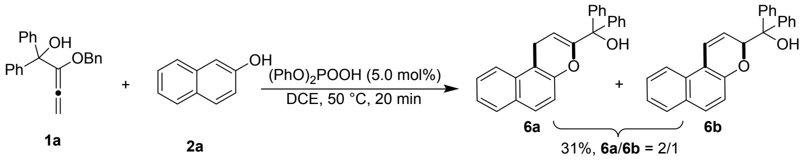

The reaction mixture of **1a** (32.8 mg, 0.10 mmol) and **2a** (21.6 mg, 0.15 mmol) in DCE solvent (1.0 mL) was added catalyst phosphoric acid (1.3 mg, 5.0 mol%) and stirred at 50 °C for 20 min. The organic solvent was removed *in vacuo*. The residue was purified by flash column chromatography on silica gel to afford a mixture of **6a** and **6b** in total 31% yield (11.3 mg, **6a/6b** = 2:1). ^{1}H NMR (400 MHz, Chloroform-*d*) δ 7.80 (d, J = 8.2 Hz, 1H), 7.68 (dd, J = 17.6, 8.6 Hz, 2H), 7.53 (t, J = 7.6 Hz, 1H), 7.48 – 7.44 (m, 4.6H), 7.40 – 7.28 (m, 10.4H), 7.04 (d, J = 8.9 Hz, 1H), 6.77 – 6.65 (m, 0.5H), 6.58 – 6.48 (m, 0.5H), 5.78 – 5.70 (m, 0.5H), 5.05 (s, 1H), 4.86 (t, J = 3.6 Hz, 1H), 3.74 (d, J = 3.6 Hz, 2H), 3.43 (s, 1H). ^{13}C NMR (100 MHz, $CDCl_3$) δ 143.84, 130.49, 128.49, 128.27, 128.09, 127.92, 127.84, 127.59, 126.66, 124.29, 122.48, 120.14, 117.77, 100.94, 21.82. HRMS (ESI+): m/z Calcd. for $C_{33}H_{29}O_3$ $[M+H]^+$:

473.2117, Found: 473.2110.

1-(1H-benzo[f]chromen-3-yl)cyclohexan-1-ol (7)

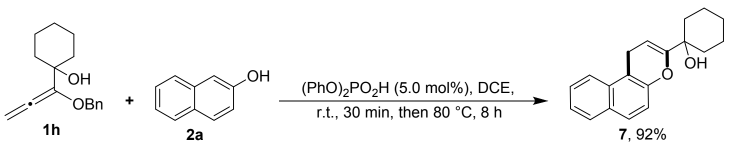

To the solution of allene **1h** (0.75 mmol, 1.5 equiv.) and naphthol **2a** (0.5 mmol) in DCE solvent (5.0 mL) was added phosphoric acid (10 mol%). After stirring at 25 °C for 30 min, the reaction mixture was heated to 70 °C and the stirring continued for another 8 h. Then the organic solvent was removed *in vacuo*. the residue was purified by flash column chromatography to afford the desired product **7** in a 92% yield (128.7 mg) as a white solid. ^{1}H NMR (400 MHz, $CDCl_3$) δ 7.79 (d, *J* = 8.1 Hz, 1H), 7.75 – 7.62 (m, 2H), 7.56 – 7.48 (m, 1H), 7.44 – 7.37 (m, 1H), 7.12 (d, *J* = 8.9 Hz, 1H), 5.25 (t, *J* = 3.6 Hz, 1H), 3.72 (d, *J* = 3.5 Hz, 2H), 1.98 – 1.83 (m, 3H), 1.81 – 1.52 (m, 7H). ^{13}C NMR (100 MHz, $CDCl_3$) δ 148.9, 130.2, 128.2, 128.0, 126.5, 124.0, 122.5, 117.8, 111.3, 93.8, 71.8, 34.9, 25.6, 21.7, 21.5. HRMS (ESI+): m/z Calcd. for $C_{19}H_{21}O_2$ $[M+H]^+$: 281.1542, Found: 281.1544.

3-(1-methoxycyclohexyl)-1H-benzo[f]chromene (7')

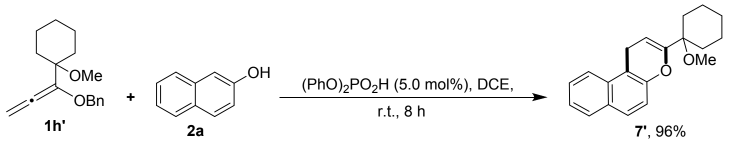

To the solution of allene **1h'** (0.75 mmol, 1.5 equiv.) and naphthol **2a** (0.5 mmol) in DCE solvent (5.0 mL) was added phosphoric acid (10 mol%). After stirring at 25 °C for 8 h. Then the organic solvent was removed *in vacuo*. The residue was purified by flash column chromatography to afford the desired product **7**' in a 96% yield (141.2 mg) as a white solid. ^{1}H NMR (400 MHz, $CDCl_3$) δ 7.83 – 7.71 (m, 1H), 7.72 – 7.61 (m, 2H), 7.57 – 7.46 (m, 1H), 7.45 – 7.35 (m, 1H), 7.22 – 7.10 (m, 1H), 5.15 (q, *J* = 3.1 Hz, 1H), 3.73 (t, *J* = 2.9 Hz, 2H), 3.19 (d, *J* = 2.3 Hz, 3H), 2.04 – 1.89 (m, 2H), 1.79 – 1.48 (m, 7H), 1.41 – 1.21 (m, 1H); ^{13}C NMR (101 MHz, $CDCl_3$) δ 151.37, 149.13, 131.90, 130.21, 128.16, 127.83, 126.41, 123.93, 122.31, 118.09, 111.03, 97.57, 77.32, 49.67, 32.01, 25.78, 21.63, 21.56. HRMS (ESI+): m/z Calcd. for $C_{20}H_{23}O_2$ $[M+H]^+$: 295.1698, Found: 2 295.1695.

Conversion from compound 5 to 3aa

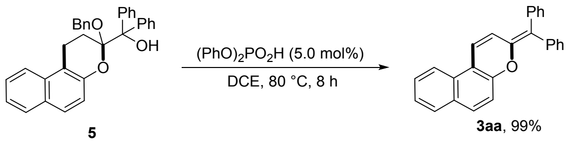

To the solution of **5** (47.2 mg, 0.1 mmol) in DCE solvent (1.0 mL) was added catalyst phosphoric acid (1.3 mg, 5.0 mol%) and stirred at 80 °C for 8 h. After the completion of the reaction determined by TLC. The organic solvent was removed *in vacuo*. The residue was purified by flash column chromatography on silica gel to afford product **3aa** in 99% yield (34.4 mg).

X-Ray crystallographic determination of compounds 3ae and 3al

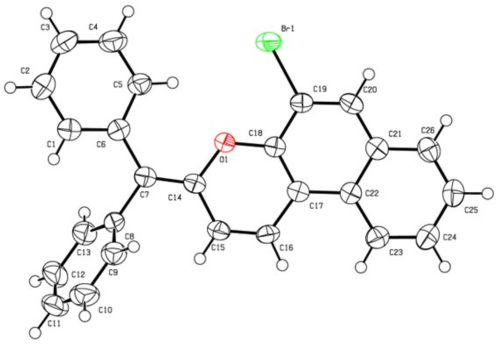

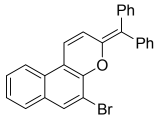

(CCDC: 1861980)

Bond precision	C-C = 0.0038 Å		Wavelength = 0.71073
Cell	a = 12.8537 (19)	b = 13.3132 (18)	c = 11.8696 (17)
	alpha = 90	beta = 114.474 (2)	gamma = 90

Temperature: 296 K

	Calculated	Reported
Volume/ Å^3	1848.7 (5)	1848.7 (5)
Space group	P21/c	P21/c
Hall group	-P2ybc	-P2ybc
Moiety formula	$C_{26}H_{17}BrO$	
Sum formula	$C_{26}H_{17}BrO$	$C_{26}H_{17}BrO$
Mr	425.30	425.30
Dx, g/cm^3	1.528	1.528
Z	4	4
μ/mm^{-1}	2.238	2.238
F0000	864.0	864.0
F0000’	863.17	
h, k, lmax	16, 17, 15	16, 17, 15
Nref	4205	4205
Tmin, Tmax	0.516, 0.511	0.347, 0.746
Tmin’	0.506	

Data completeness = 0.996 Theta (max) = 27.421

R (reflections) = 0.0395 (3139) Wr2 (reflections) = 0.0986 (4190)

S = 1.000 Npar = 254

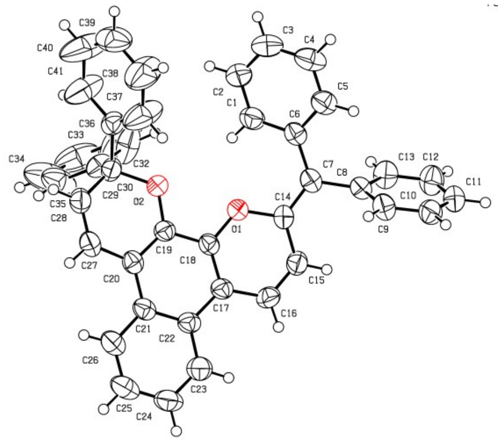

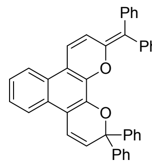

(CCDC: 1861979)

Bond precision	C-C = 0.0057 Å		Wavelength = 0.71073
Cell	a = 9.7427 (14)	b = 13.5639 (19)	c = 21.658 (3)
	alpha = 90	beta = 90	gamma = 90
Temperature: 296 K			
	Calculated		Reported
Volume/ $Å^3$	2862.1 (7)		2862.0 (7)
Space group	Pna21		Pna21
Hall group	P2c-2n		P2c-2n
Moiety formula	$C_{41}H_{28}O_2$		
Sum formula	$C_{41}H_{28}O_2$		$C_{41}H_{28}O_2$
Mr	552.63		552.63
Dx, g/cm^3	1.283		1.283
Z	4		4
μ/mm^{-1}	0.077		0.077
F0000	1160.0		1160.0
F0000'	1160.48		
h, k, lmax	11, 16, 25		11, 16, 25
Nref	5012 [2579]		4566
Tmin, Tmax	0.973, 0.977		0.535, 0.745
Tmin'	0.970		

Data completeness = 1.77 / 0.91 Theta (max) = 24.947

R (reflections) = 0.0419 (3774) Wr2 (reflections) = 0.1106 (4566)

S = 0.999 Npar = 377

NMR spectra

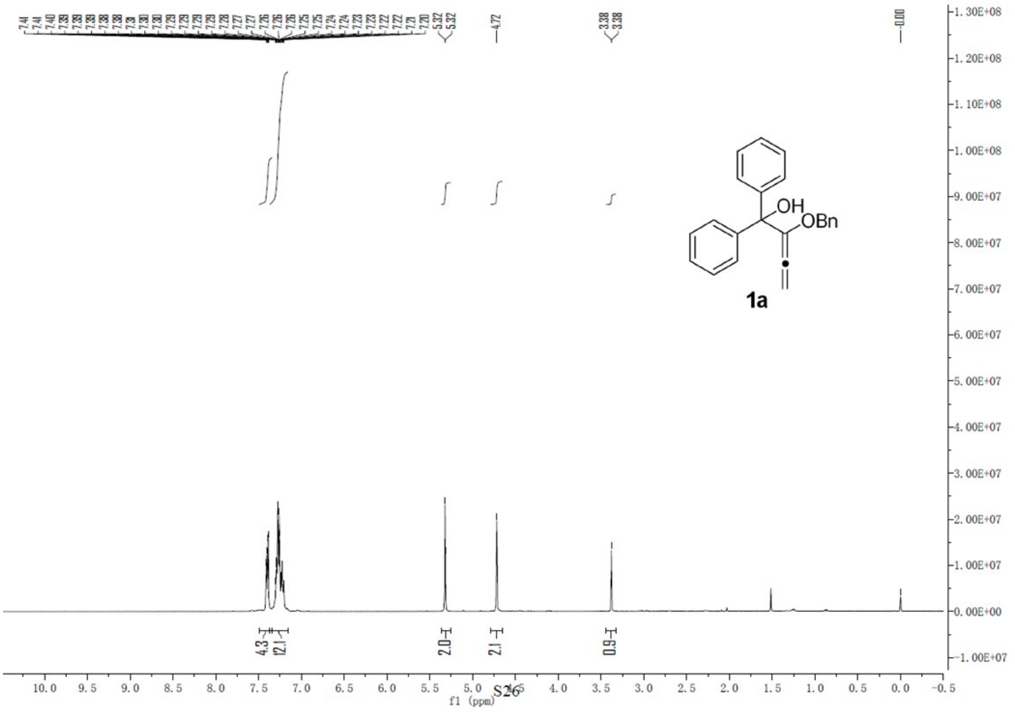

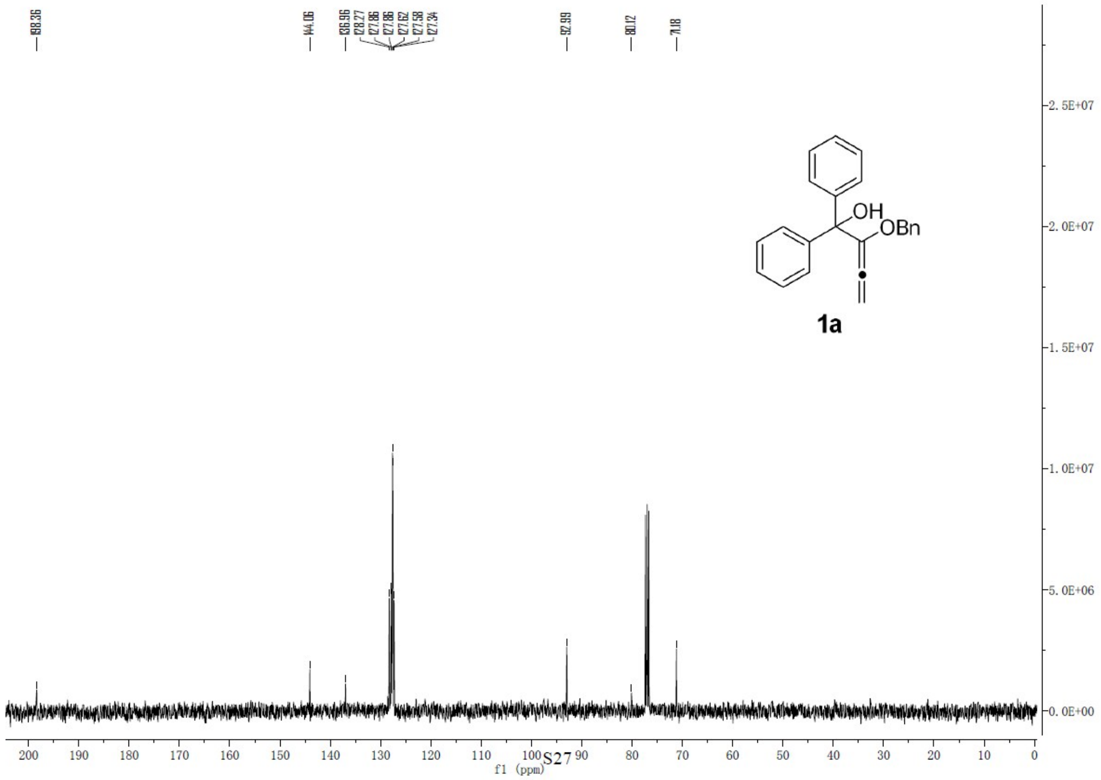

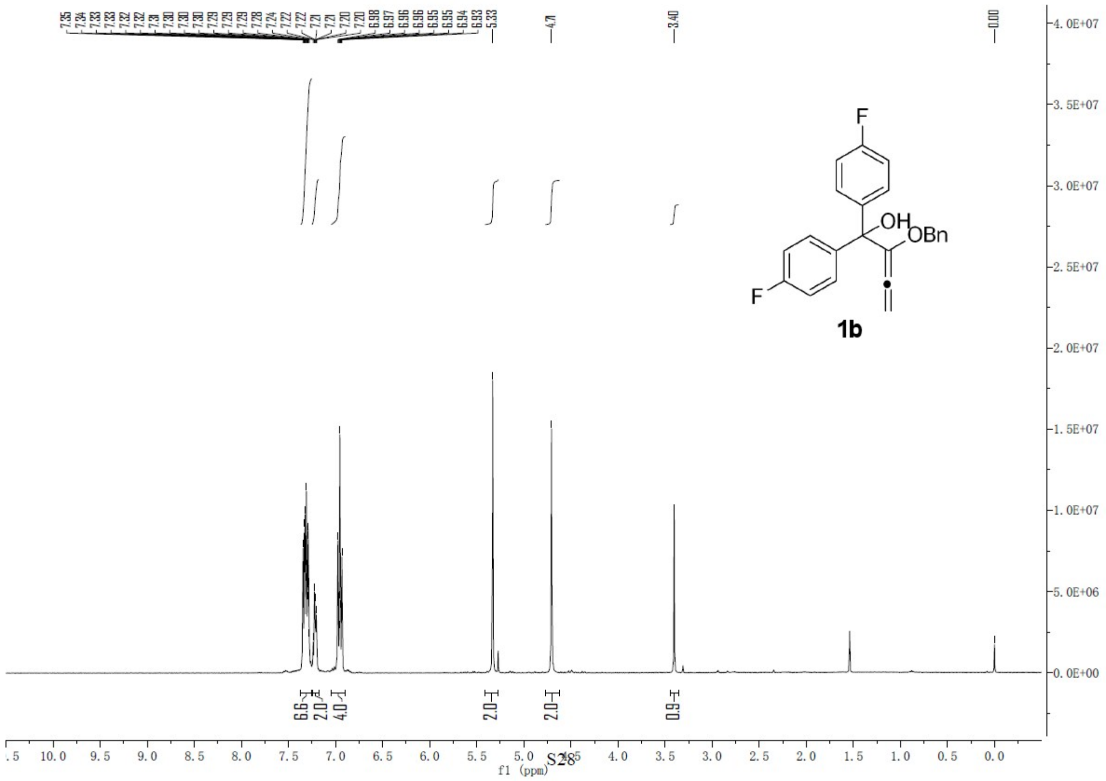
1b

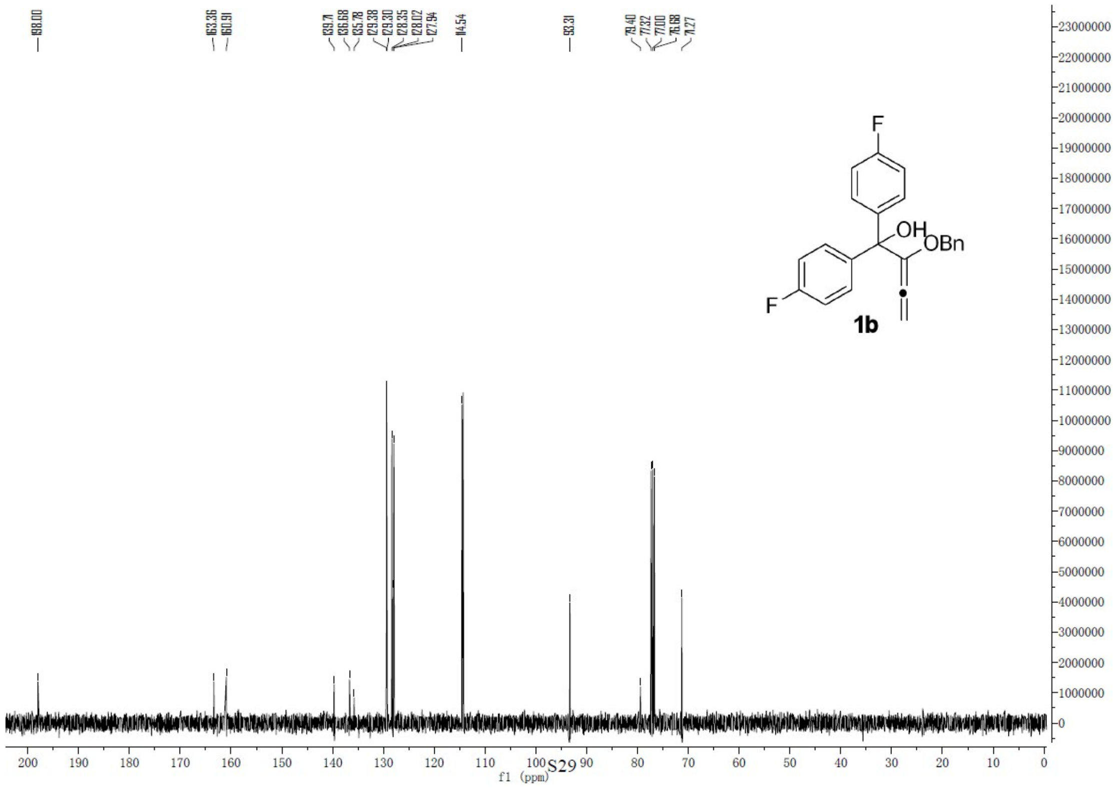
1b

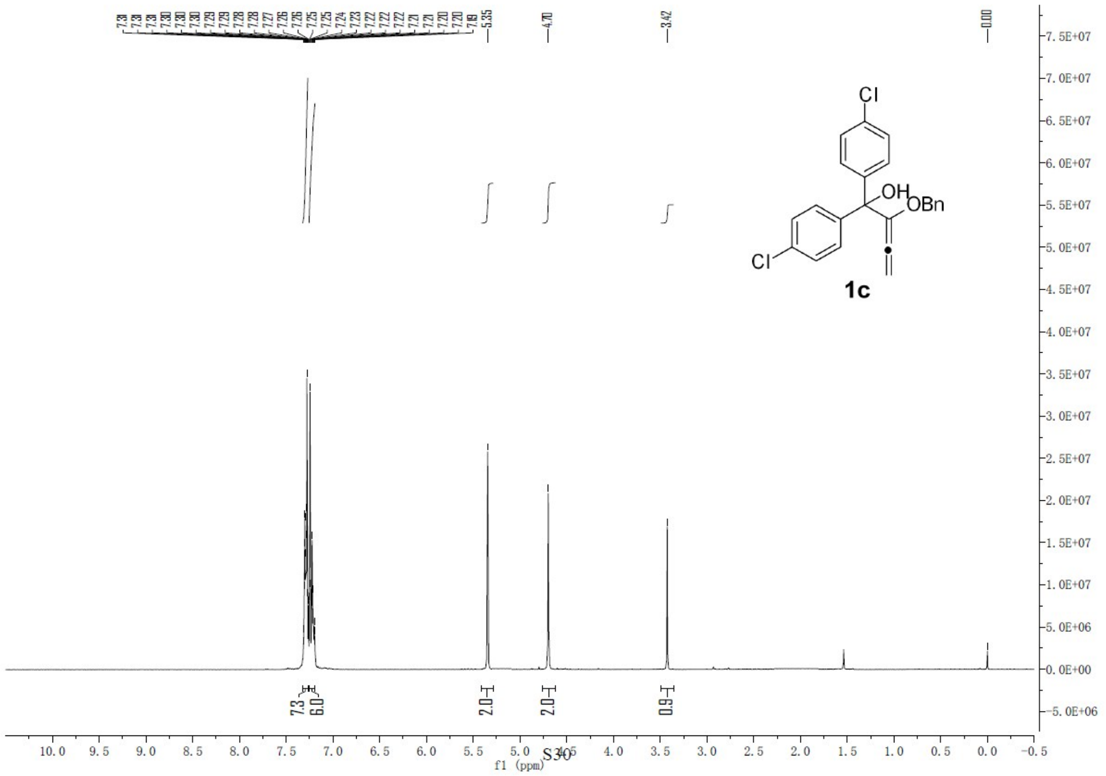
1c
f1 (ppm)

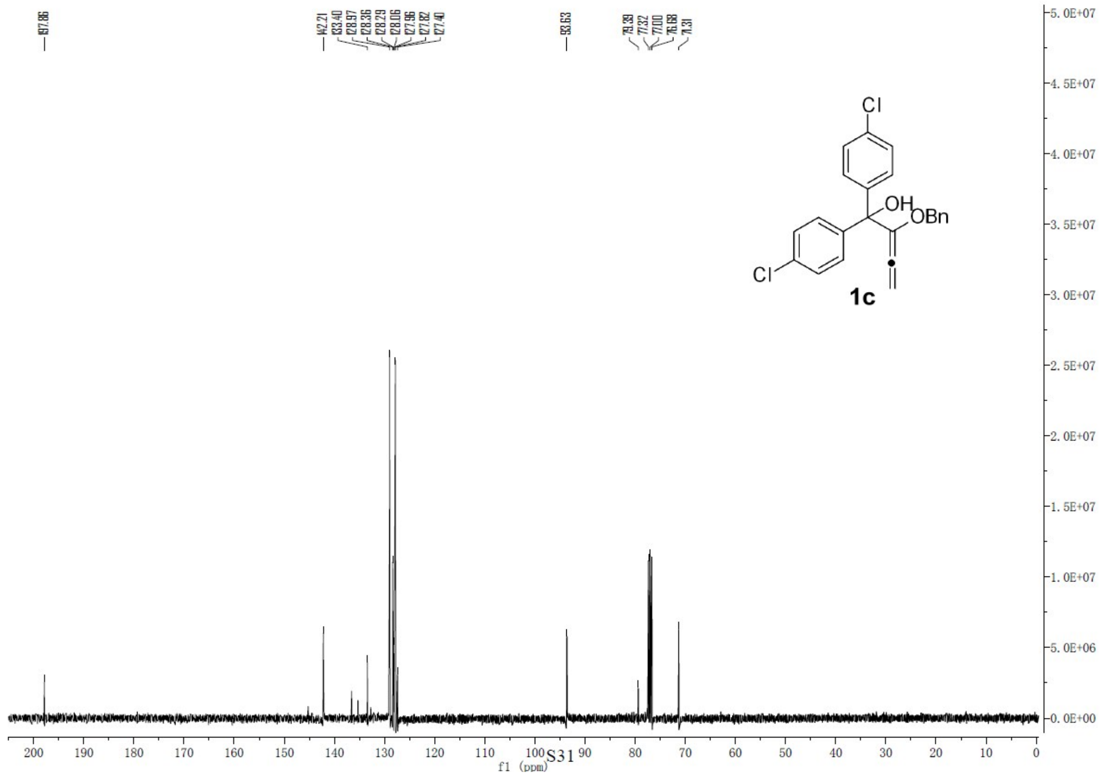
1c
f1 (ppm)

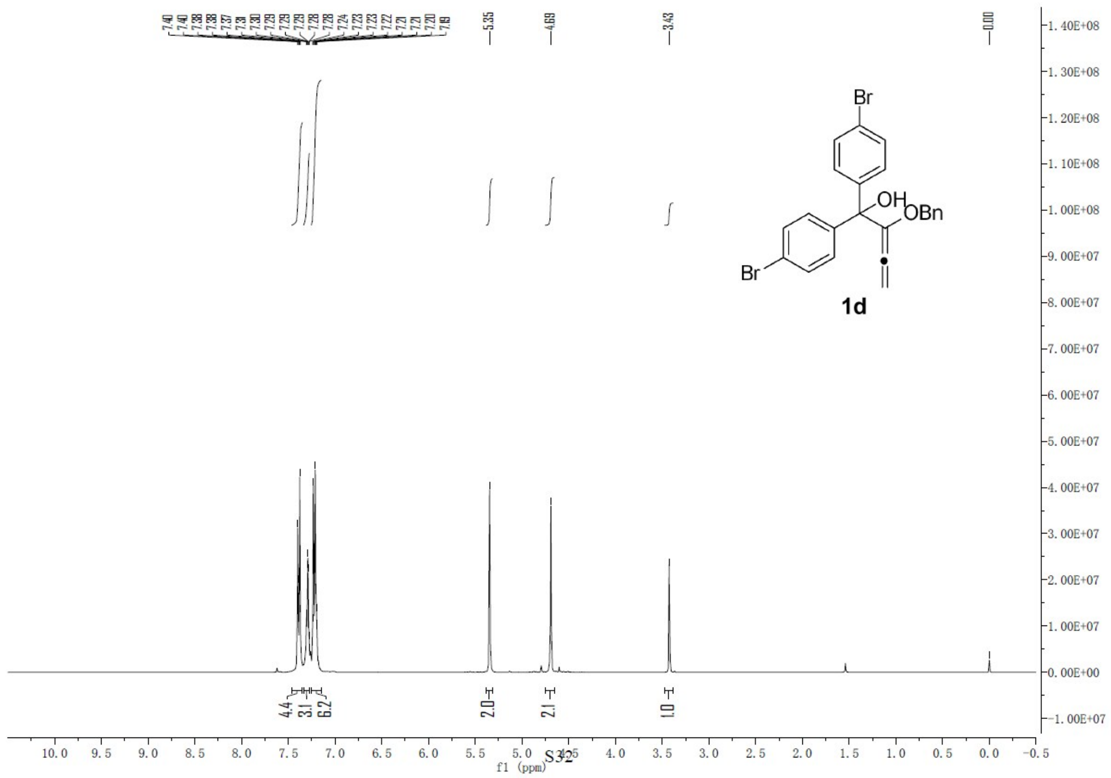

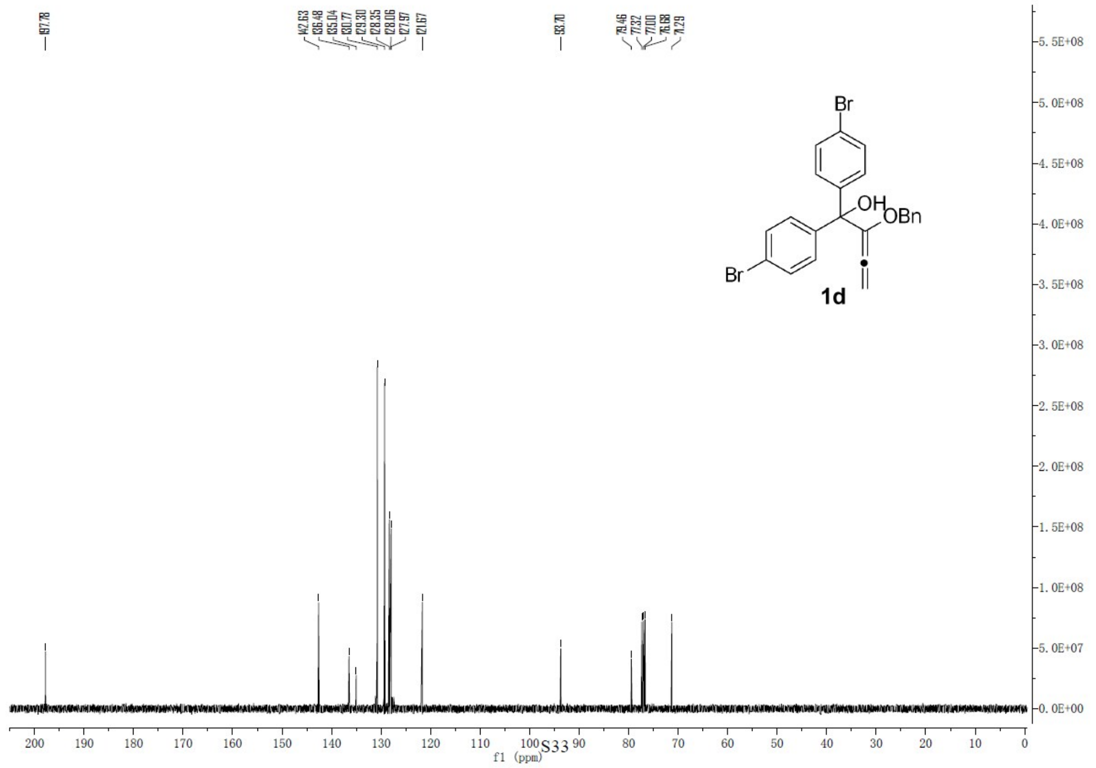

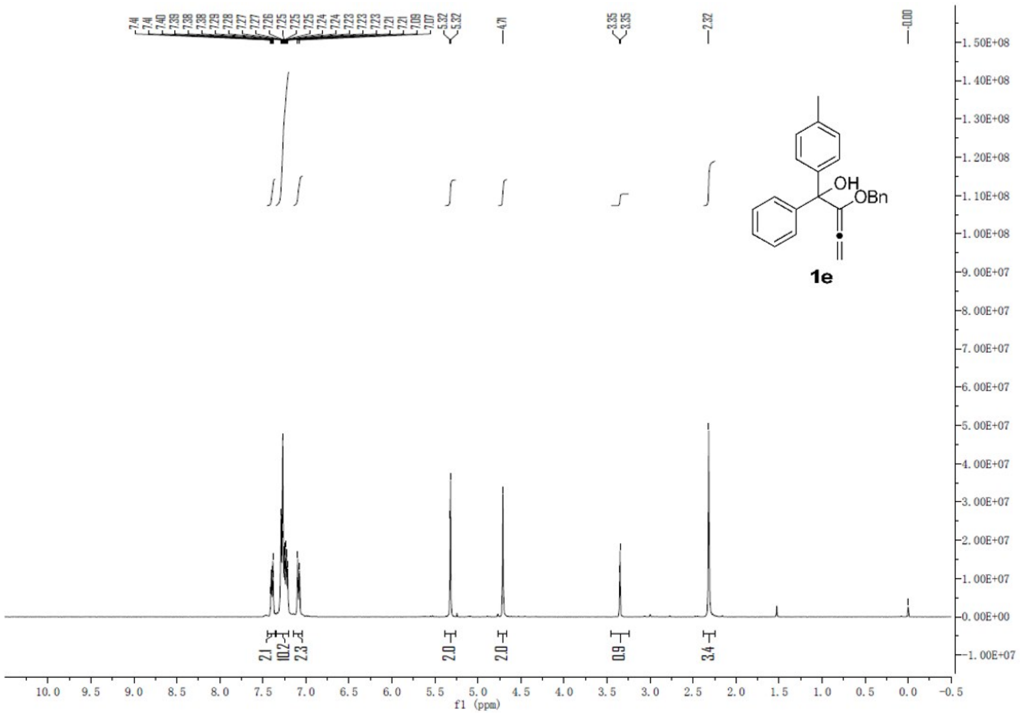
1e
f1 (ppm)

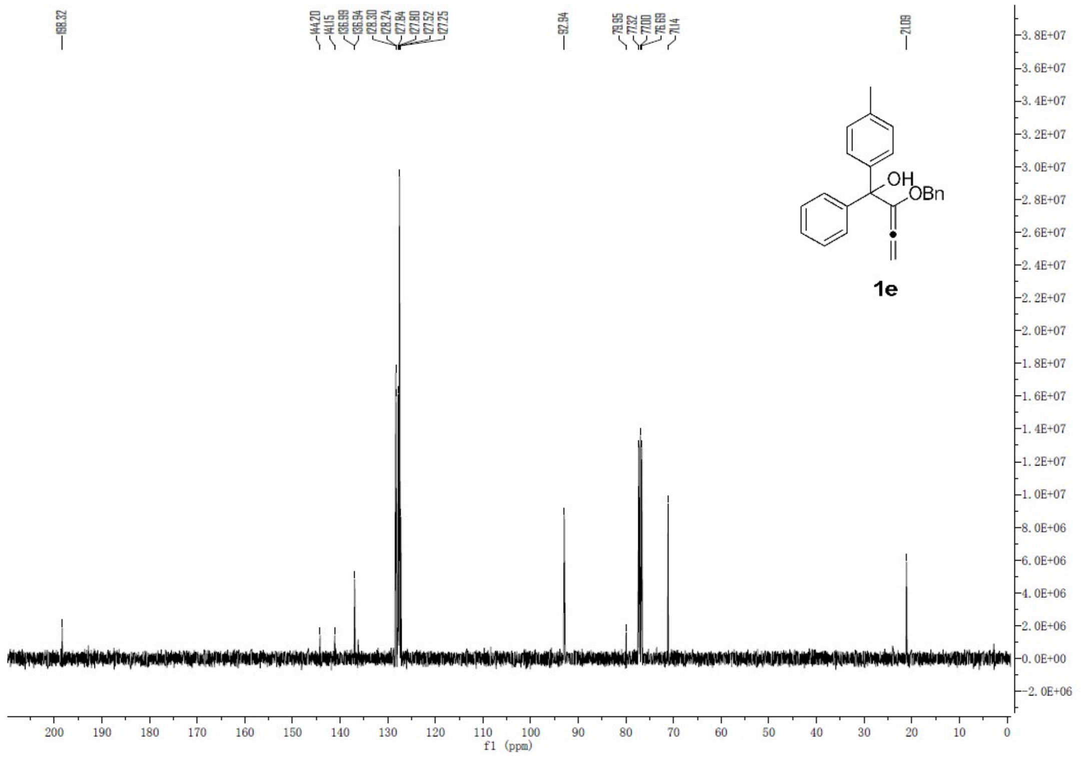
1e
f1 (ppm)

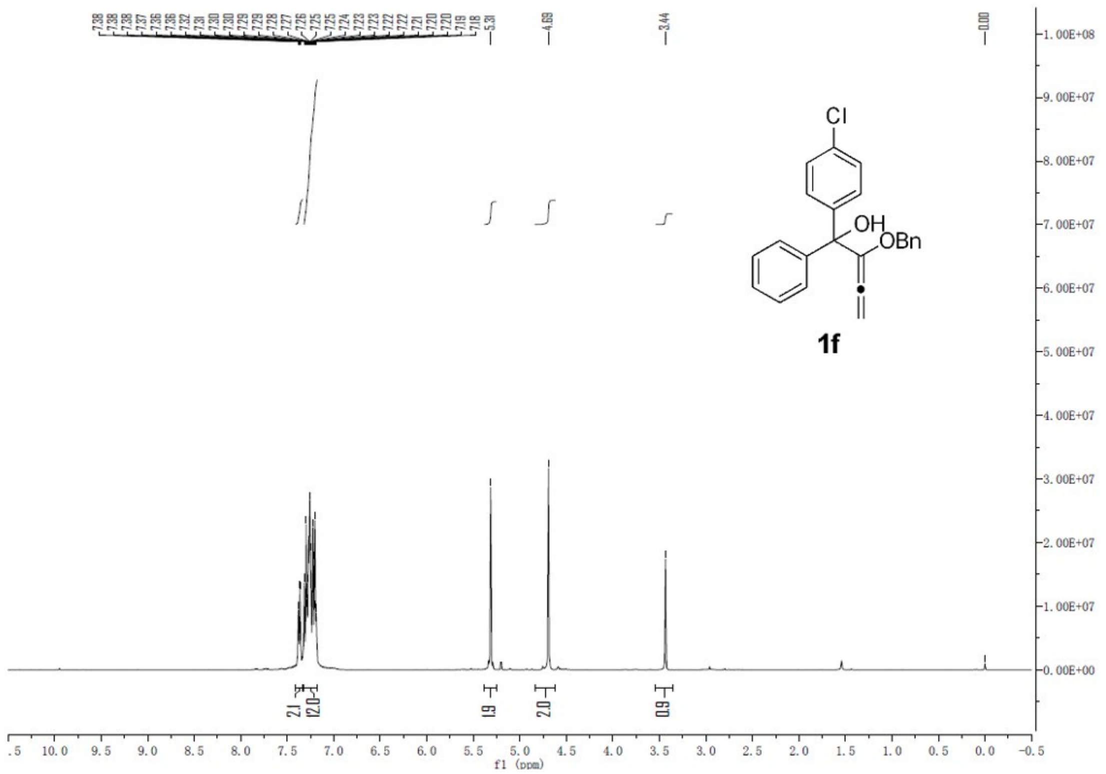
1f
OH
OBn
Cl
f1 (ppm)

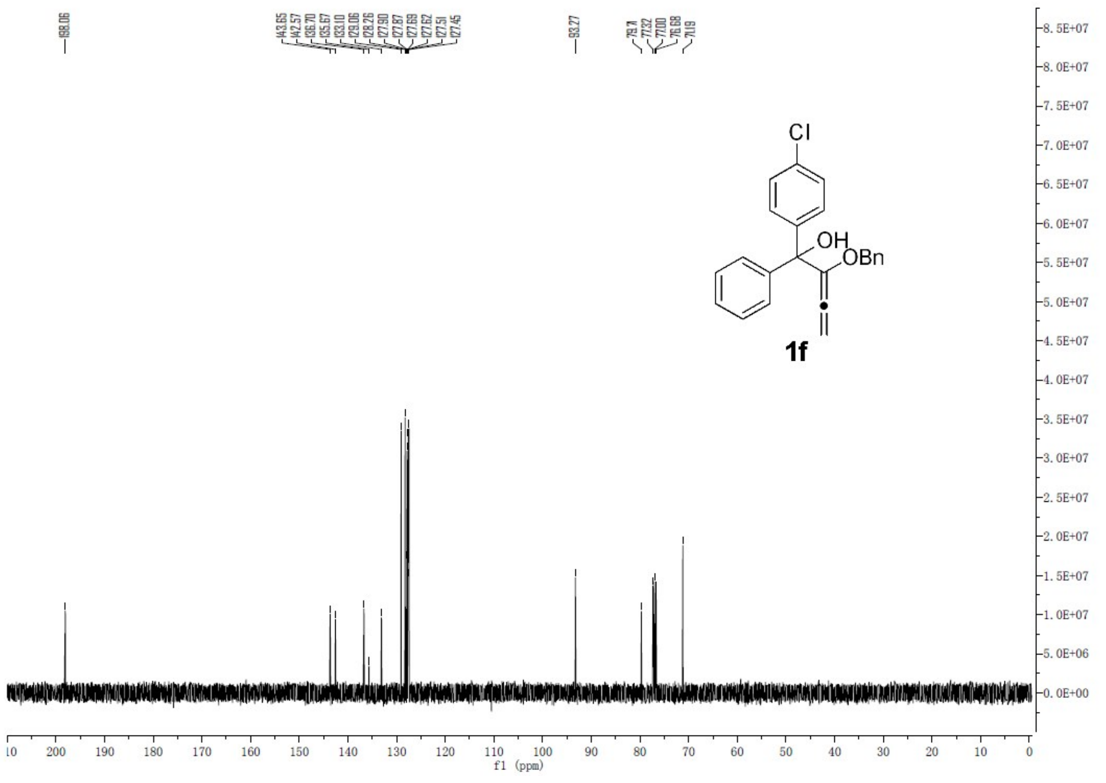
1f
OH
OBn
Cl
f1 (ppm)

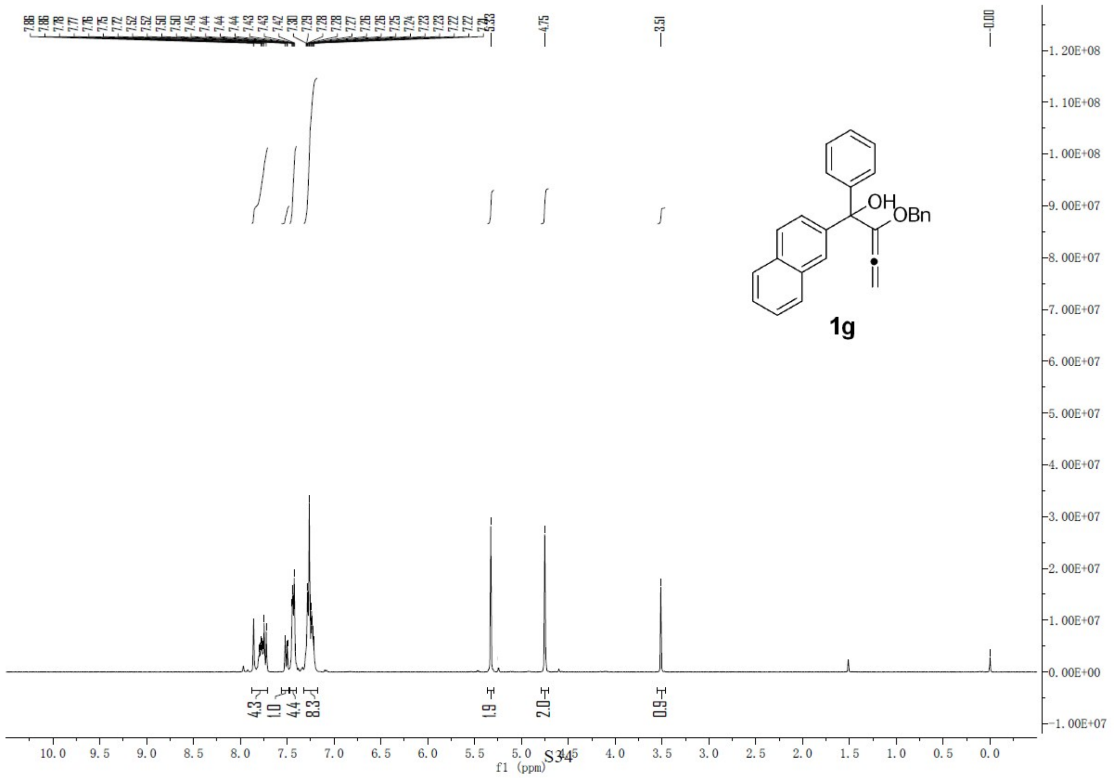
OH
OBn
1g
f1 (ppm)

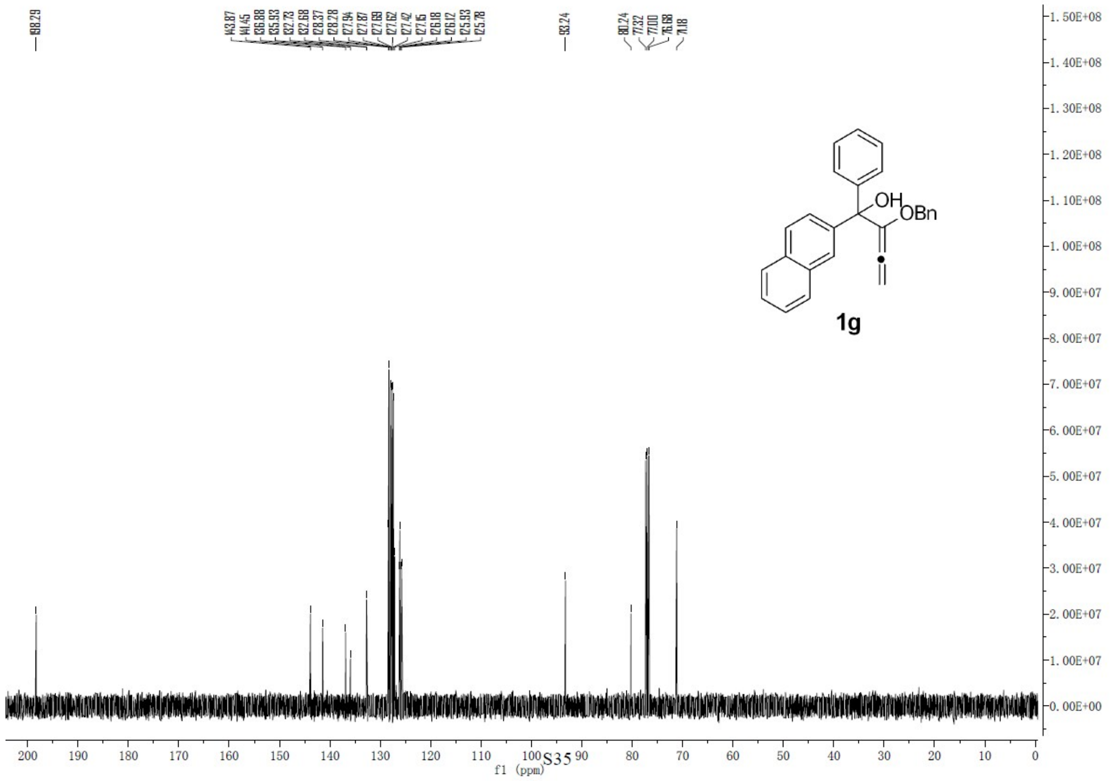
OH
OBn
1g
f1 (ppm)

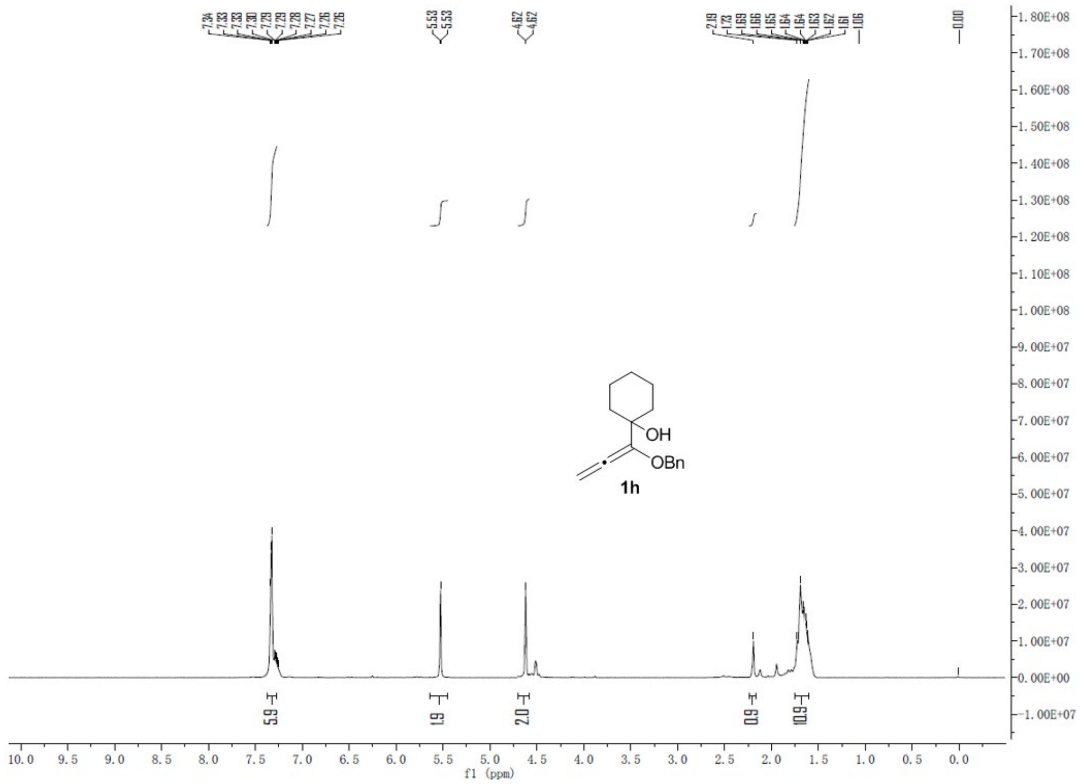

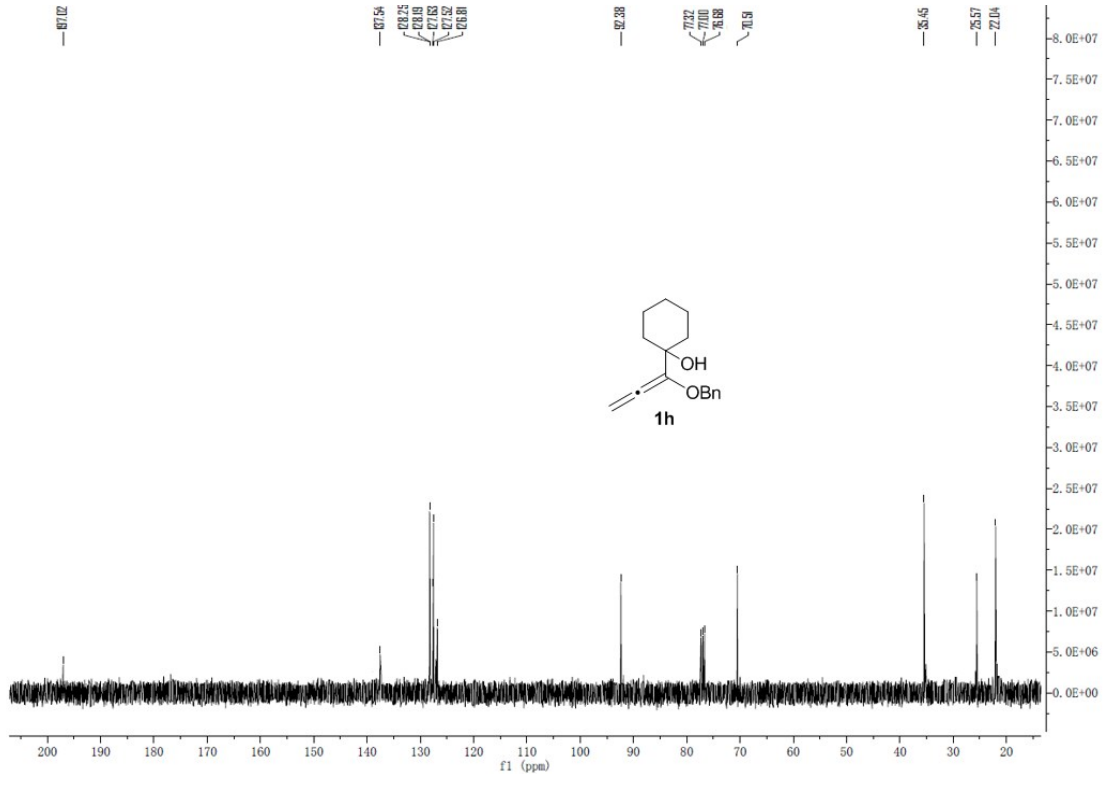

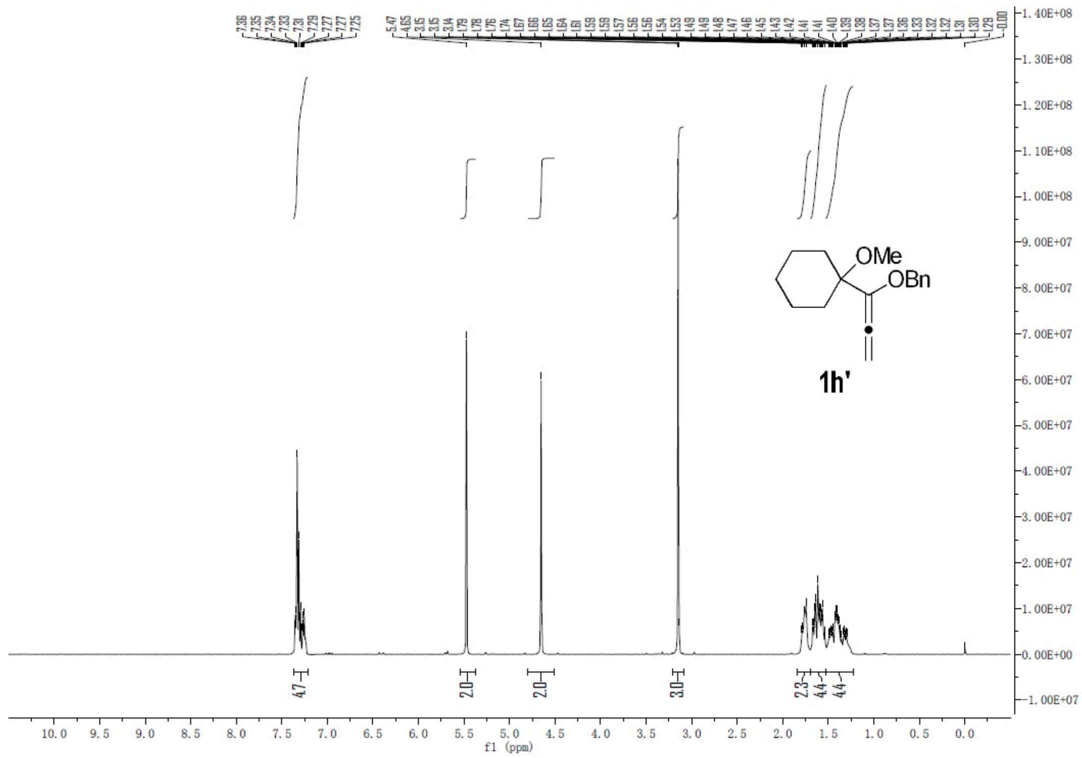

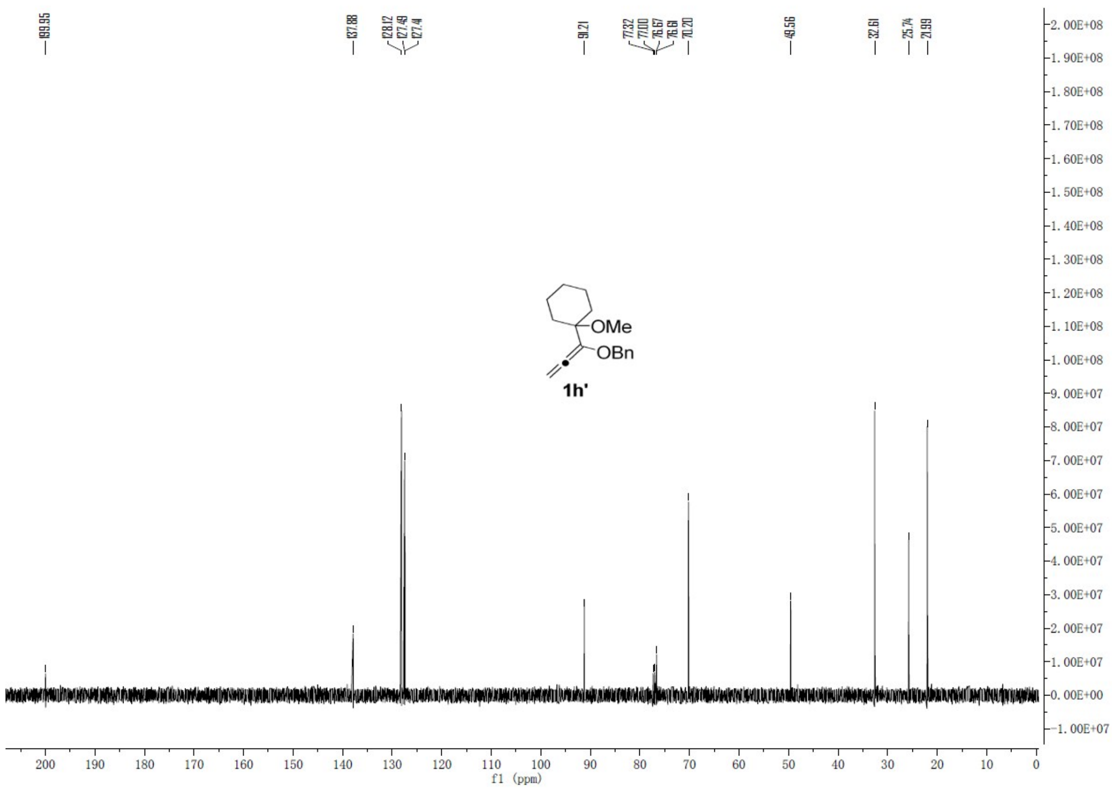

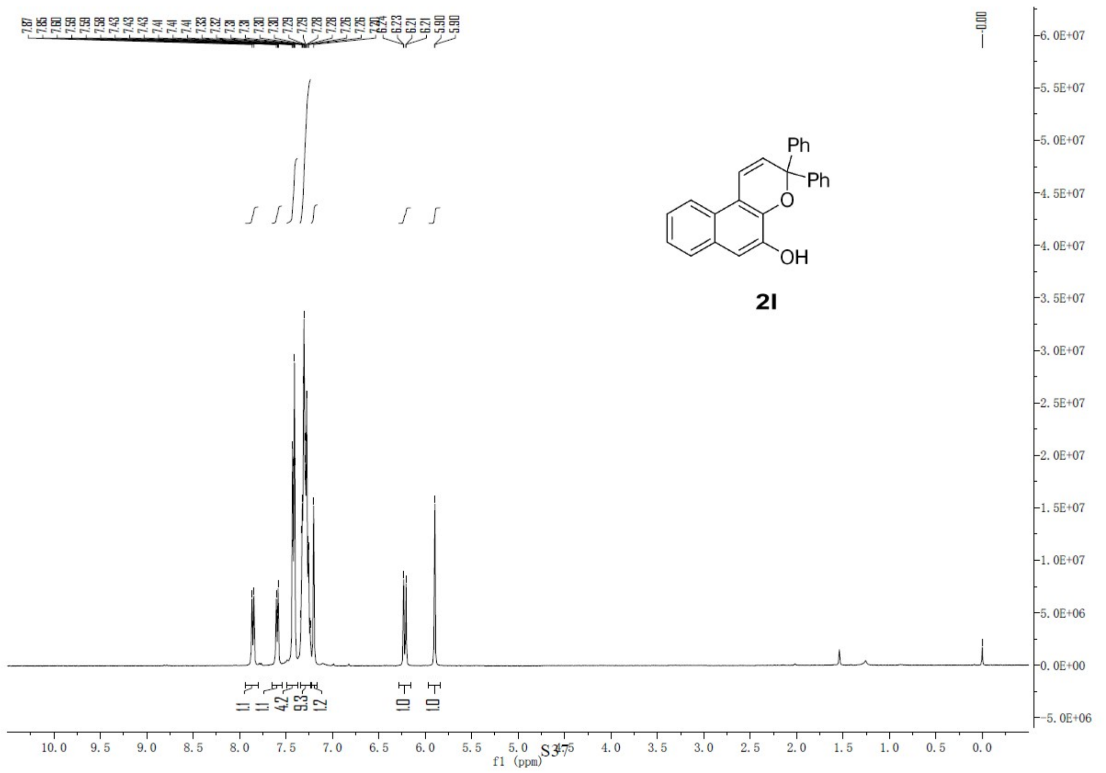
2l
f1 (ppm)

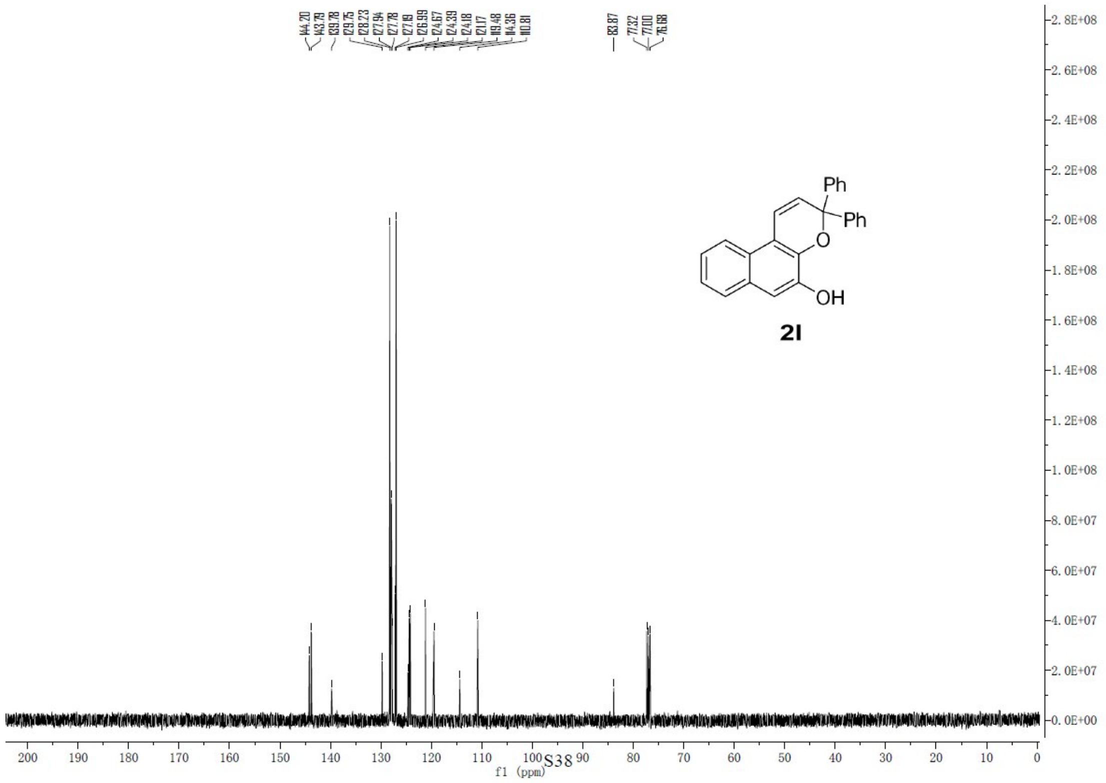
2l
f1 (ppm)

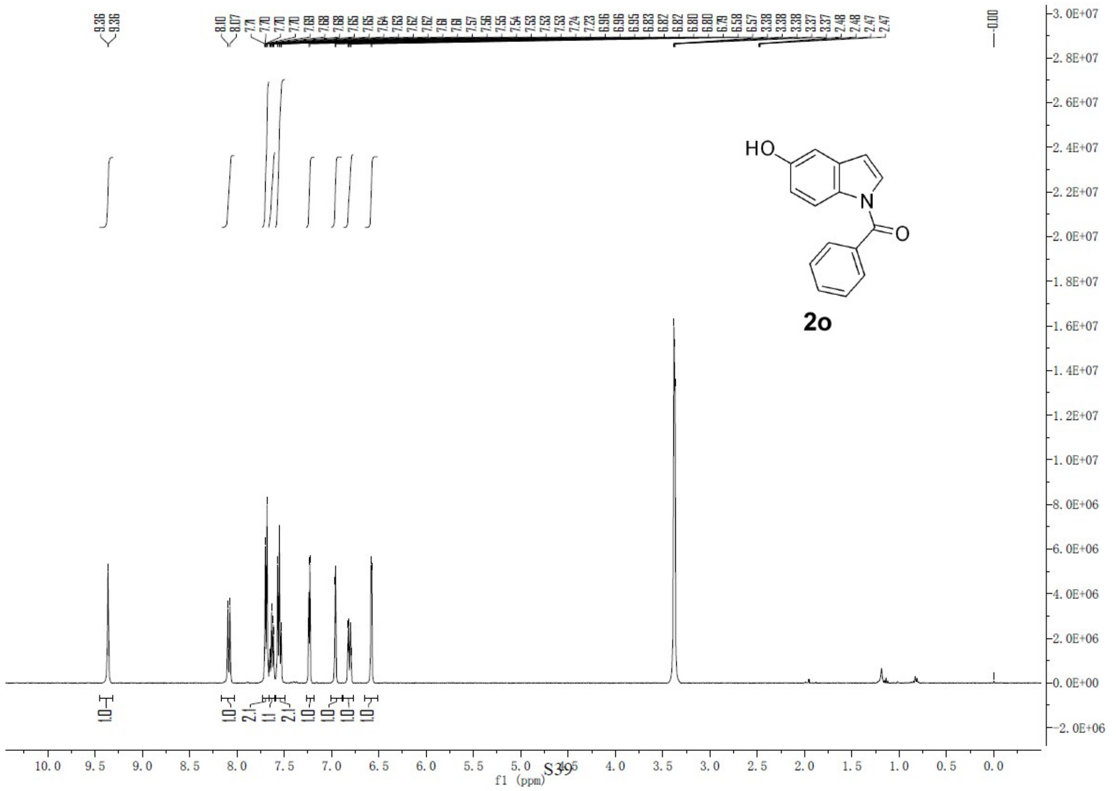
HO
N
O
2o
f1 (ppm)
S39

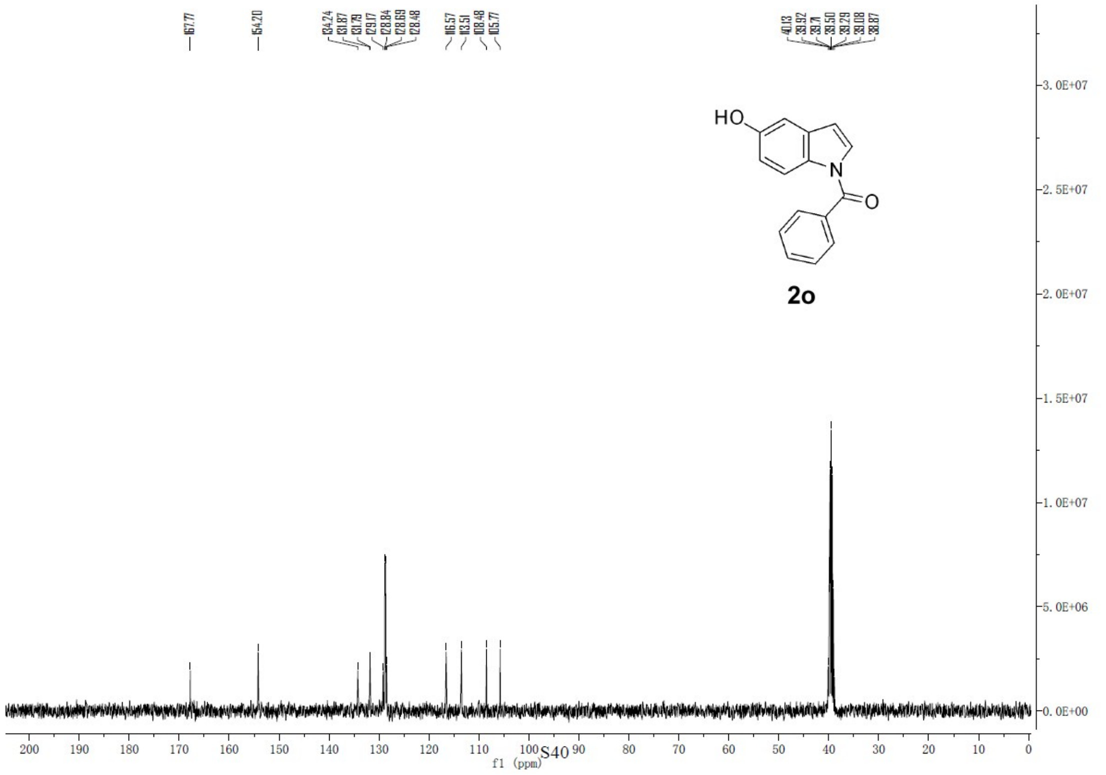
HO
N
O
2o
f1 (ppm)
S40

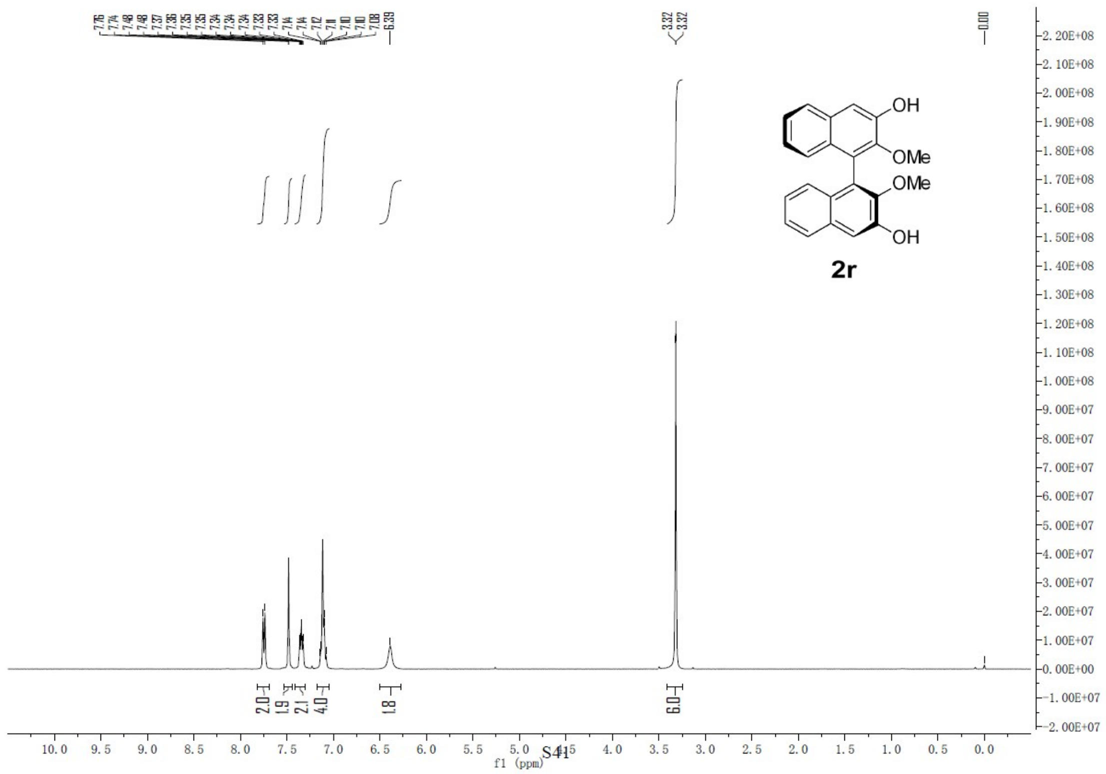
OH
OMe
OMe
OH
2r
f1 (ppm)
S41

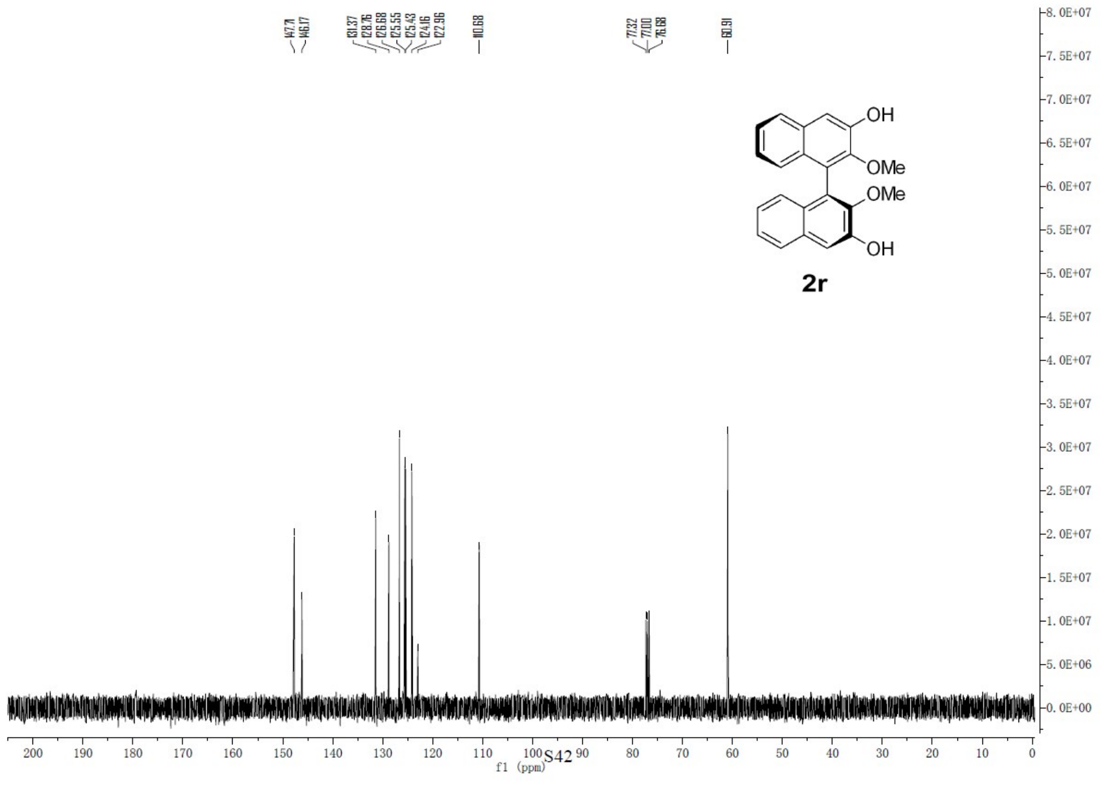
OH
OMe
OMe
OH
2r
f1 (ppm)
S42

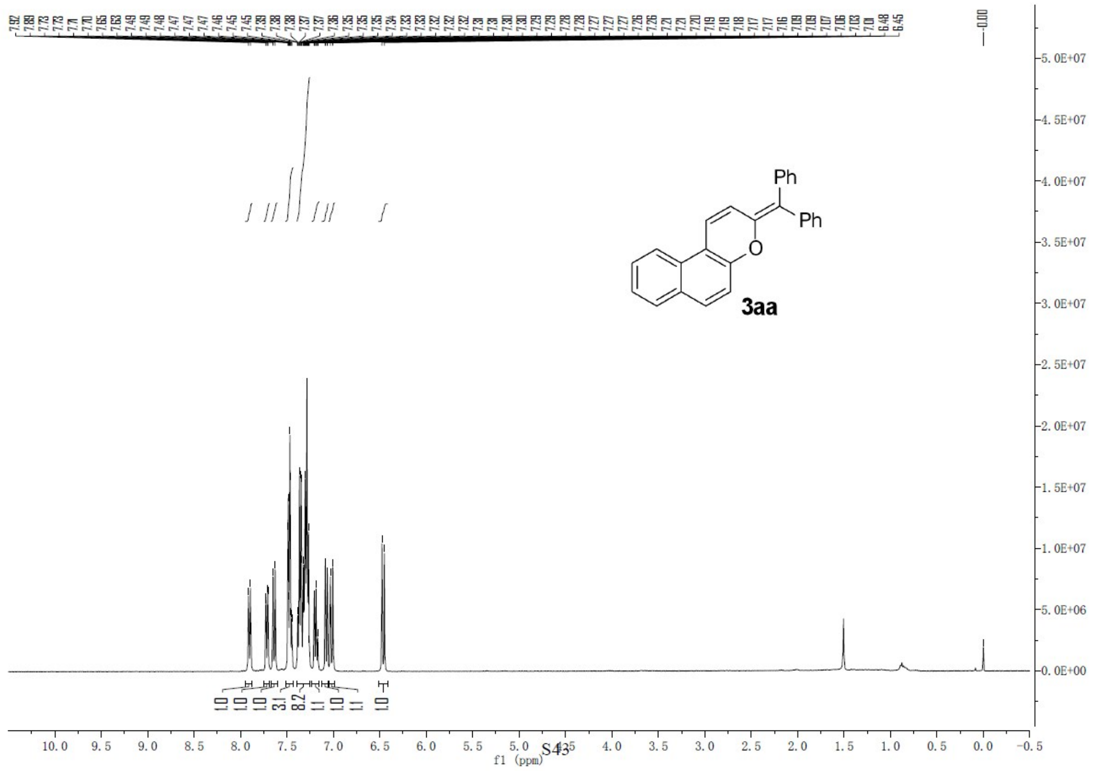
Ph
Ph
O
3aa
f1 (ppm)
S43

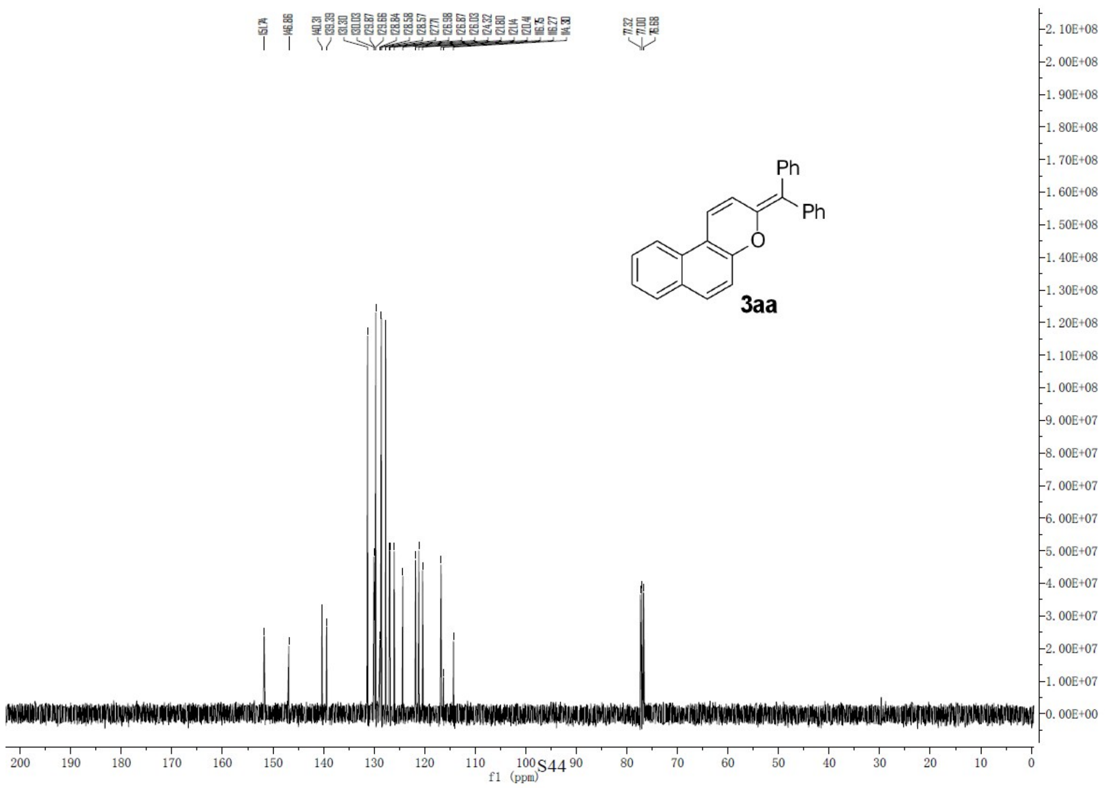
Ph
Ph
O
3aa
f1 (ppm)
S44

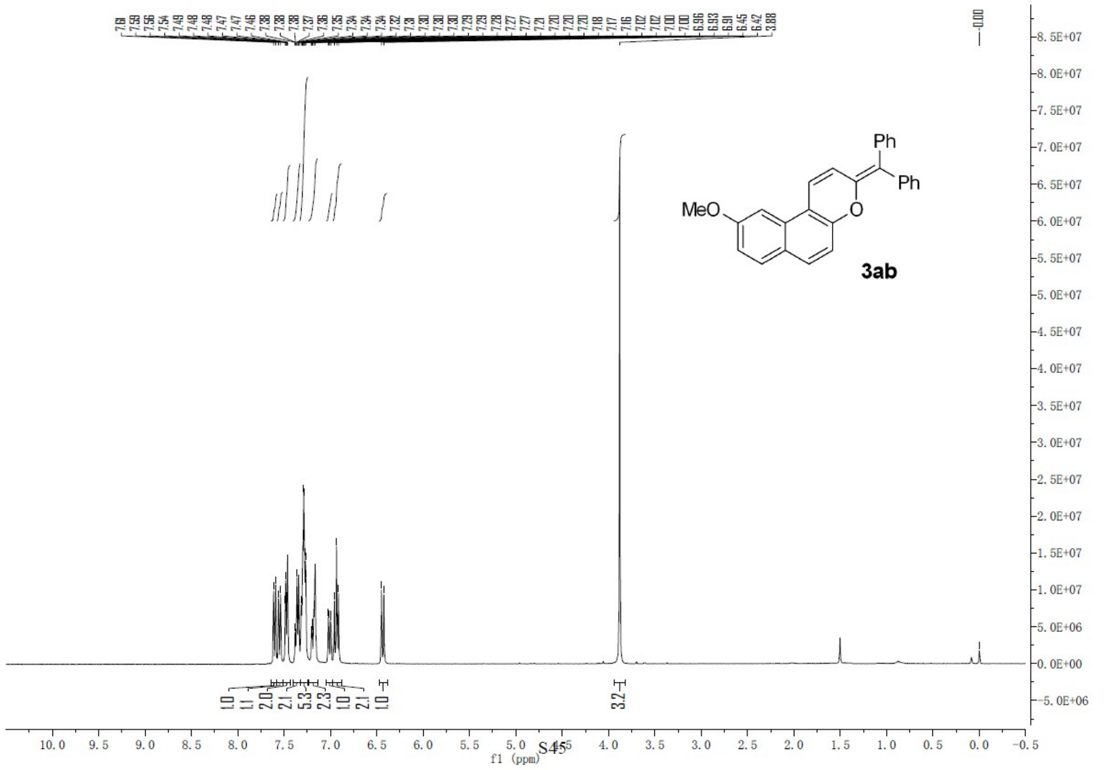

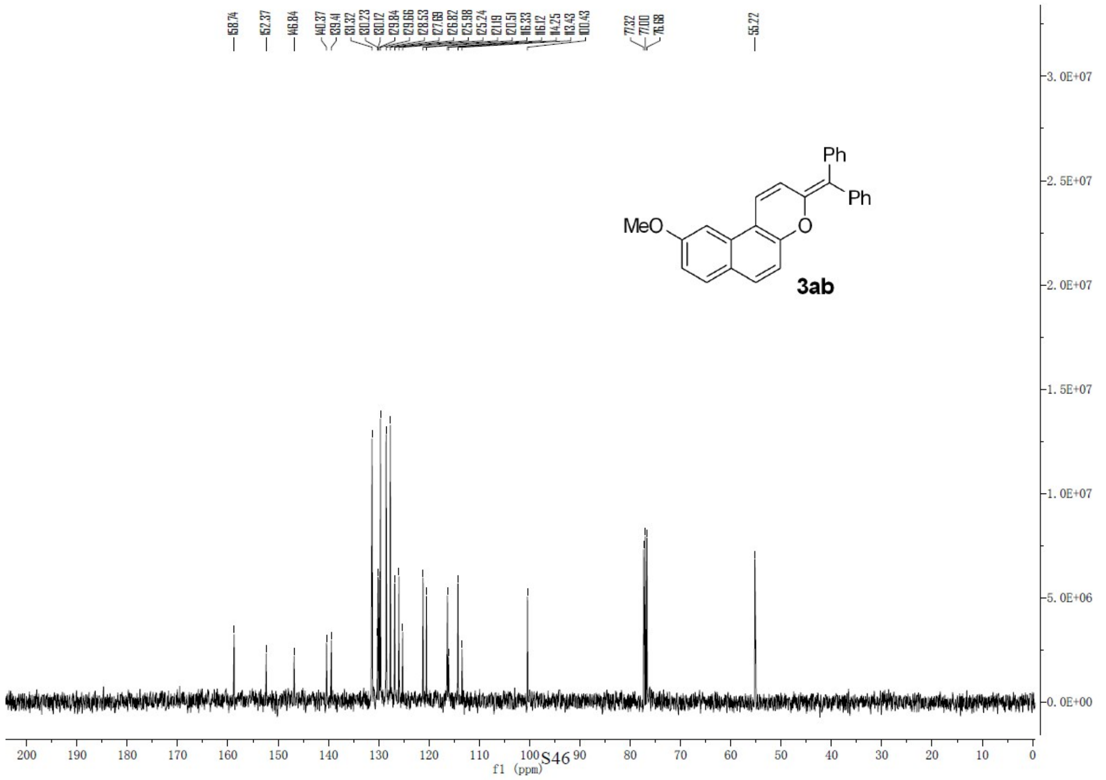

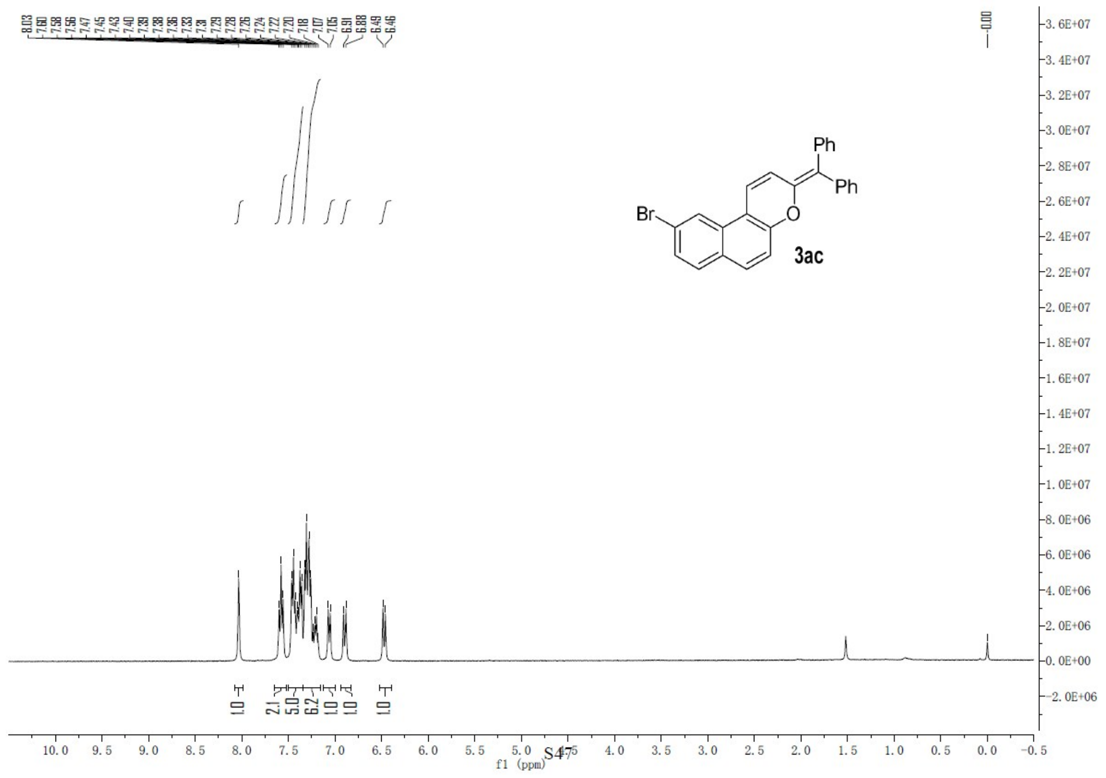

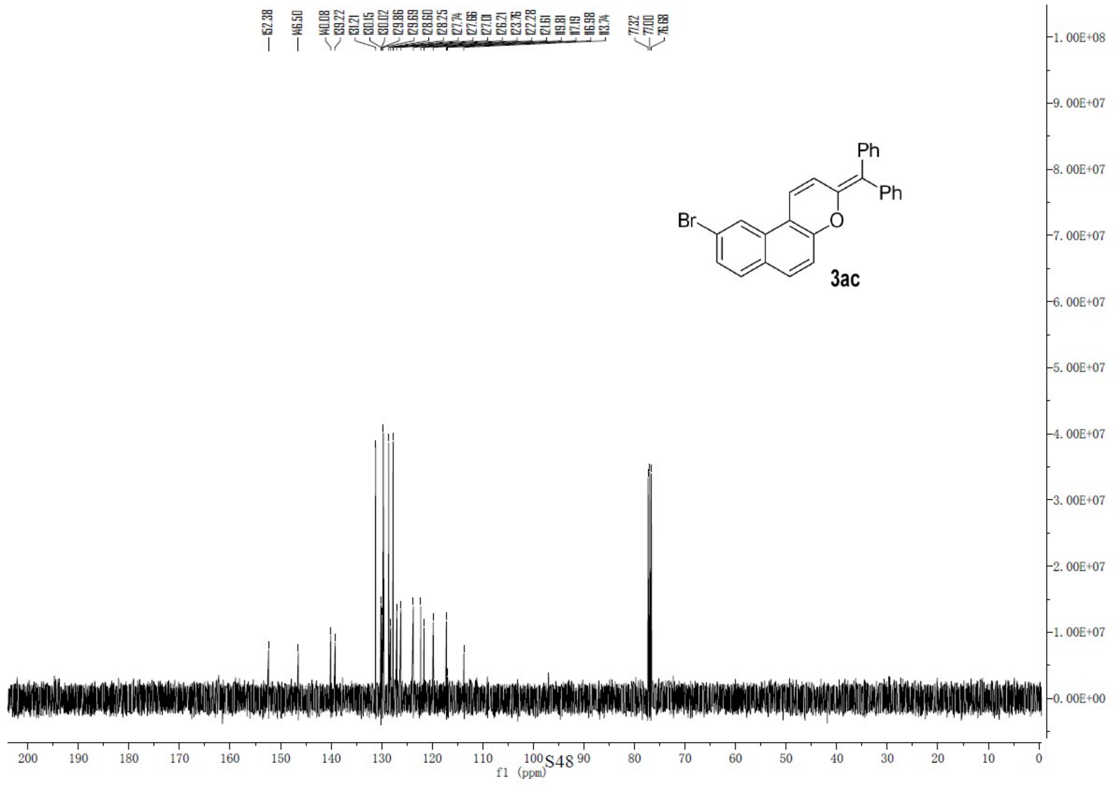

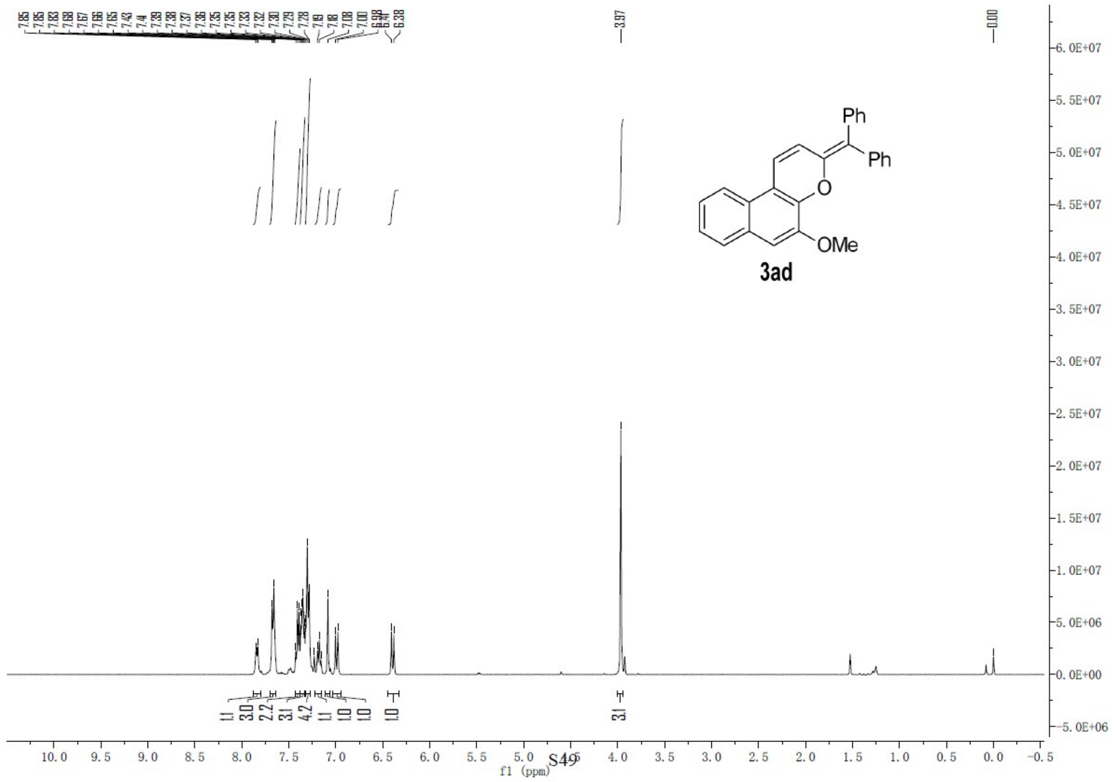

Ph
Ph
O
OMe
3ad
f1 (ppm)
S49

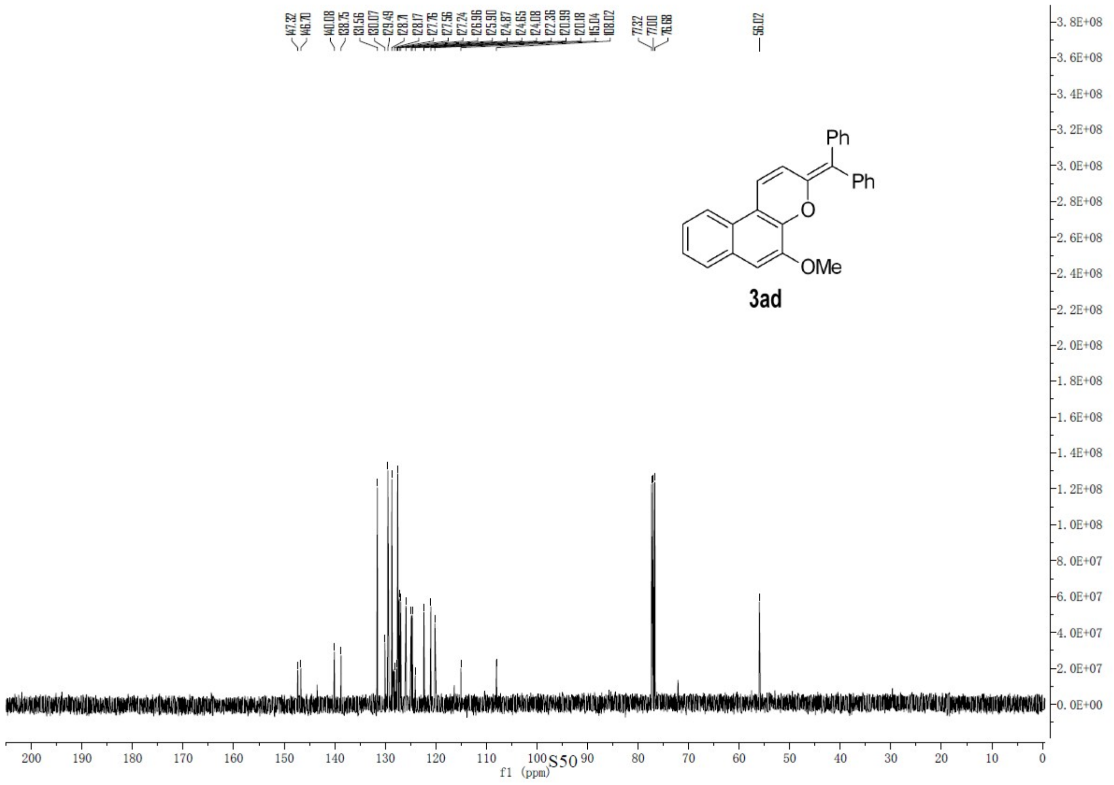

Ph
Ph
O
OMe
3ad
f1 (ppm)
S50

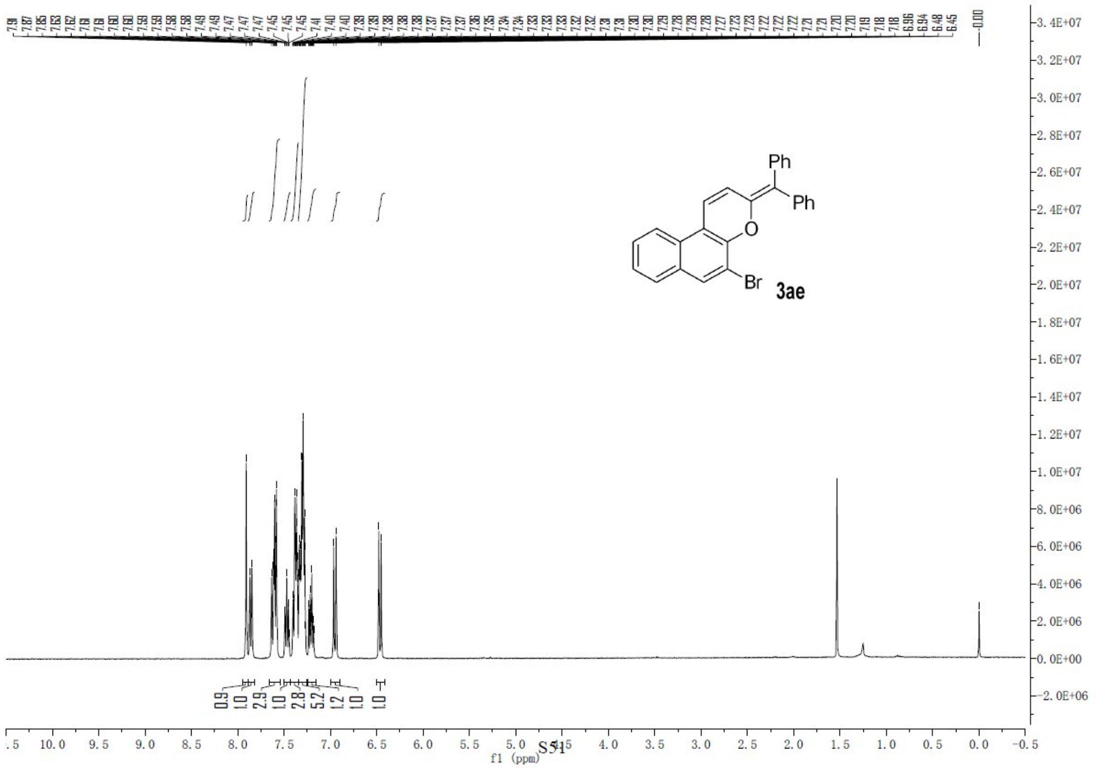
Ph
Ph
O
Br
3ae
f1 (ppm)
S51

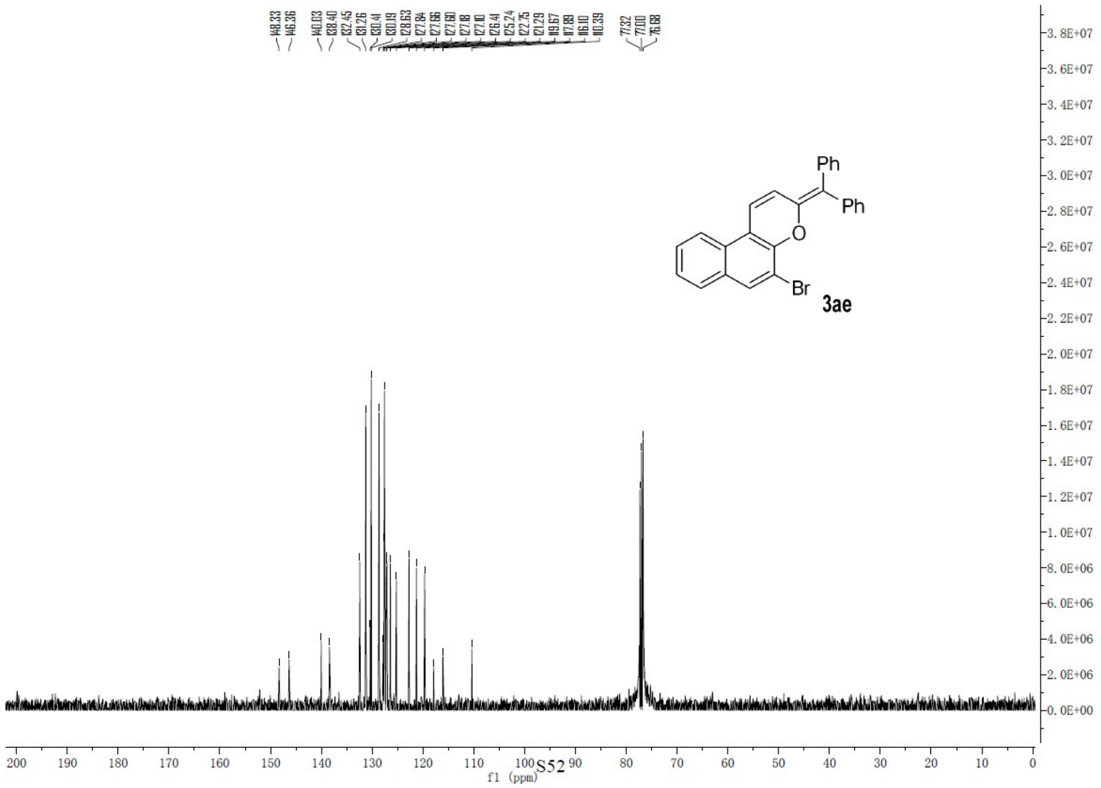
Ph
Ph
O
Br
3ae
f1 (ppm)
S52

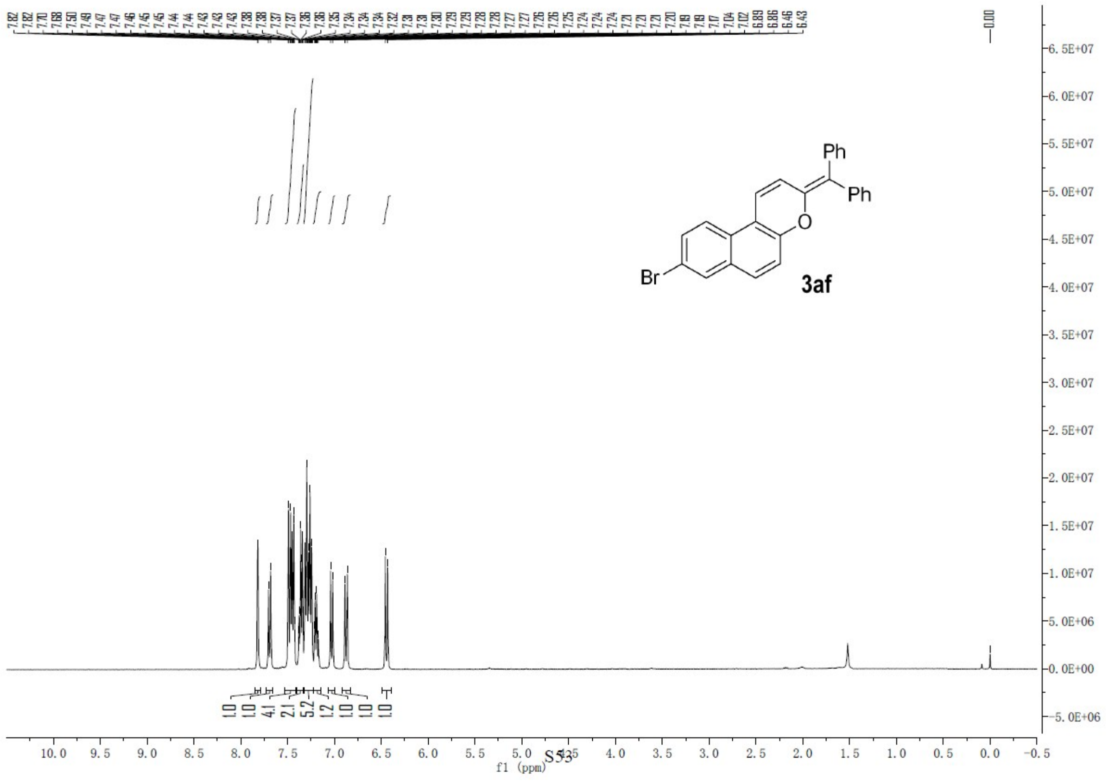

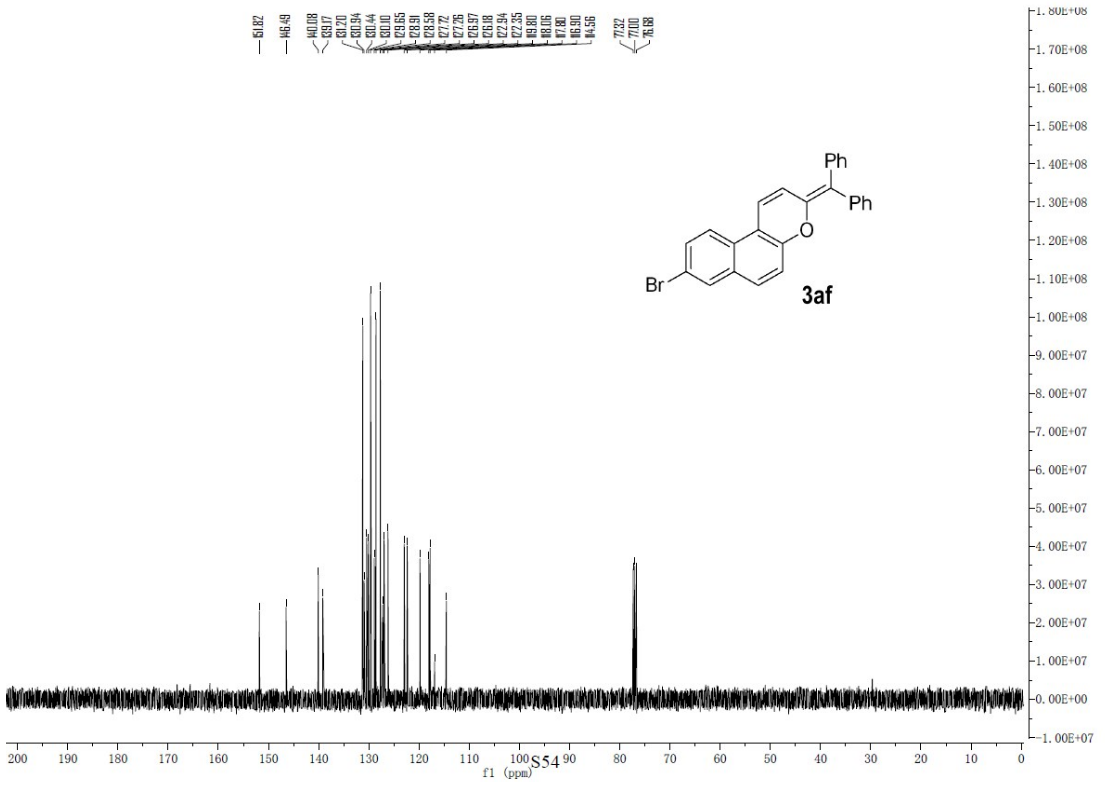

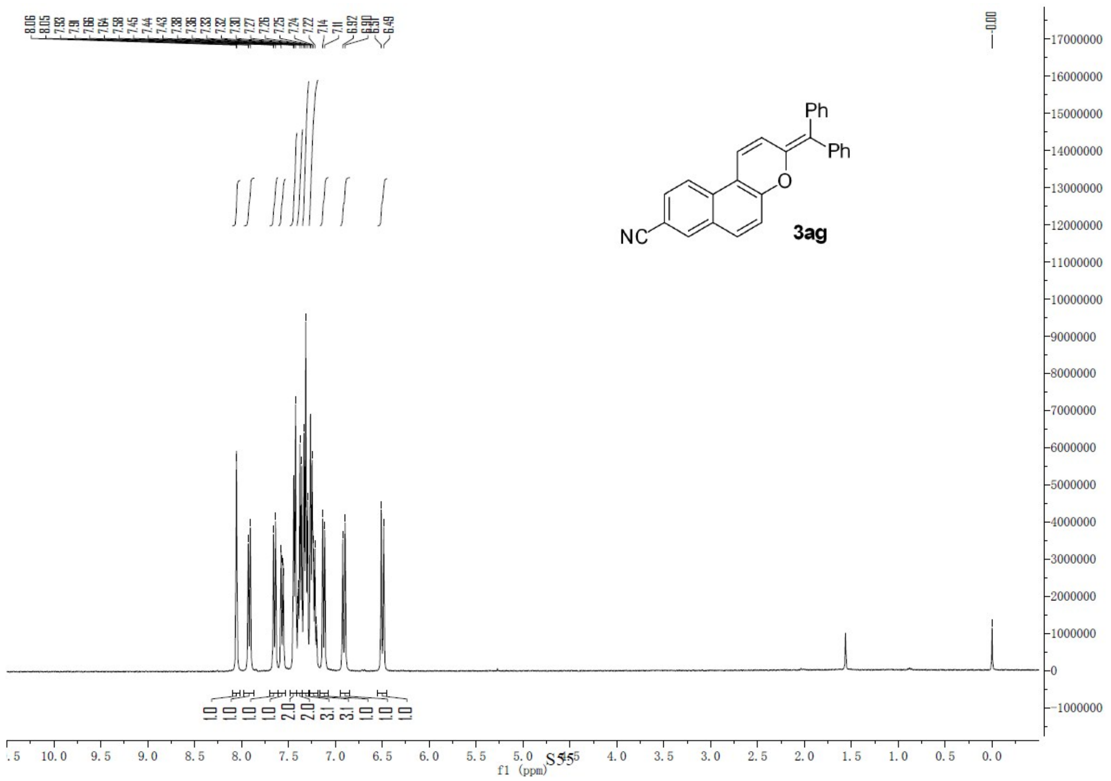
3ag
f1 (ppm)

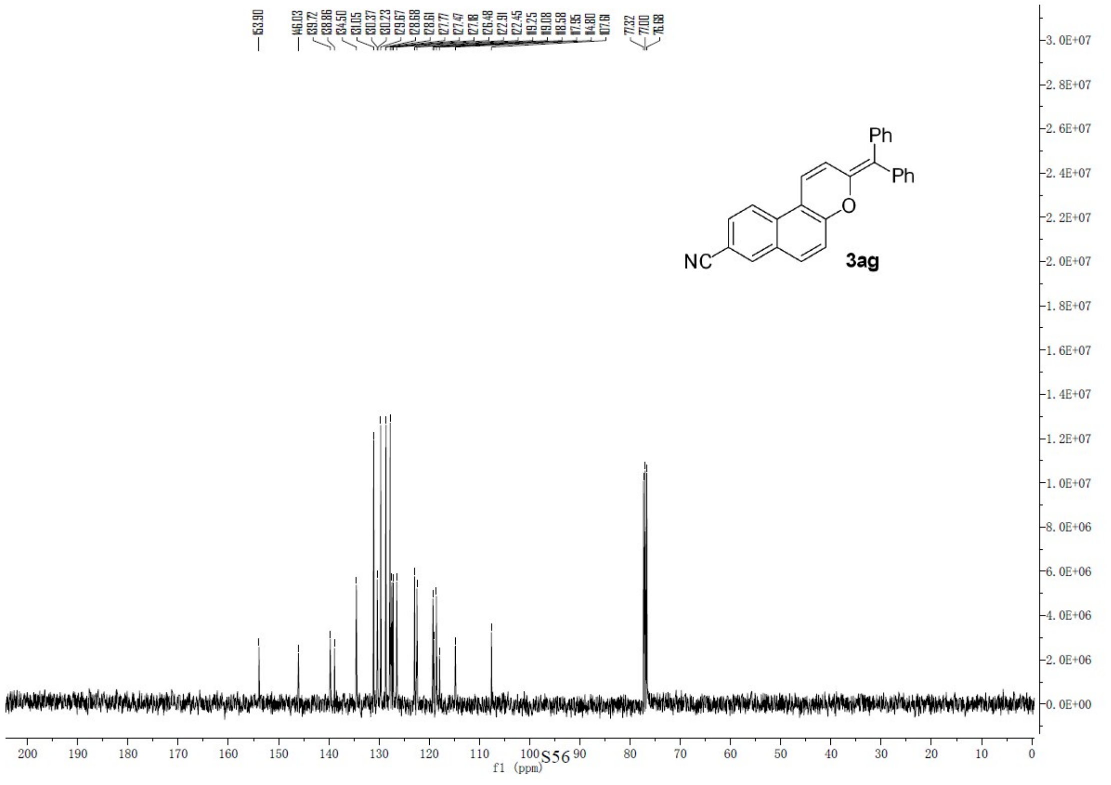
3ag
f1 (ppm)

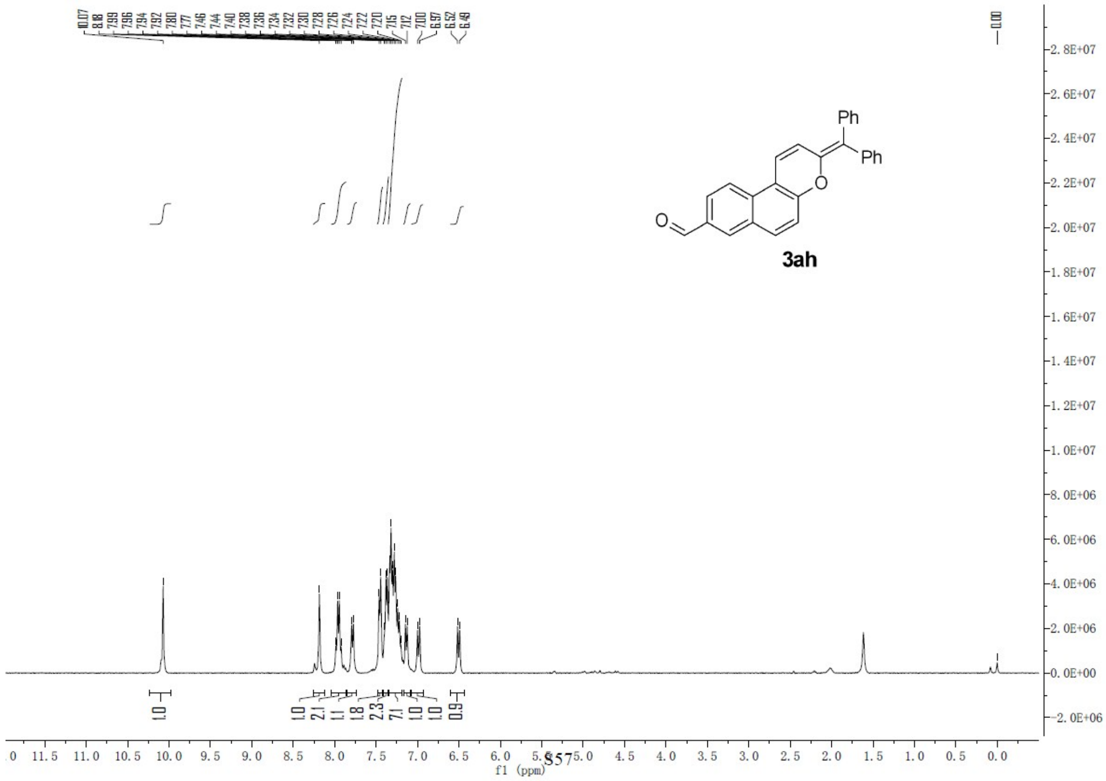
3ah
f1 (ppm)
S57

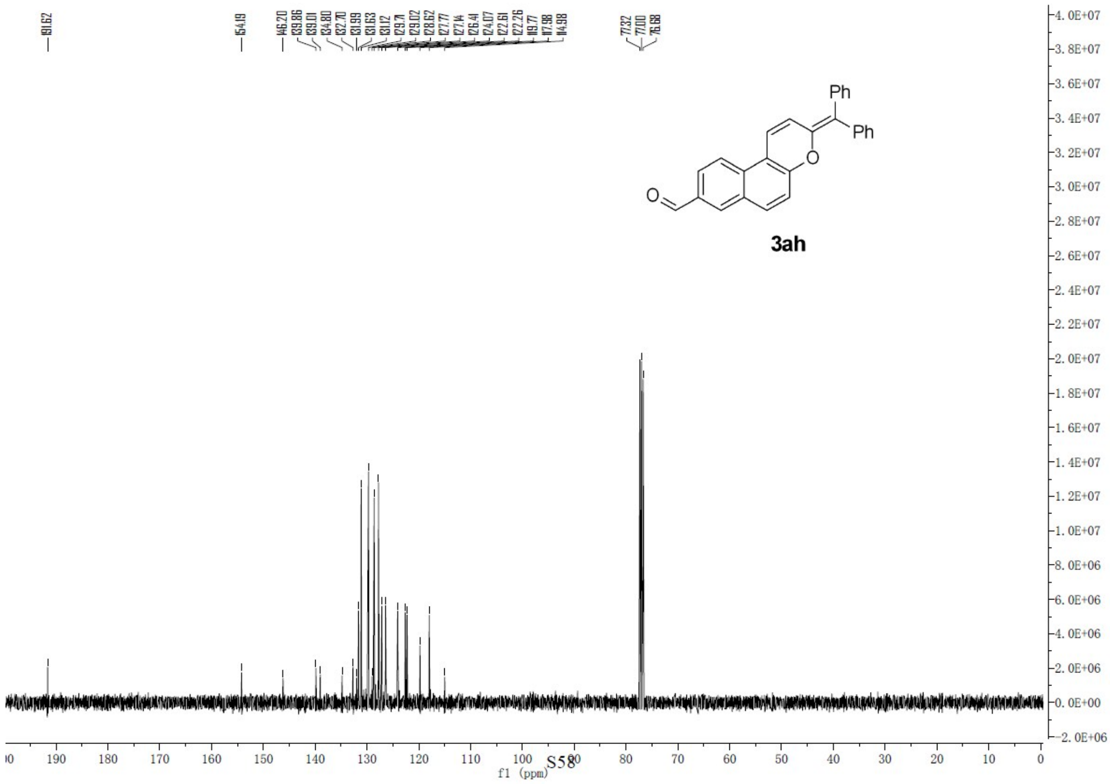
3ah
f1 (ppm)
S58

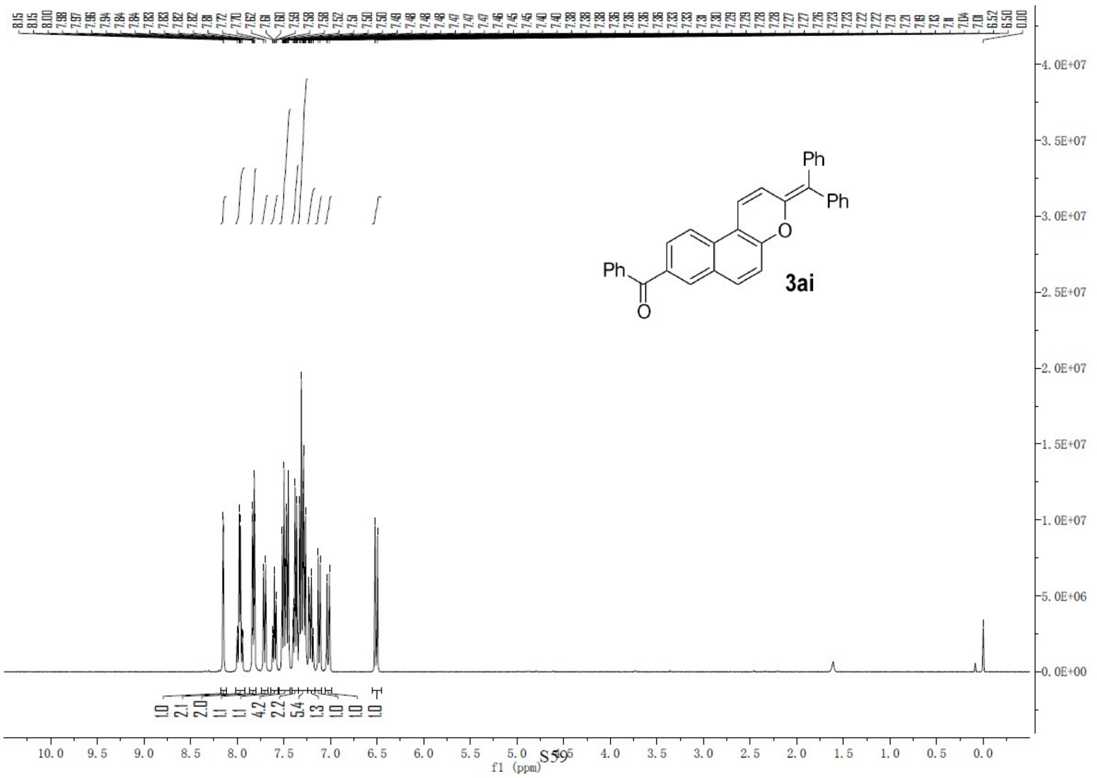
Ph
Ph
O
Ph
O
3ai
f1 (ppm)

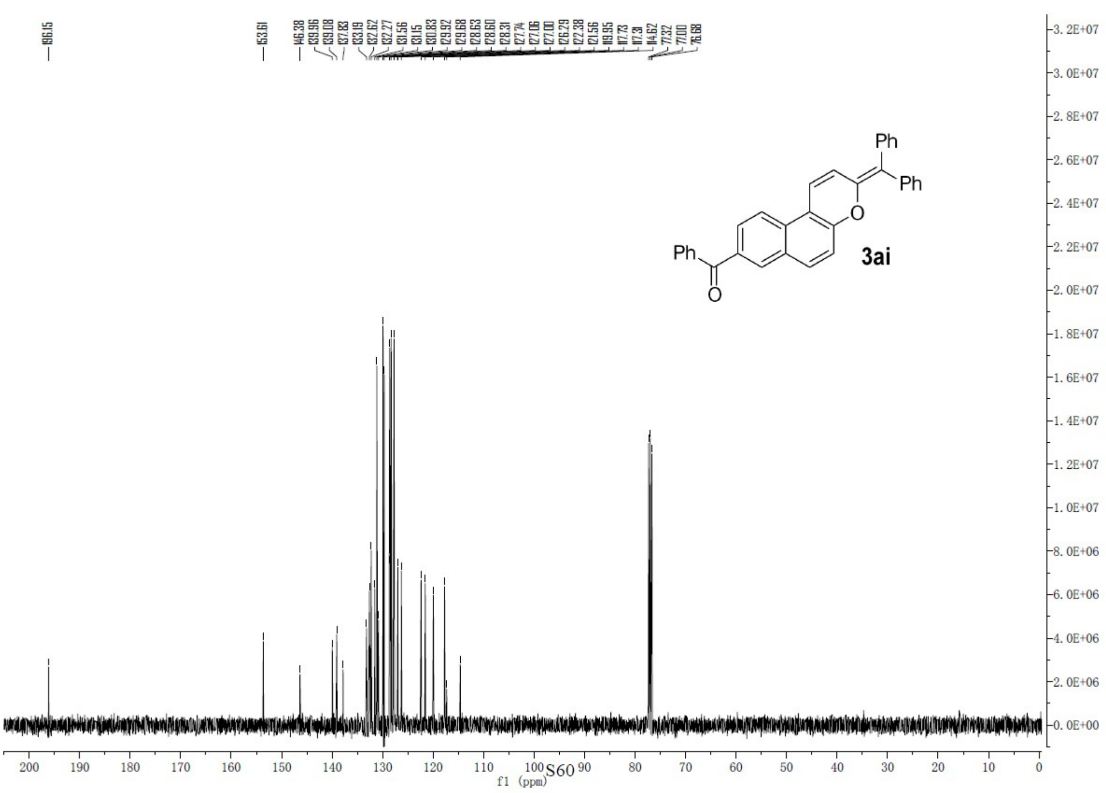
Ph
Ph
O
Ph
O
3ai
f1 (ppm)

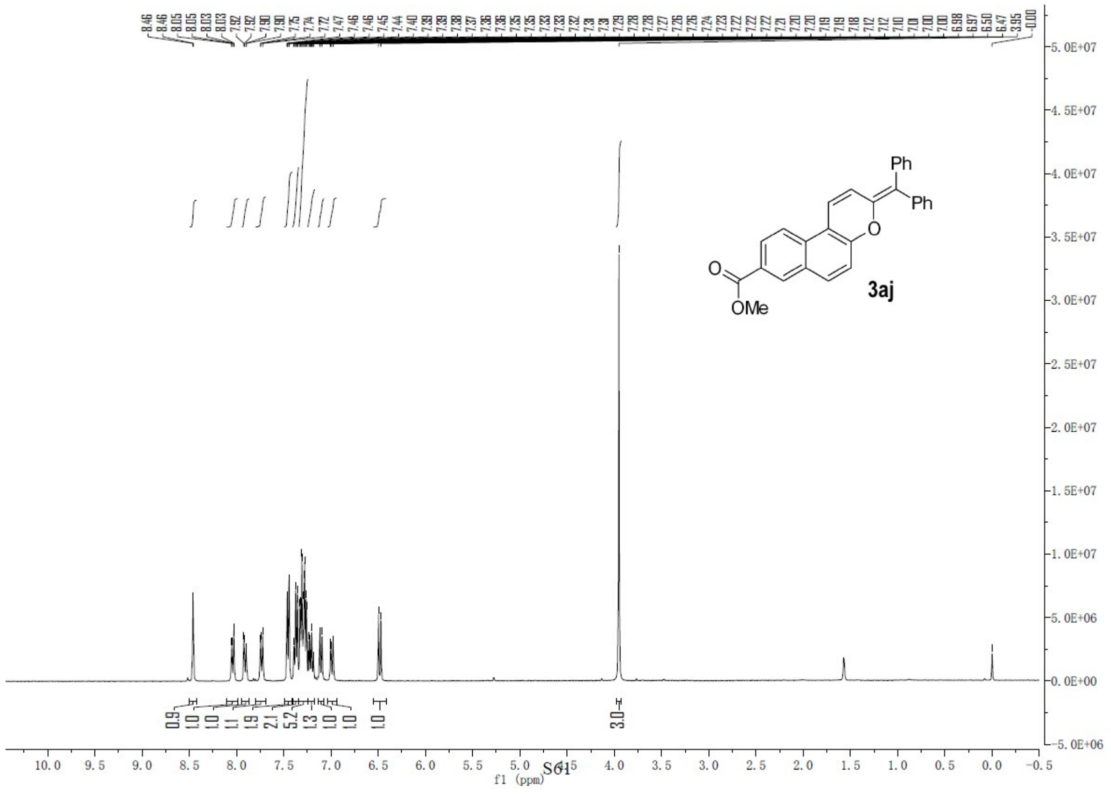

3aj
f1 (ppm)

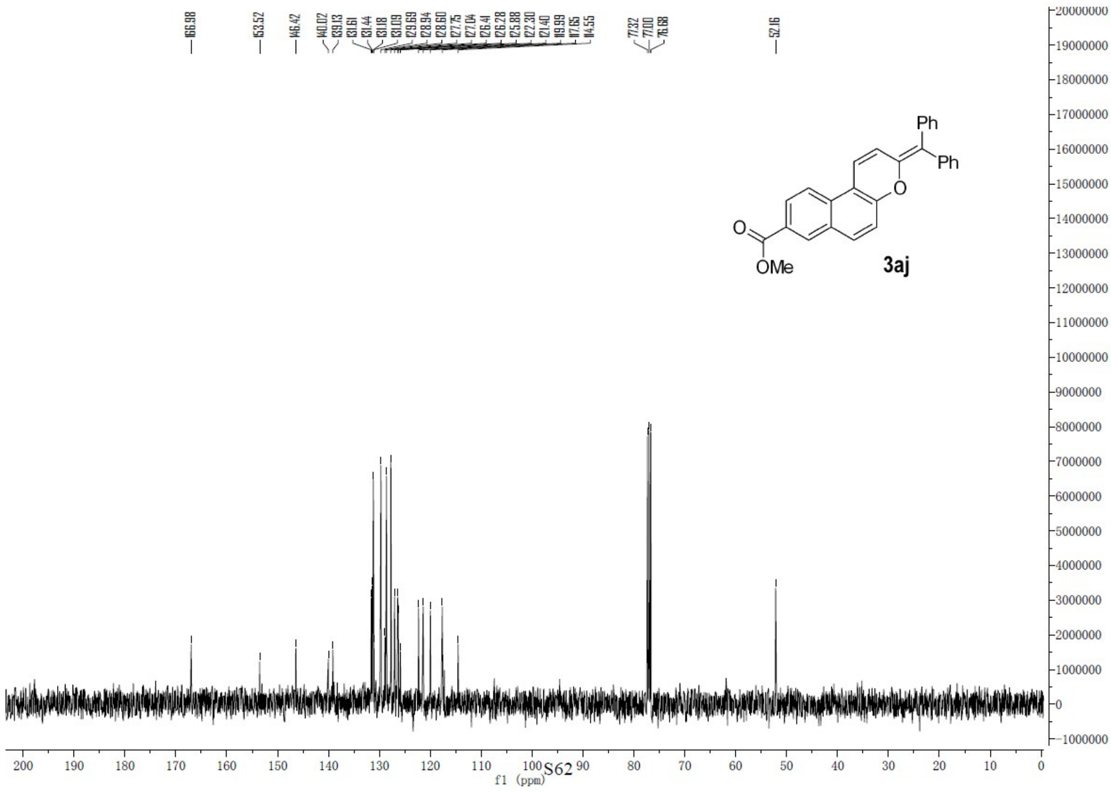

3aj
f1 (ppm)

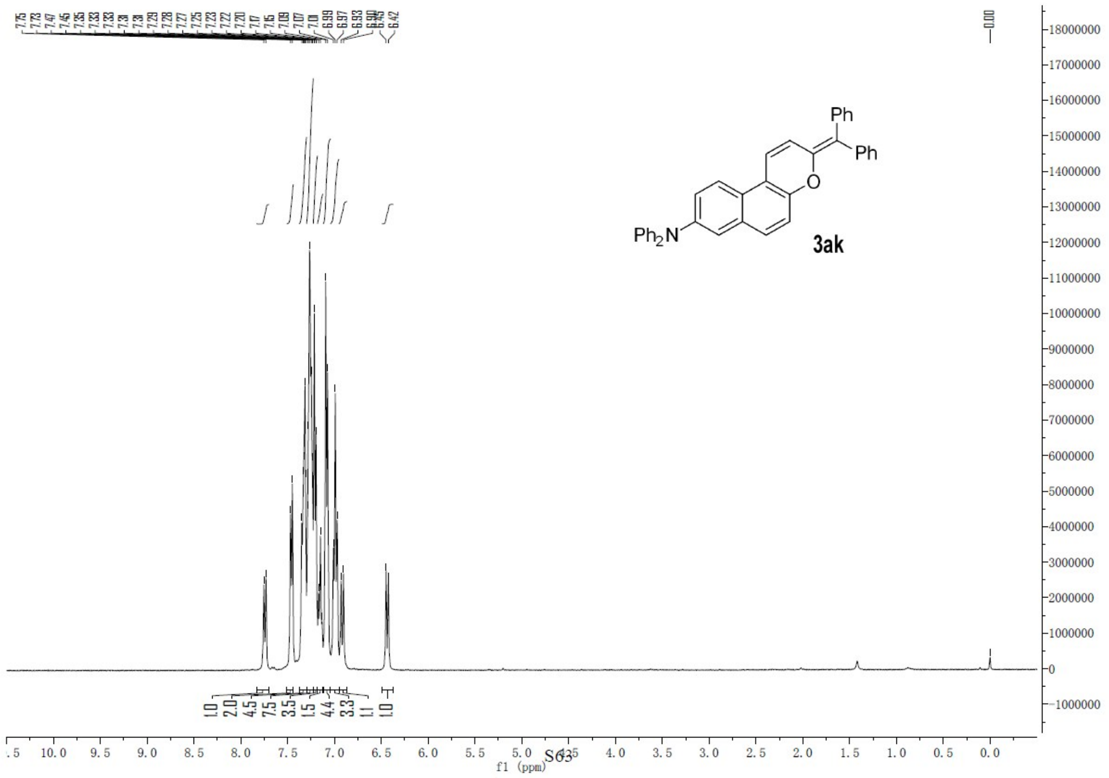
Ph
Ph
O
Ph_2N
3ak
f1 (ppm)
S63

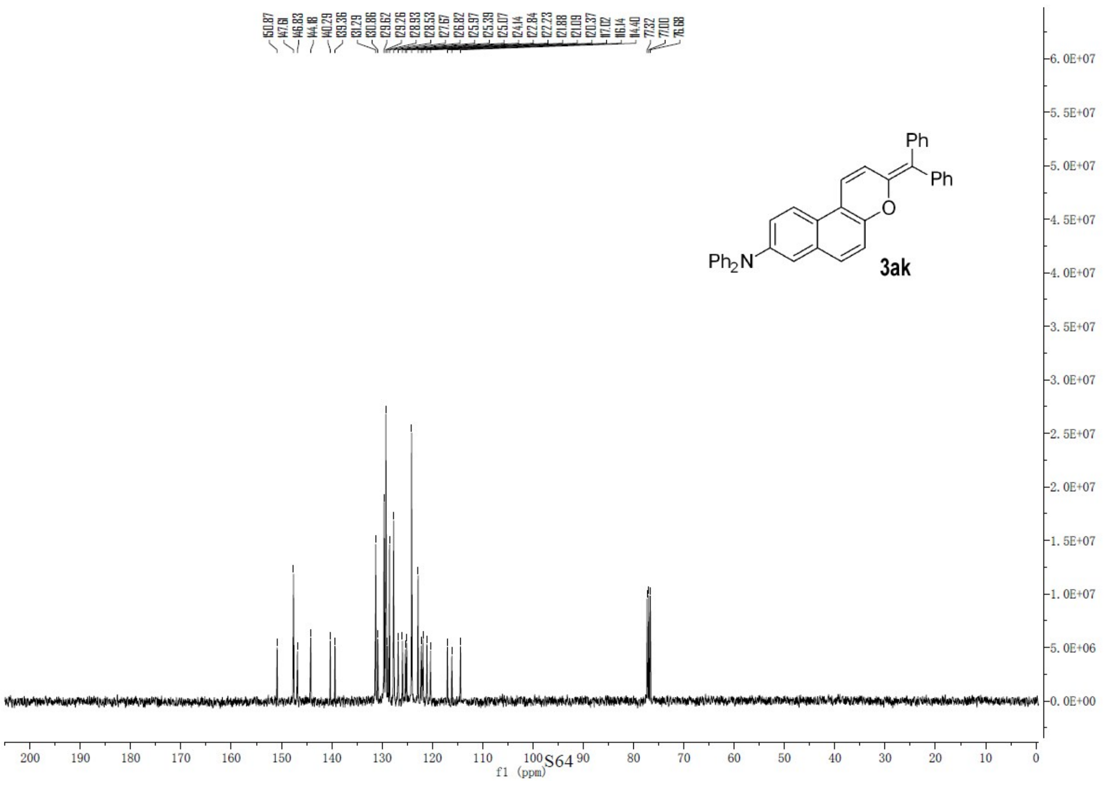
Ph
Ph
O
Ph_2N
3ak
f1 (ppm)
S64

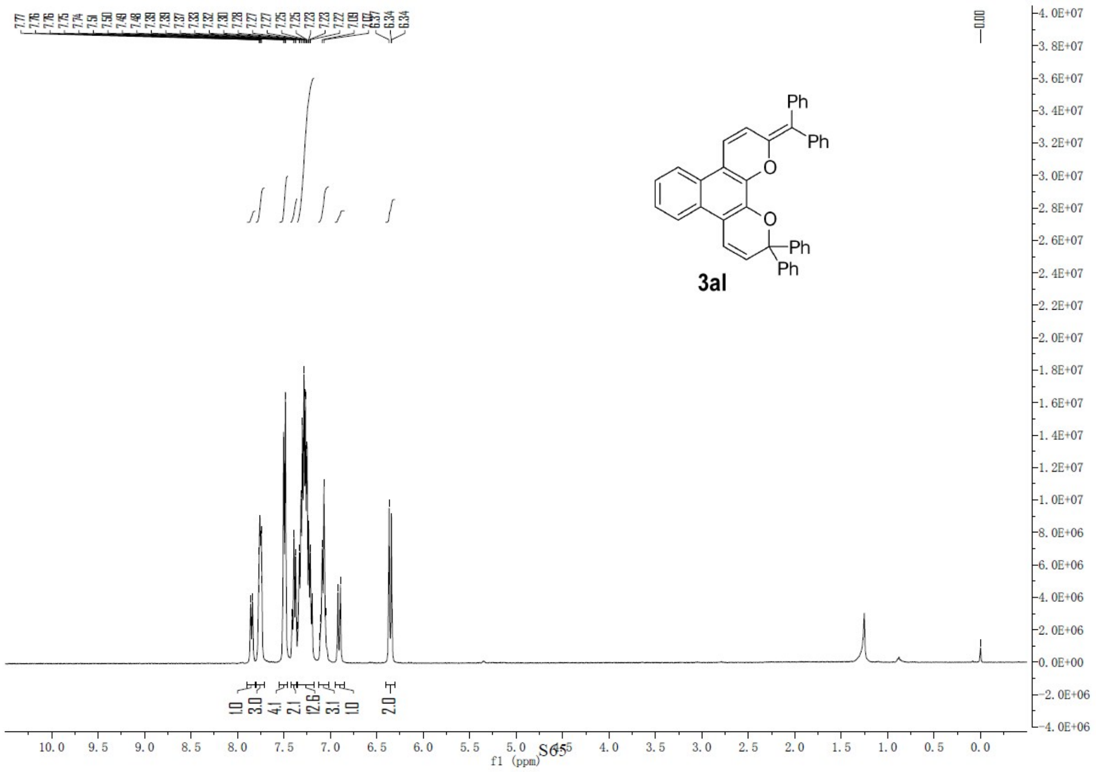
3al
f1 (ppm)

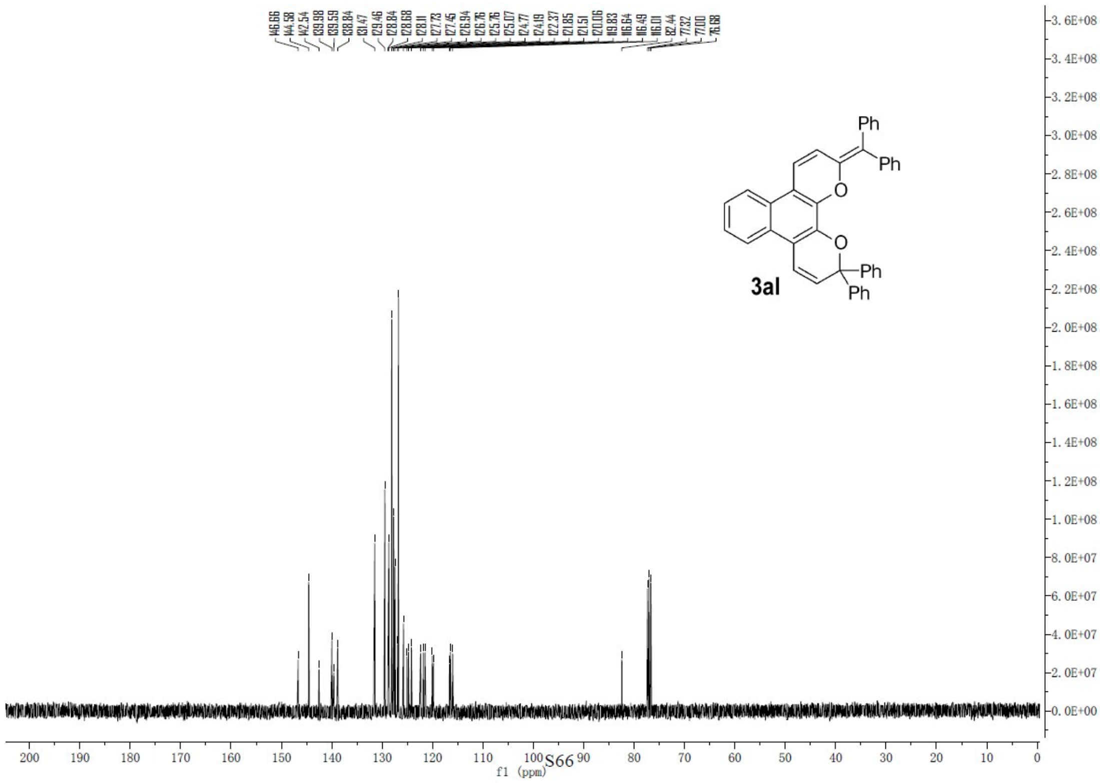
3al
f1 (ppm)

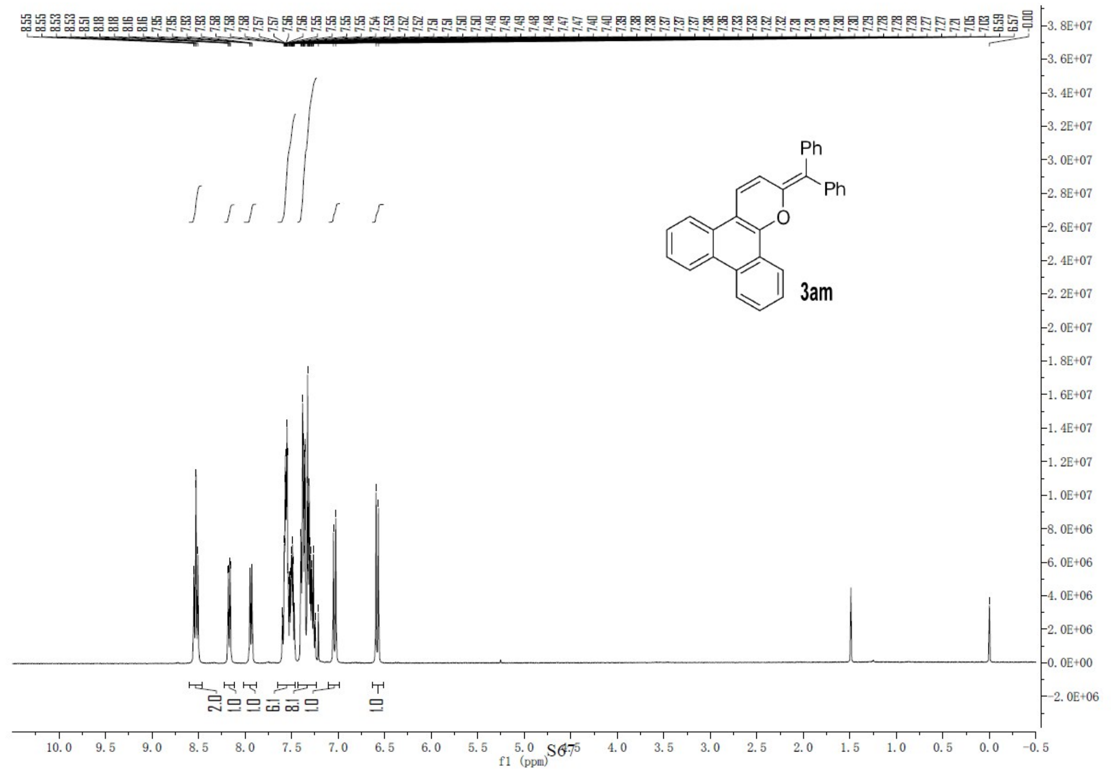
Ph
Ph
O
3am
f1 (ppm)
S67

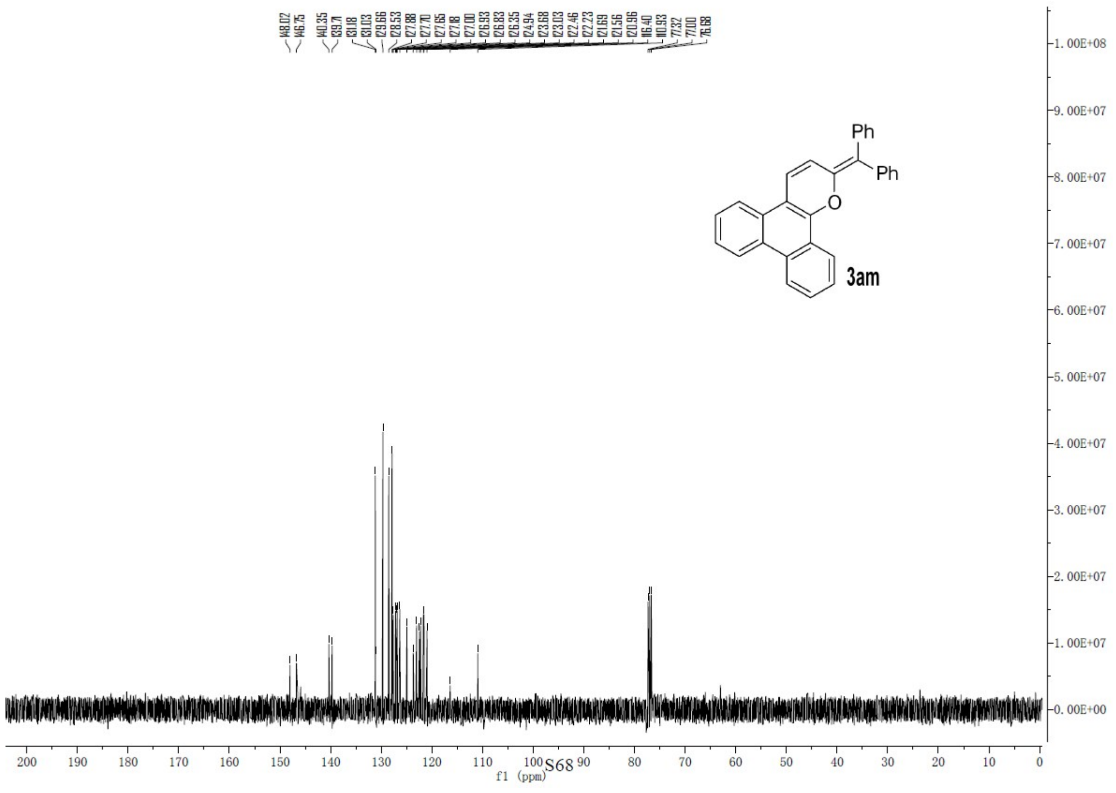
Ph
Ph
O
3am
f1 (ppm)
S68

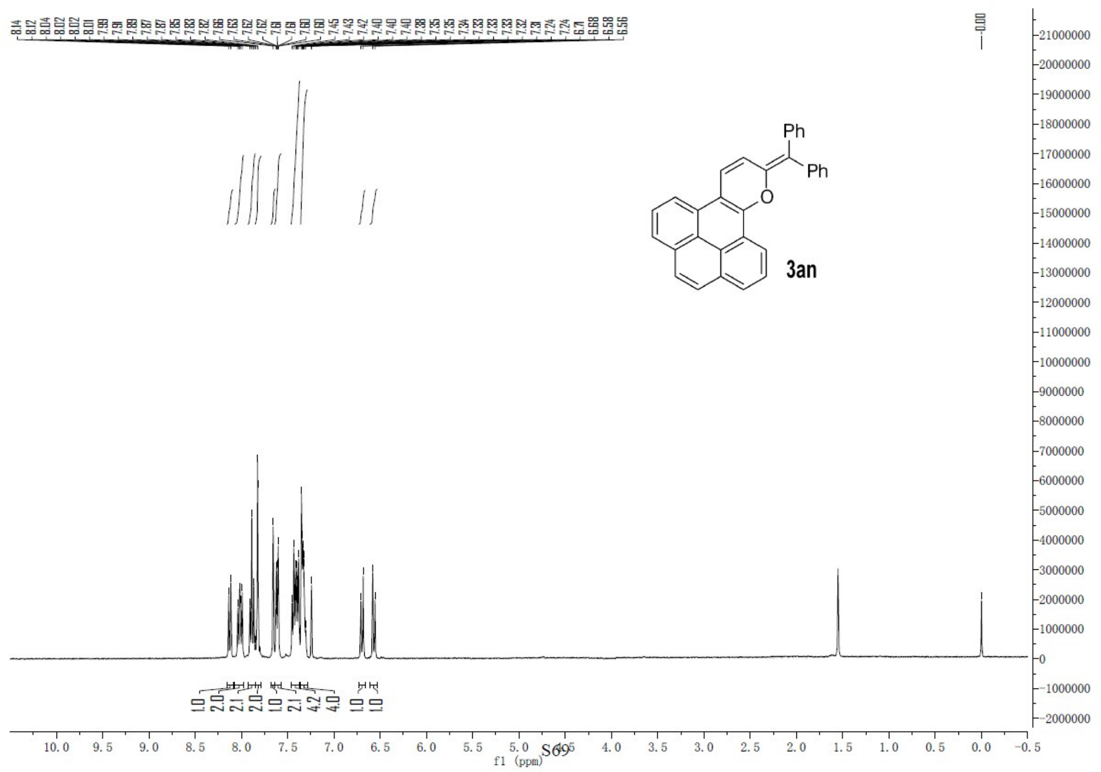

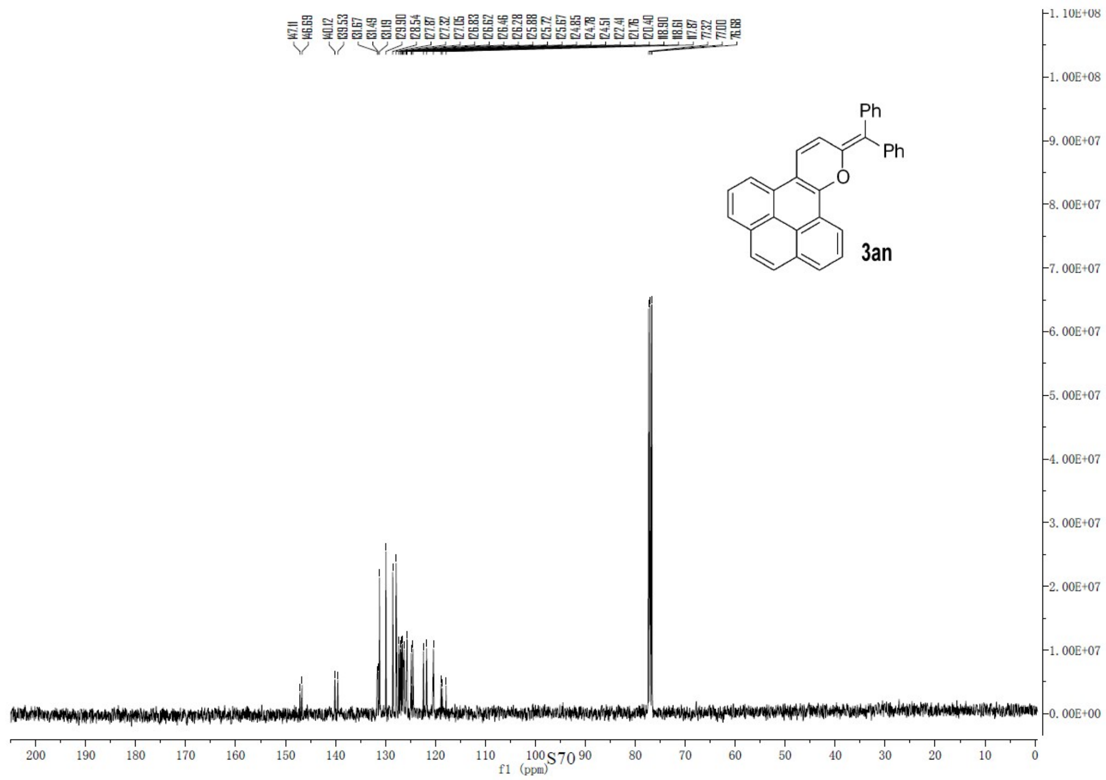

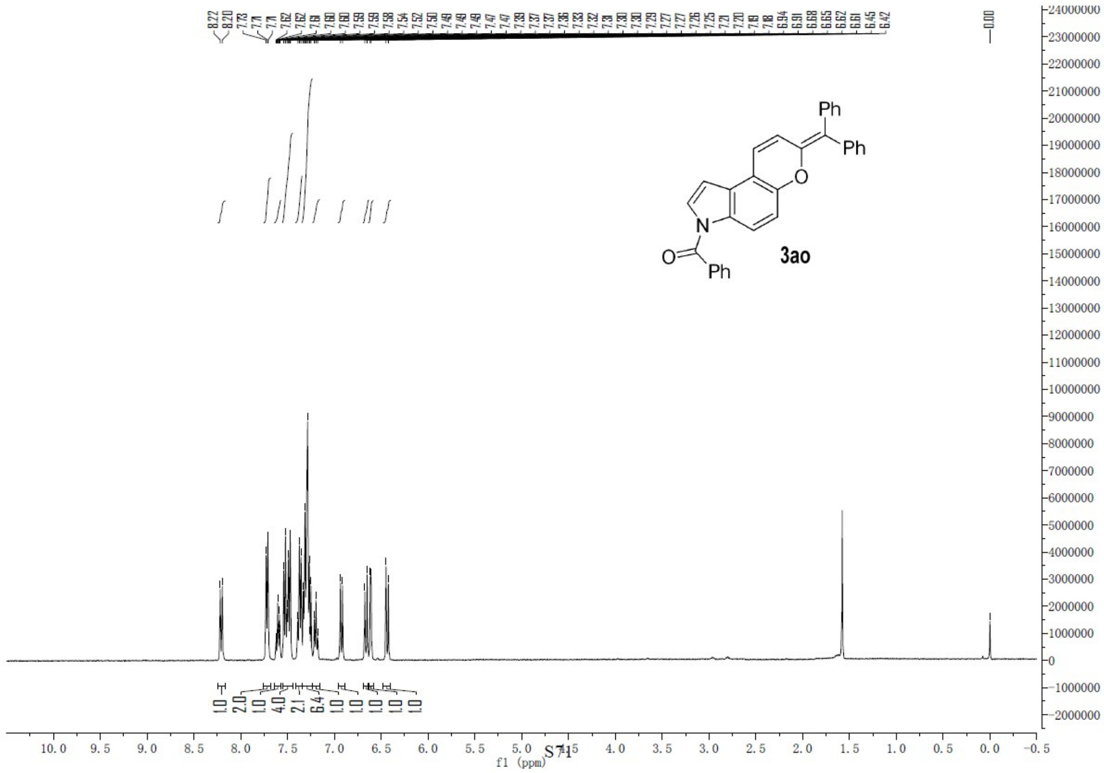
Ph
Ph
O
N
O
Ph
3ao
f1 (ppm)
S71

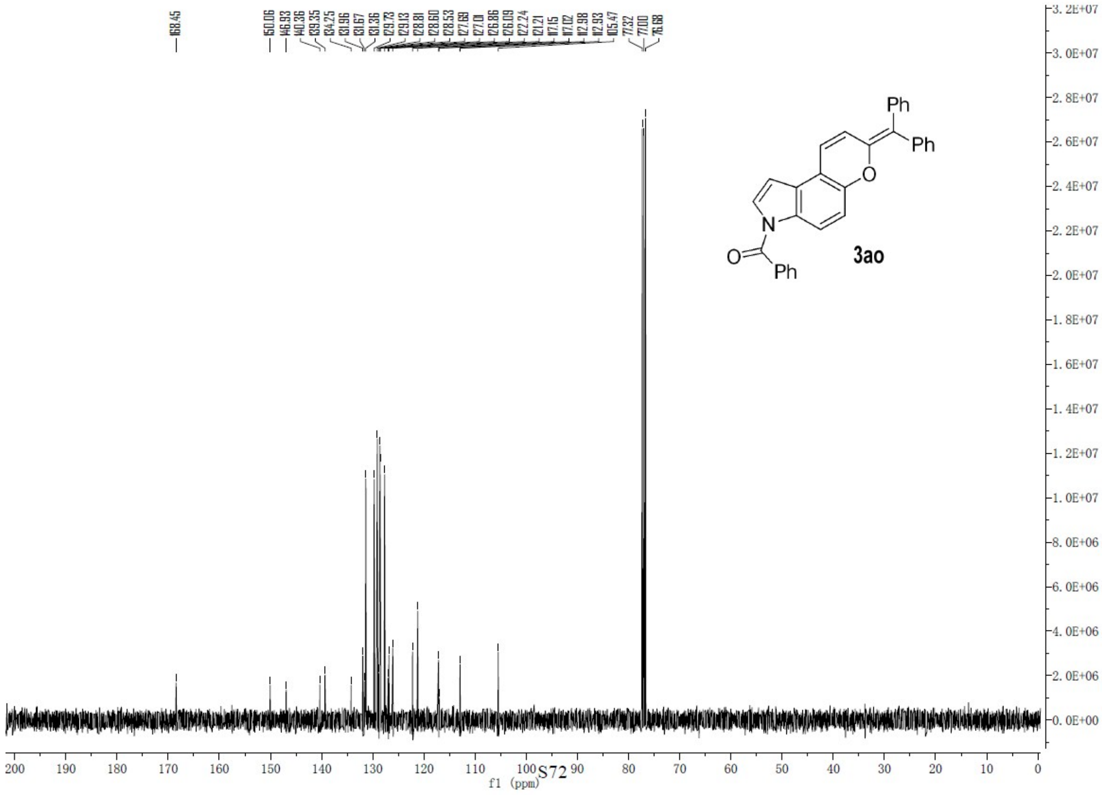
Ph
Ph
O
N
O
Ph
3ao
f1 (ppm)
S72

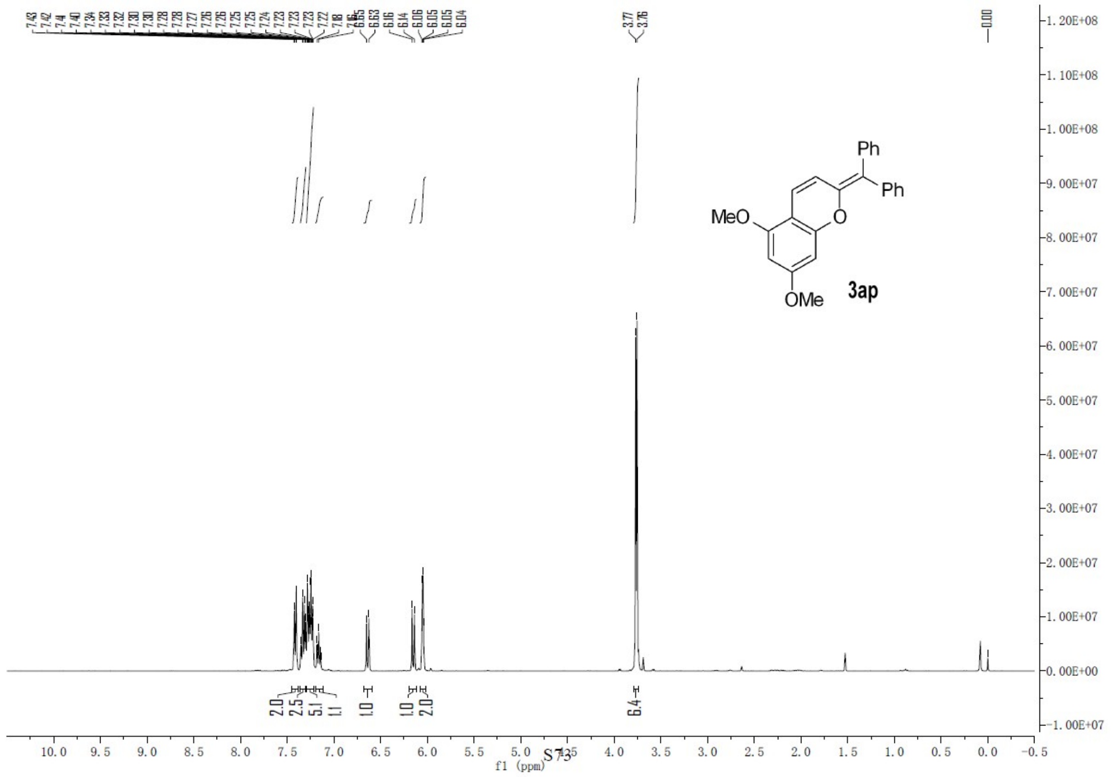

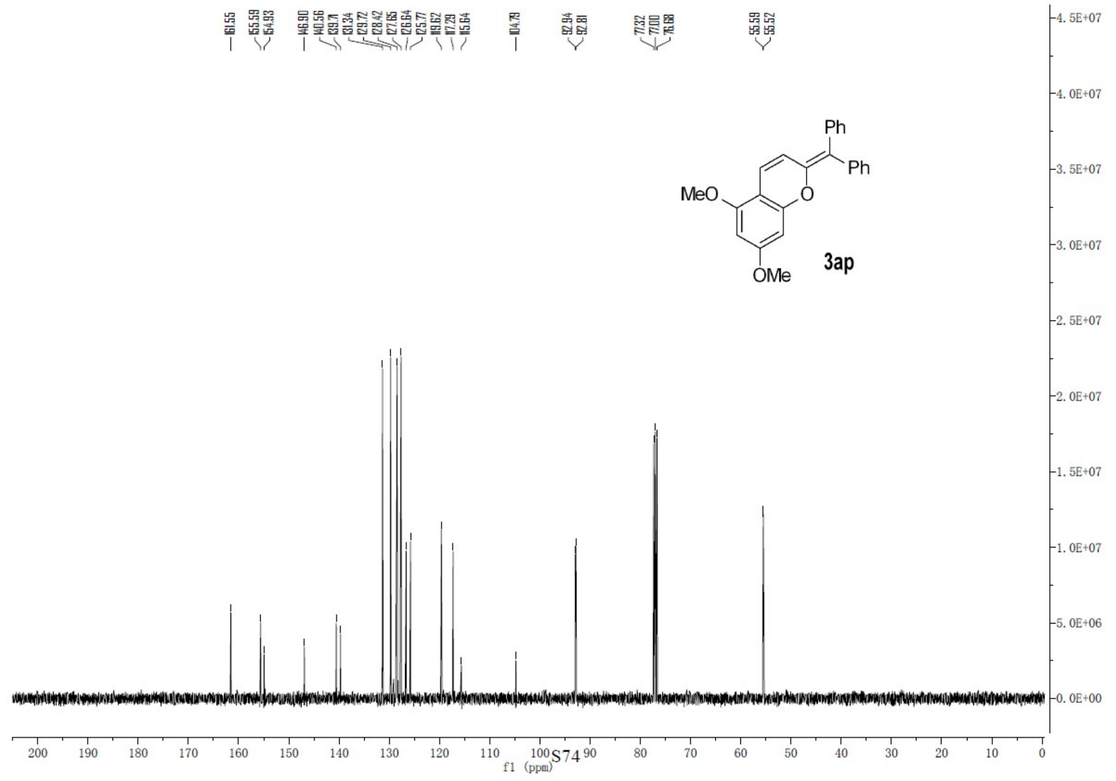

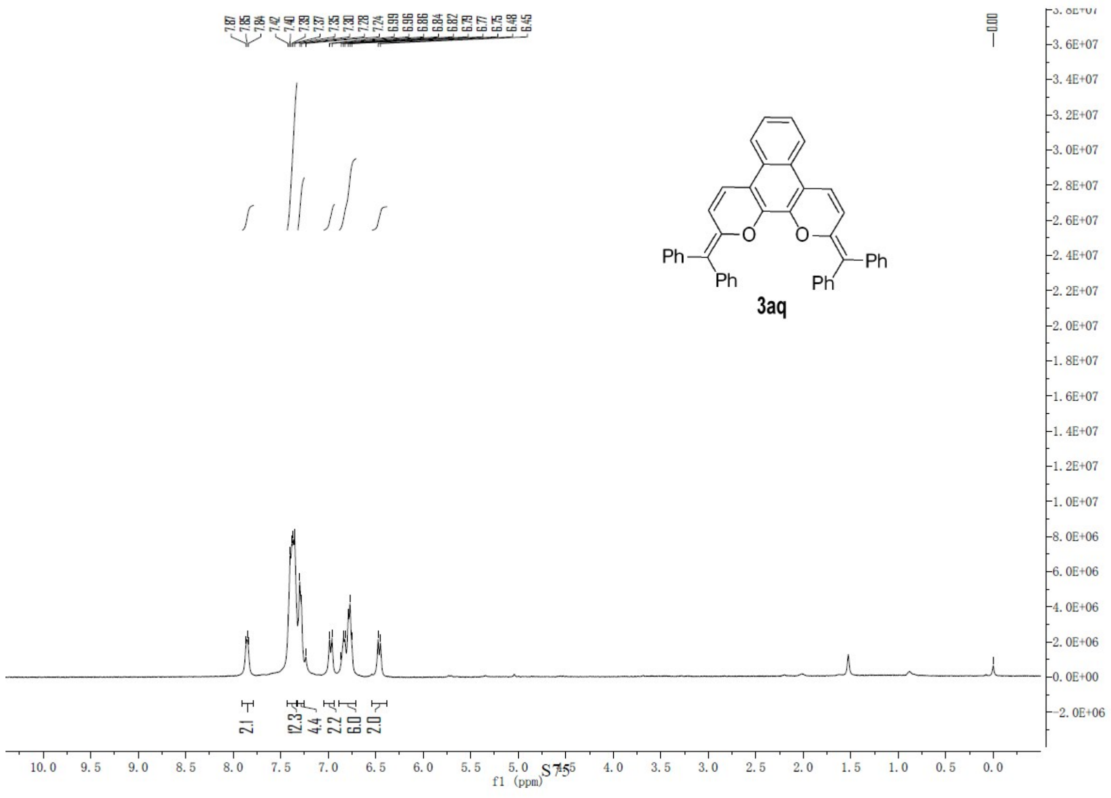

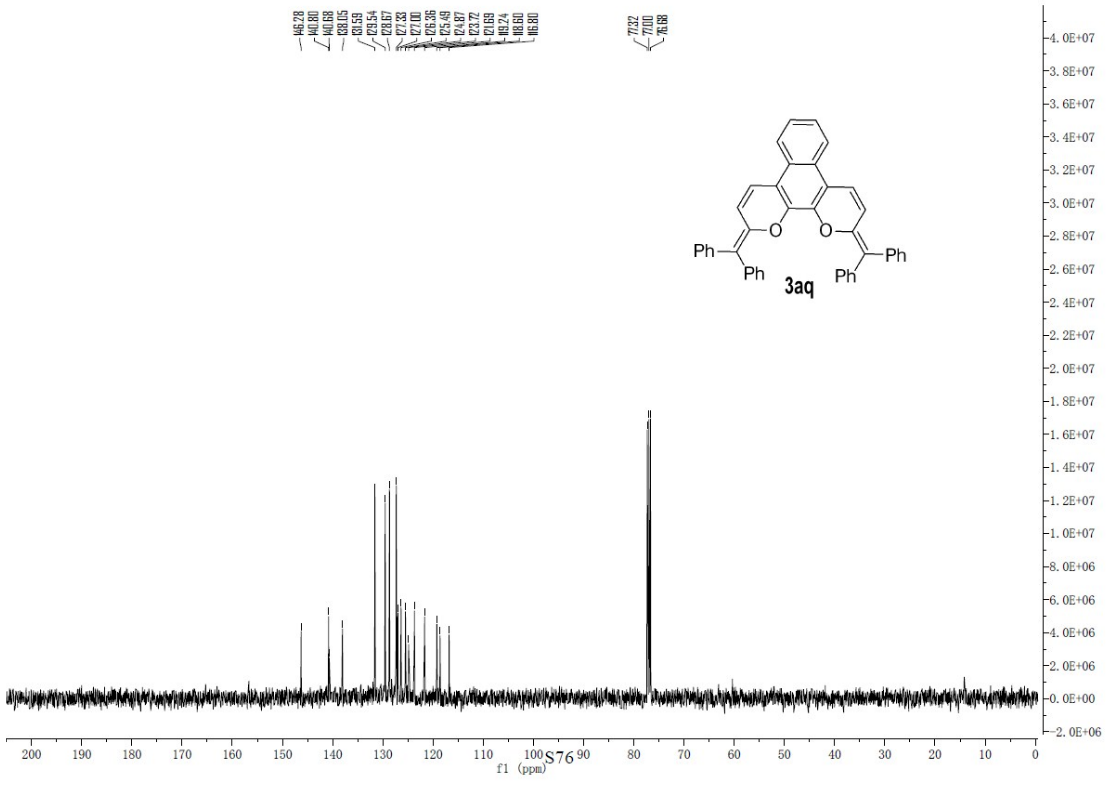

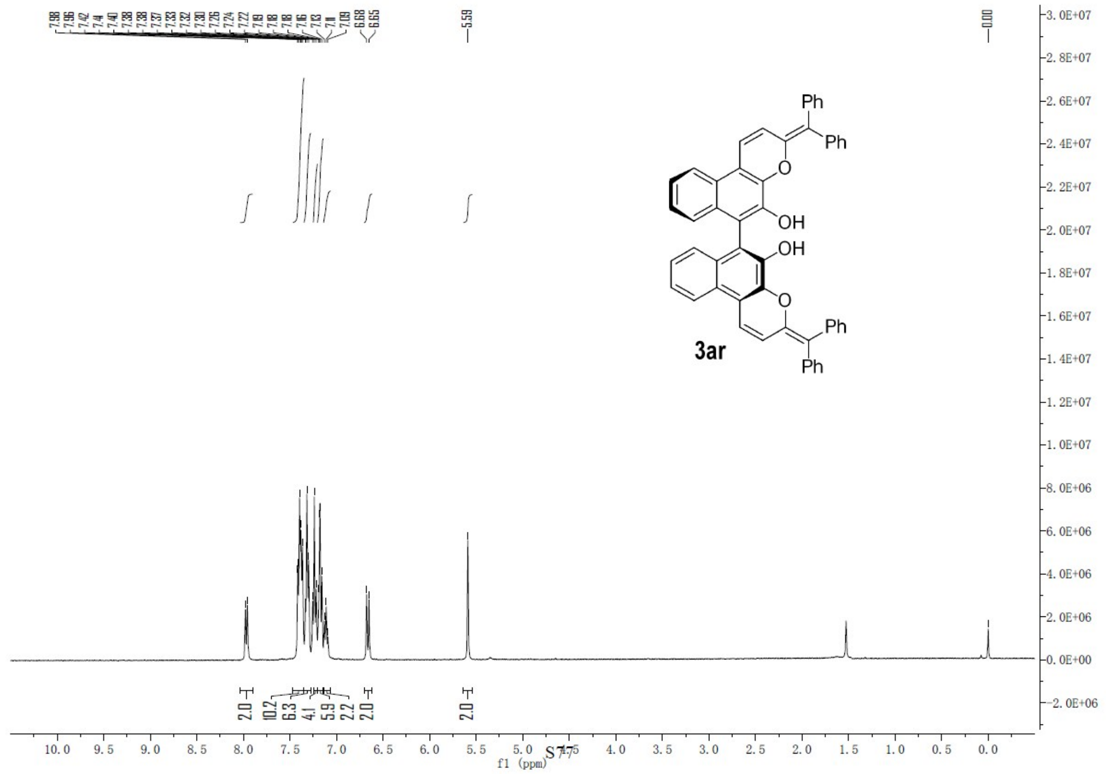
5.59
0.00
Ph
Ph
O
OH
OH
O
Ph
3ar
Ph
2.0
10.2
6.3
4.1
5.9
2.2
2.0
2.0
f1 (ppm)

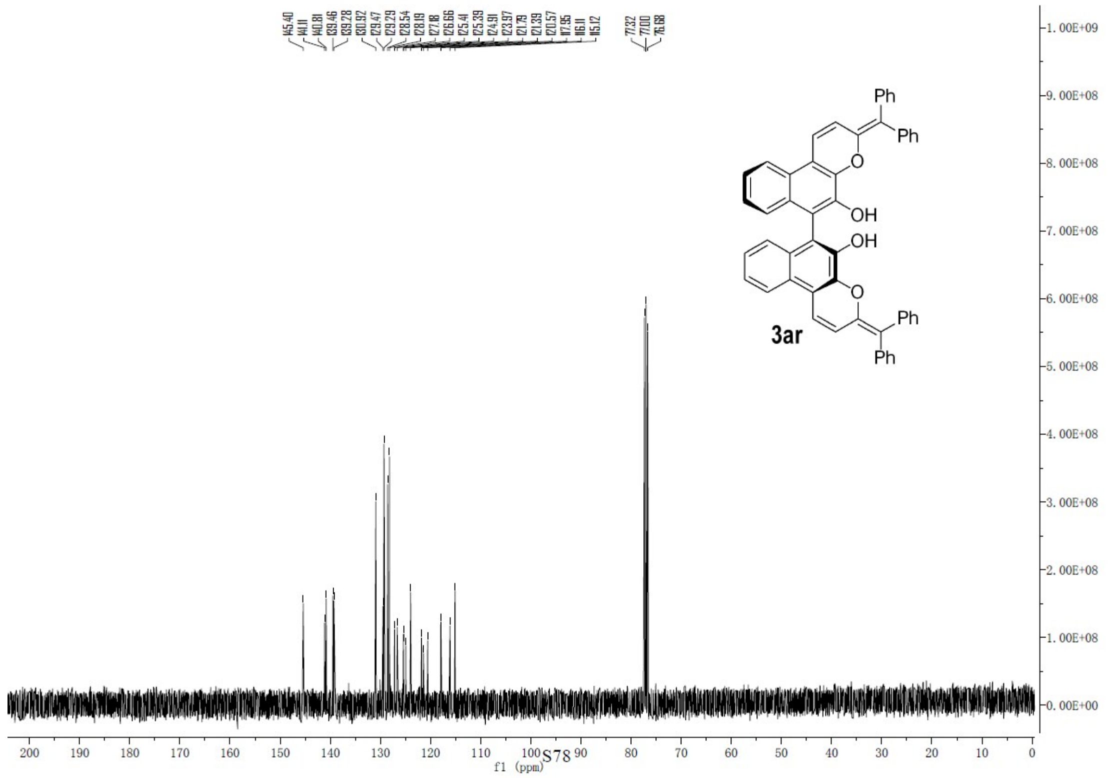
Ph
Ph
O
OH
OH
O
Ph
3ar
Ph
f1 (ppm)

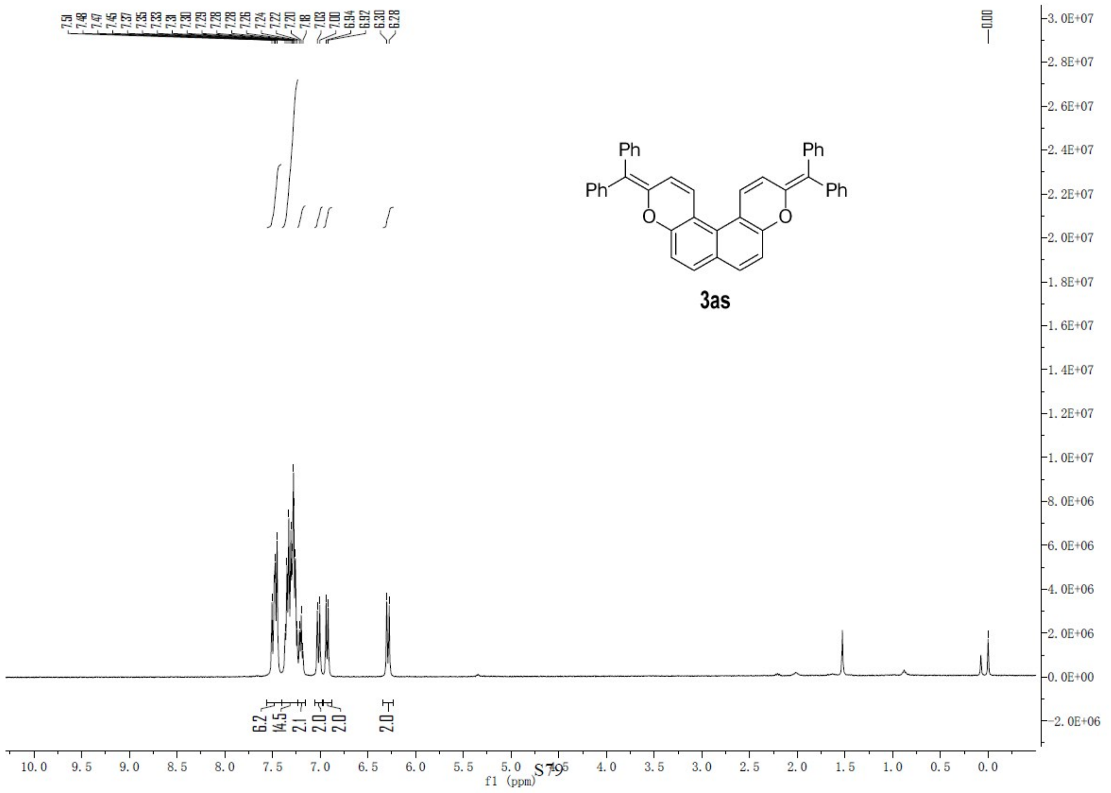
3as
S79
f1 (ppm)

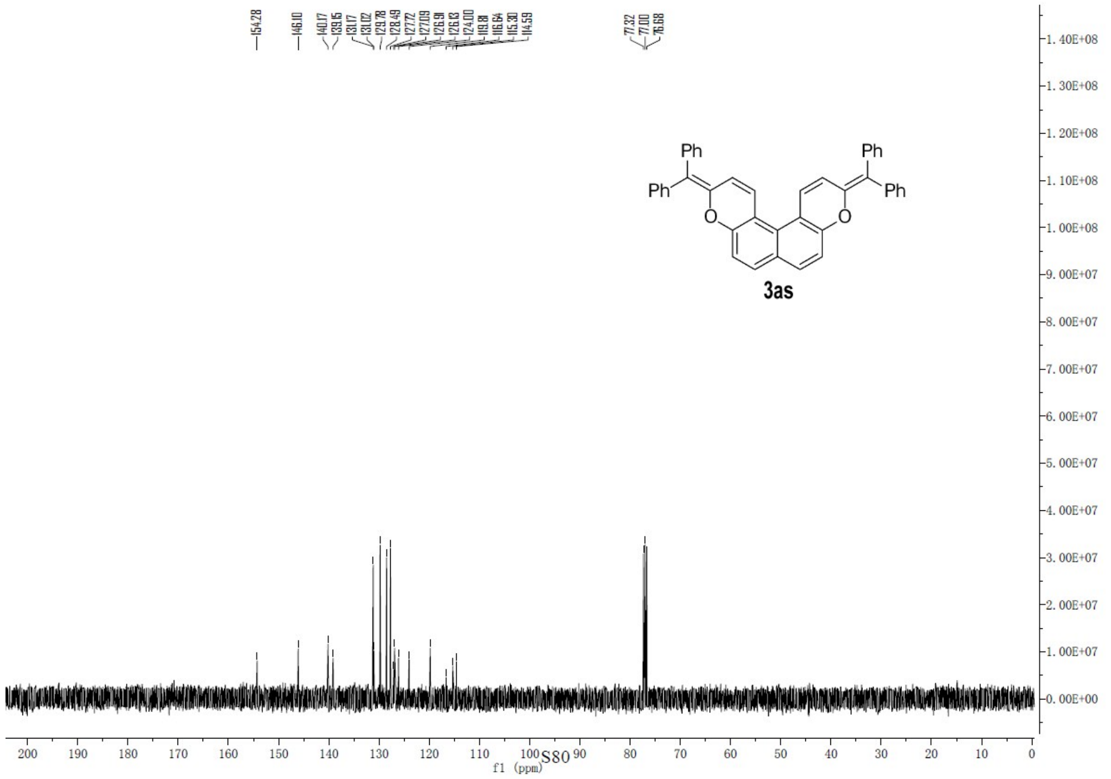
3as
S80
f1 (ppm)

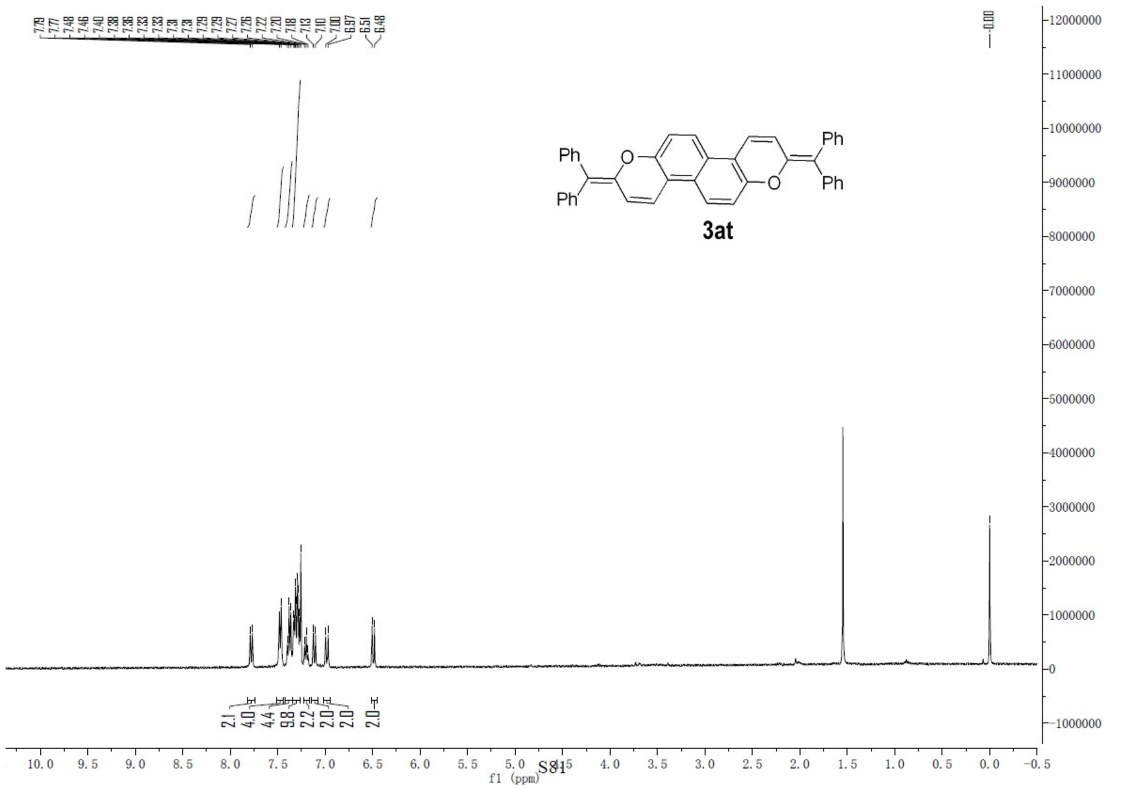

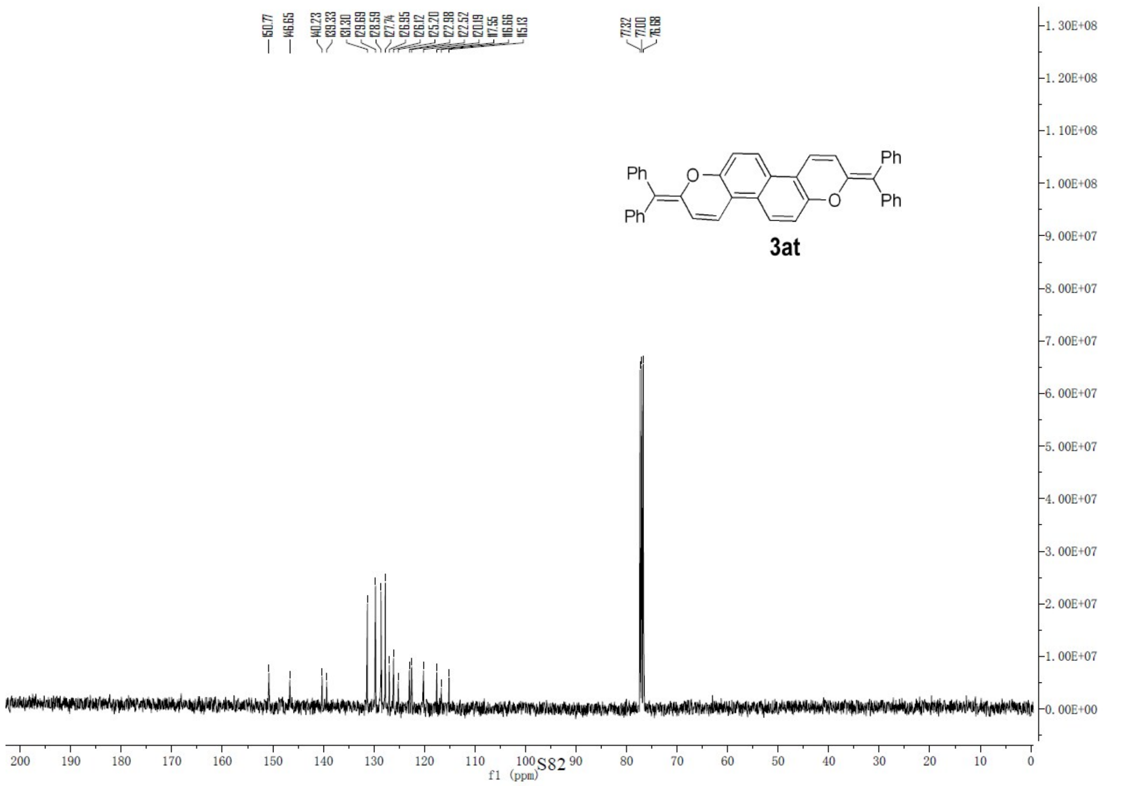

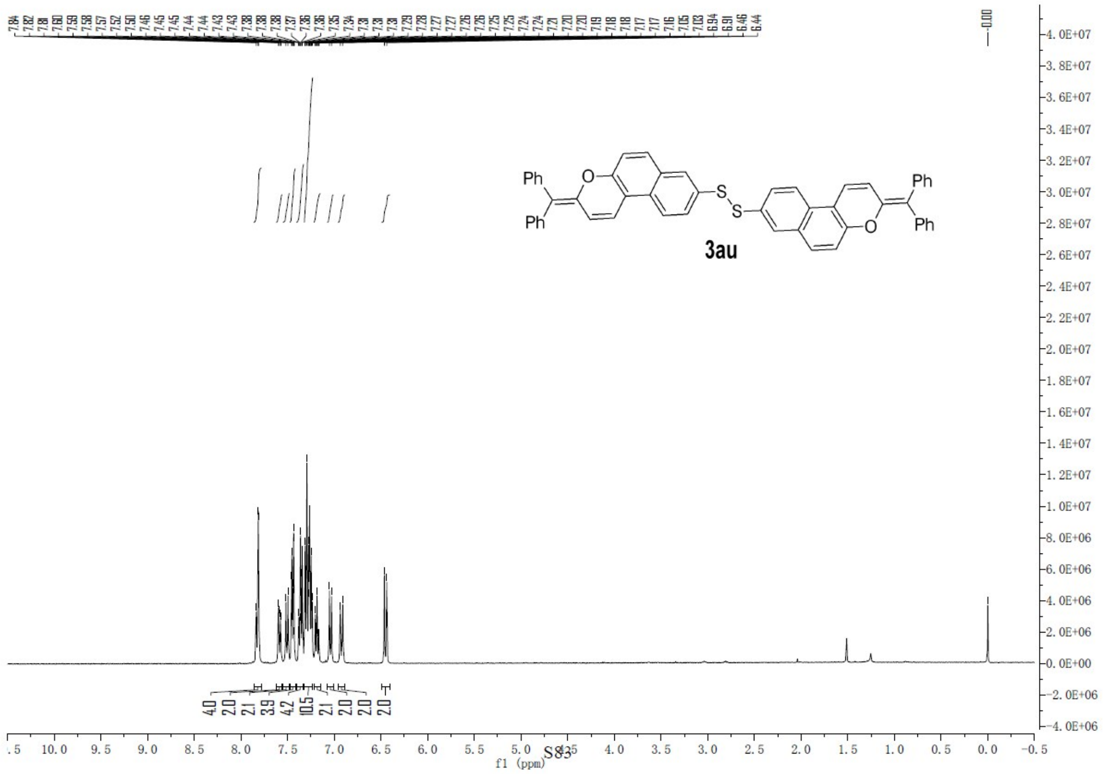
3au
S83
f1 (ppm)

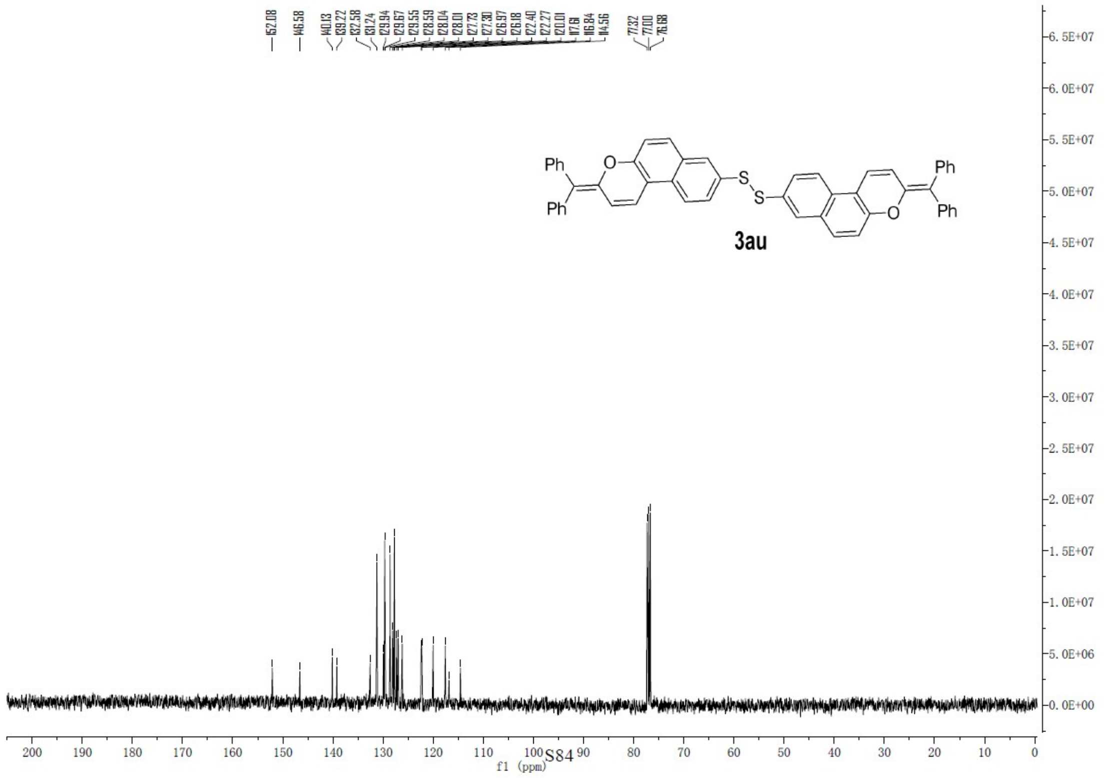
3au
S84
f1 (ppm)

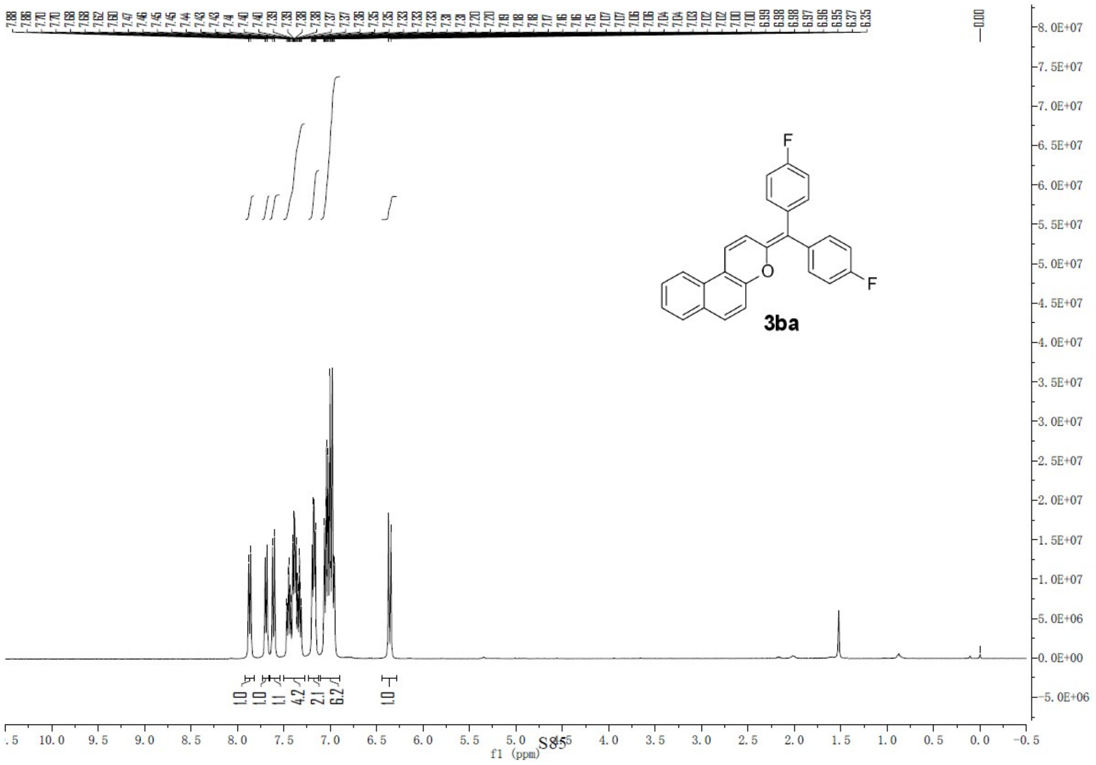

3ba
f1 (ppm)

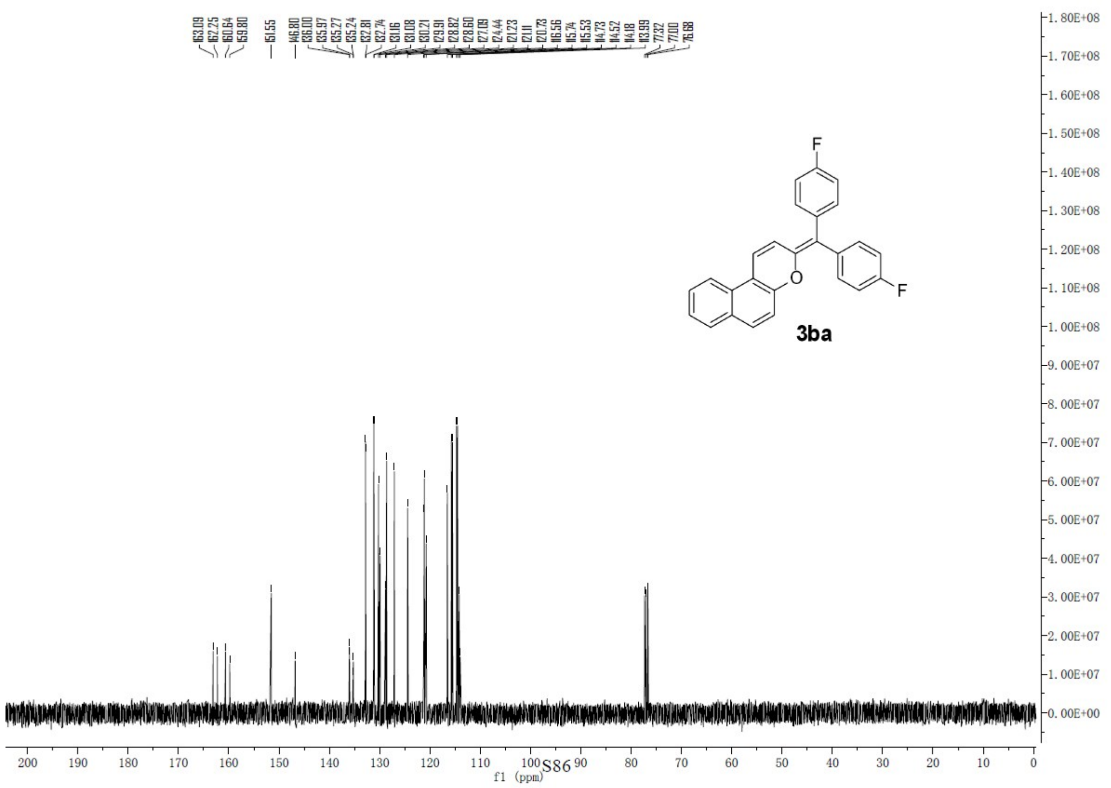

3ba
f1 (ppm)

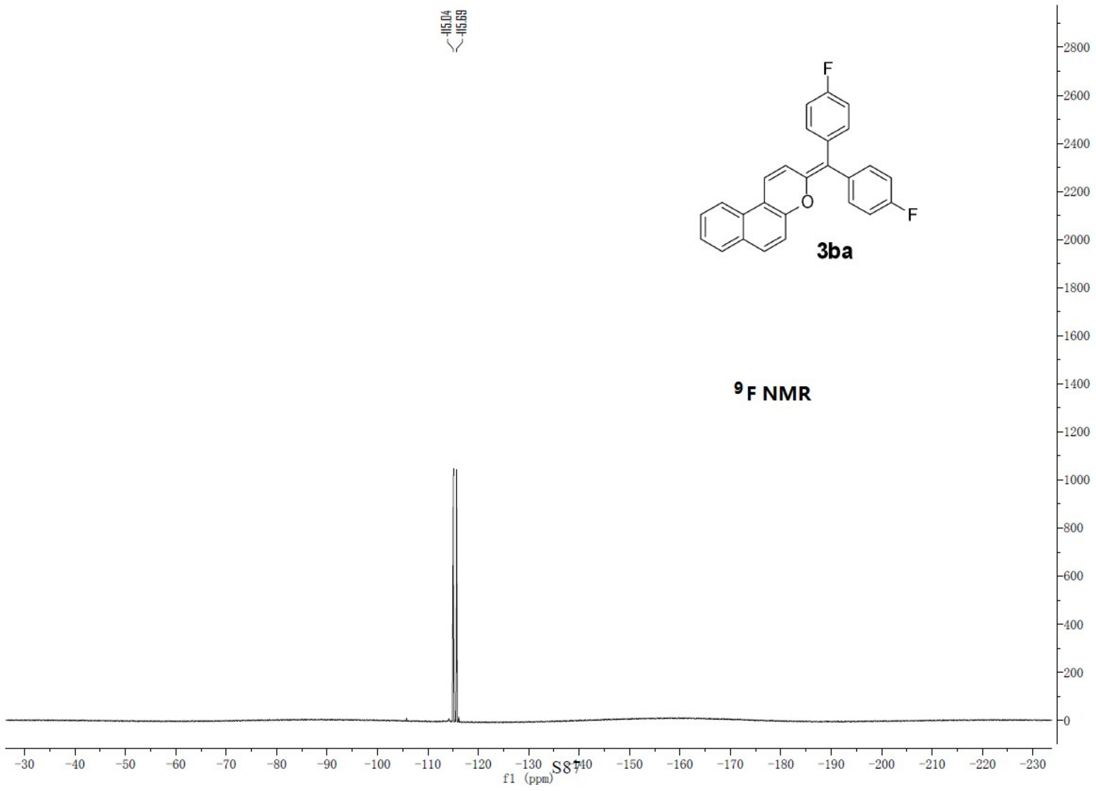

-115.04
-115.69
F
O
F
3ba
9F NMR
f1 (ppm)

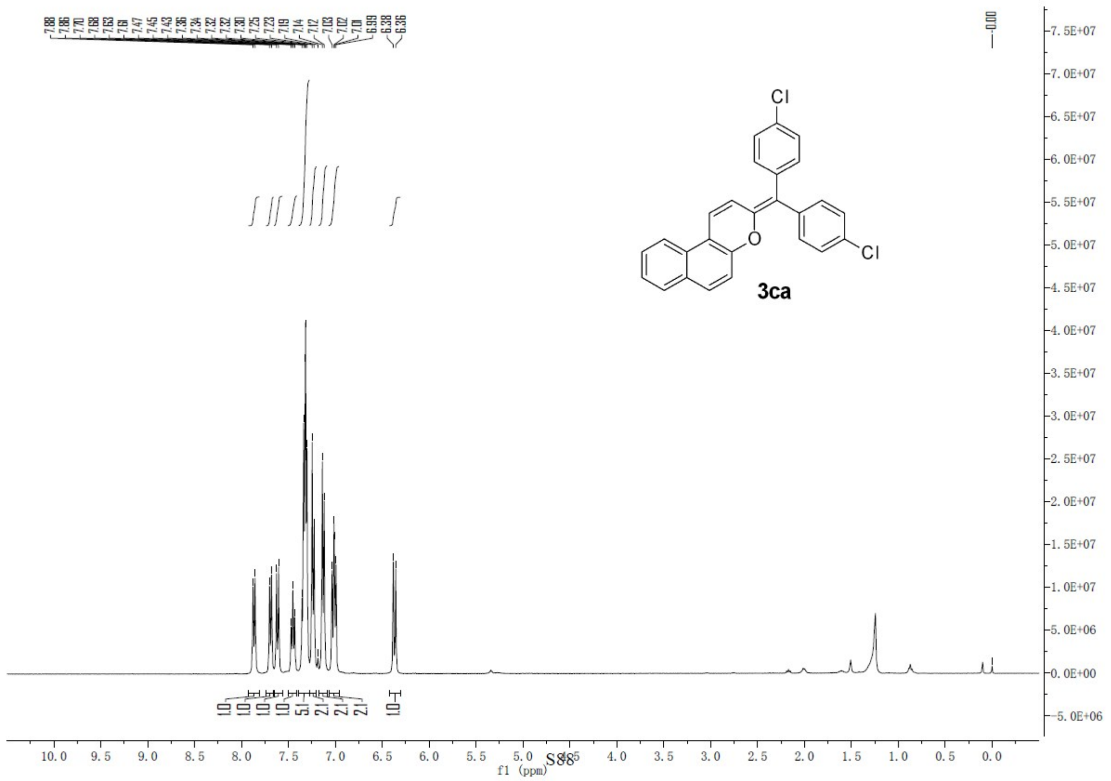
Cl
O
Cl
3ca
f1 (ppm)
S88

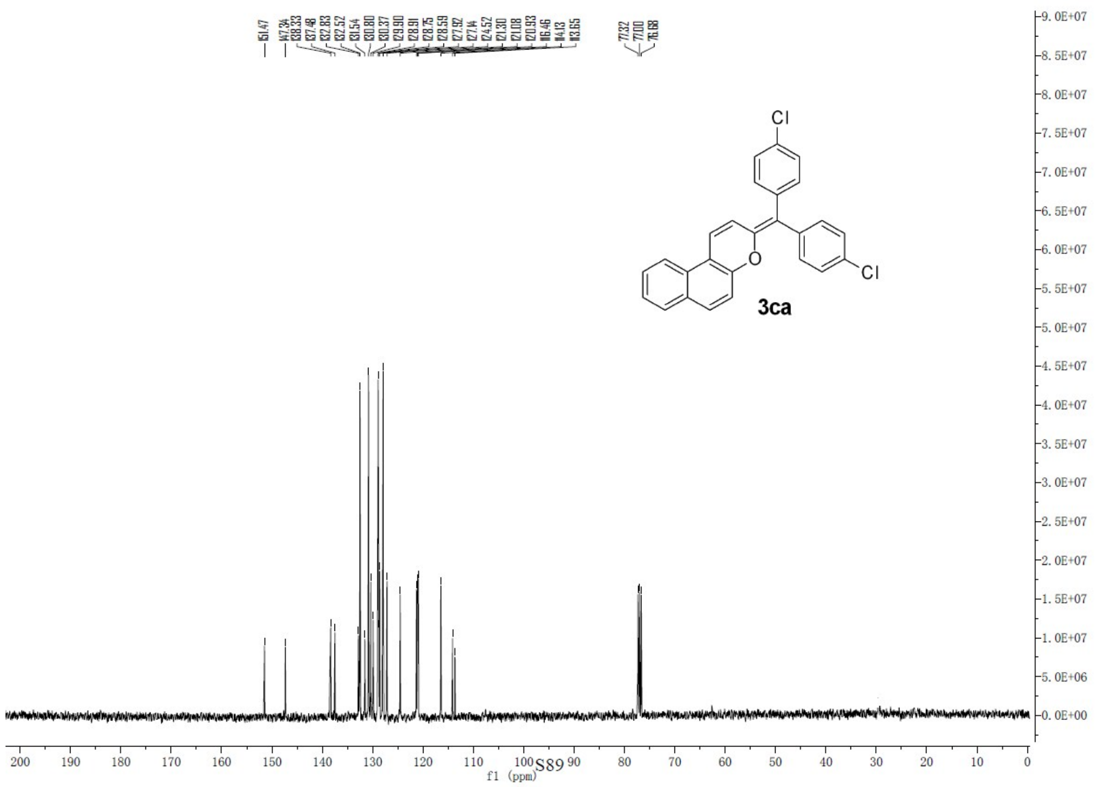
Cl
O
Cl
3ca
f1 (ppm)
S89

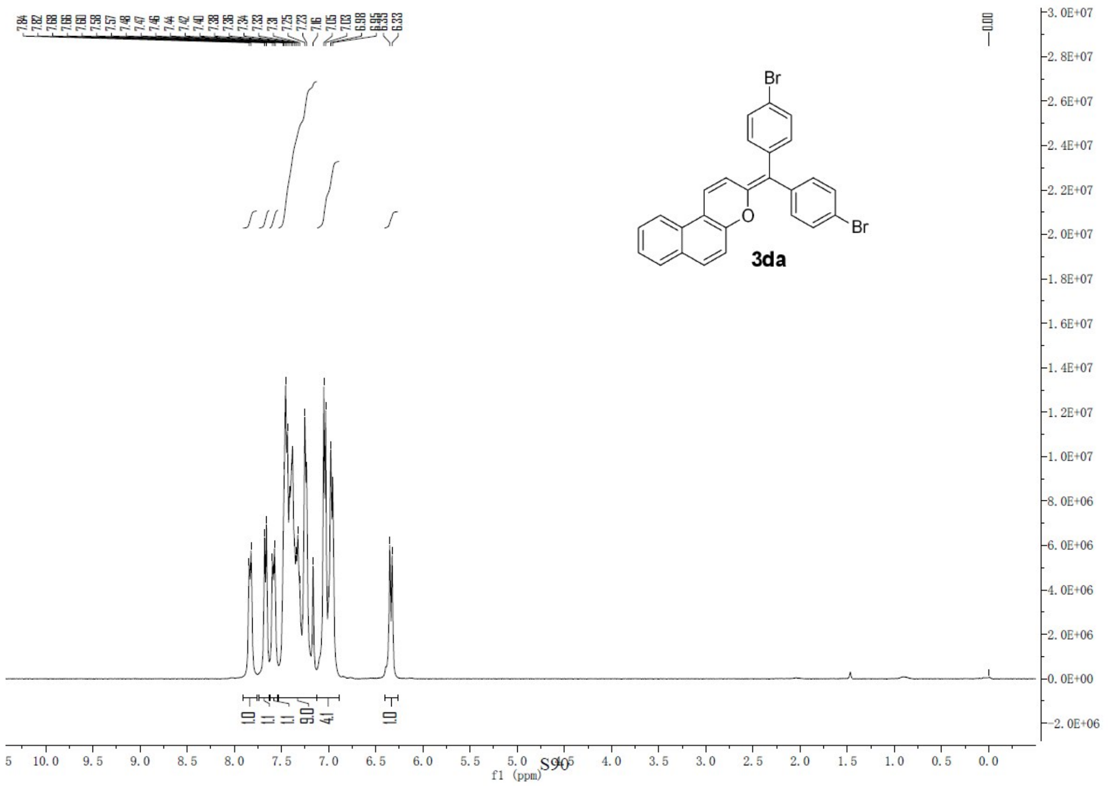
Br
O
Br
3da
S90
f1 (ppm)

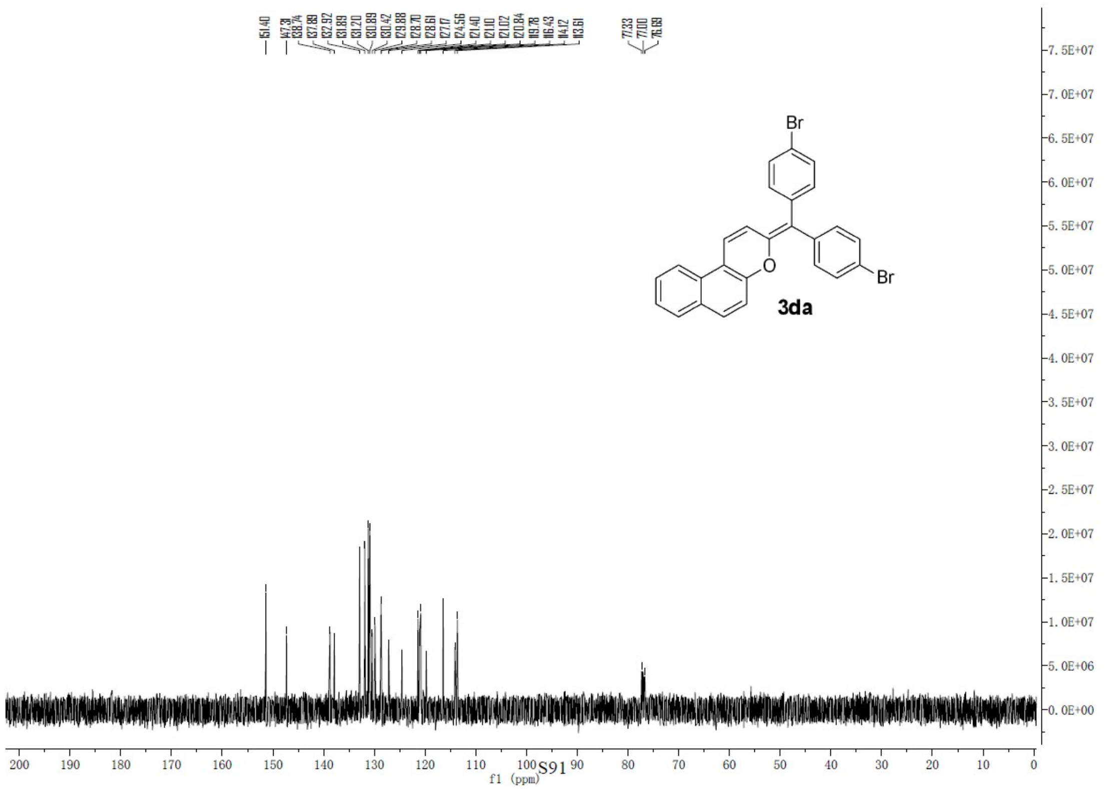
Br
O
Br
3da
S91
f1 (ppm)

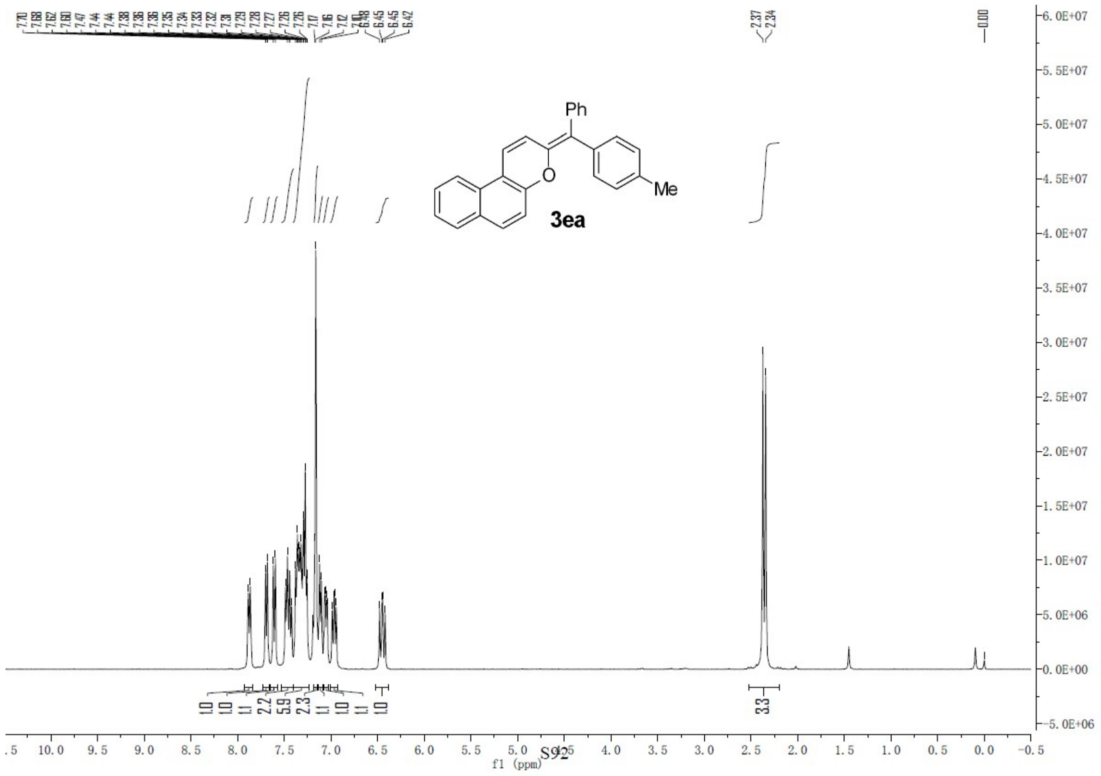
3ea
Ph
O
Me
f1 (ppm)
S92

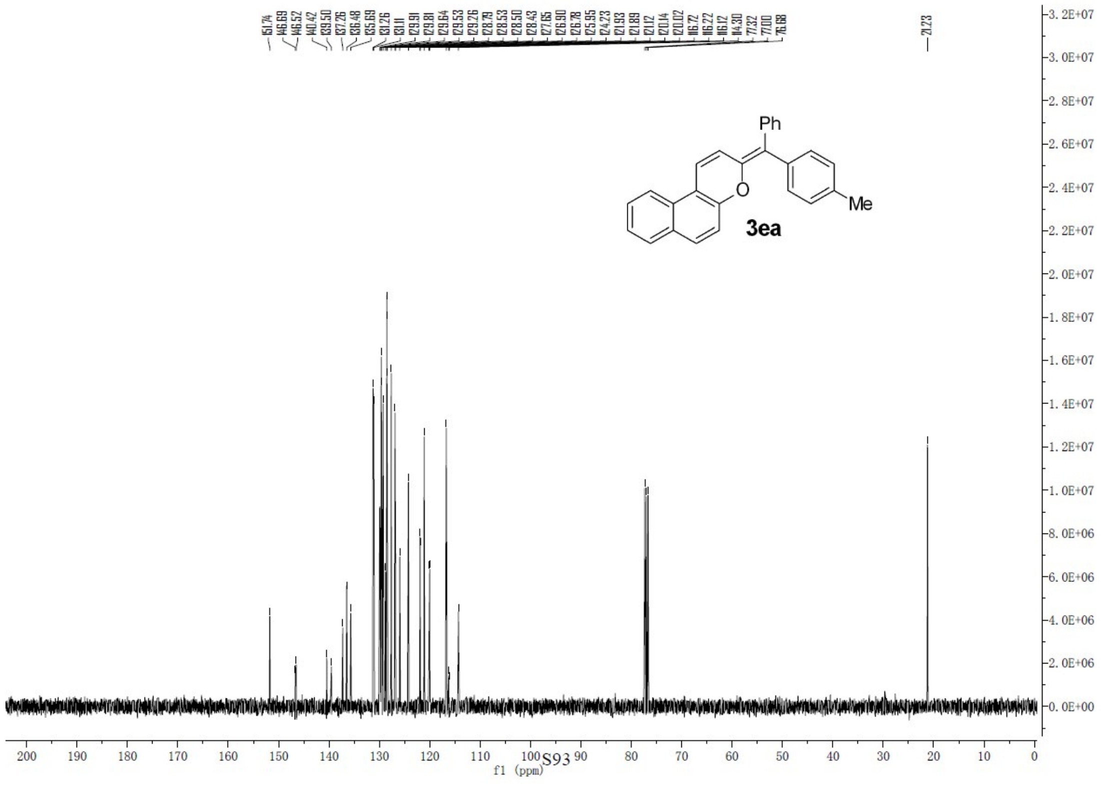
3ea
Ph
O
Me
f1 (ppm)
S93

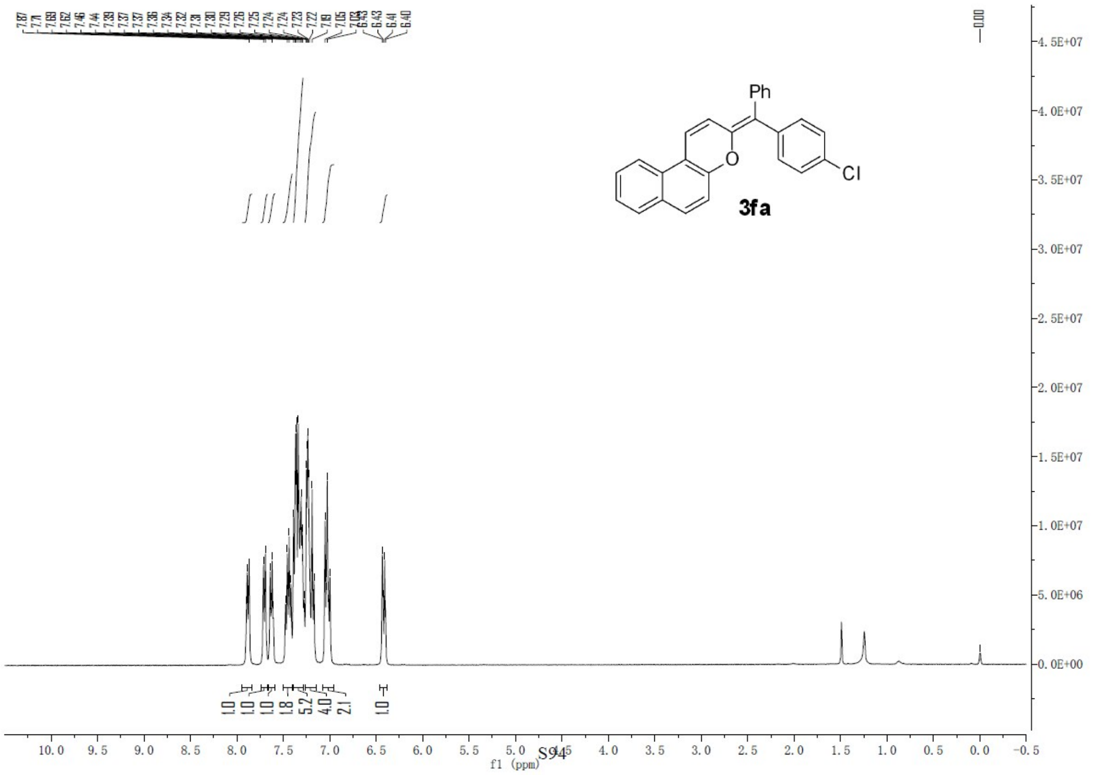
Ph
O
Cl
3fa
f1 (ppm)
S94

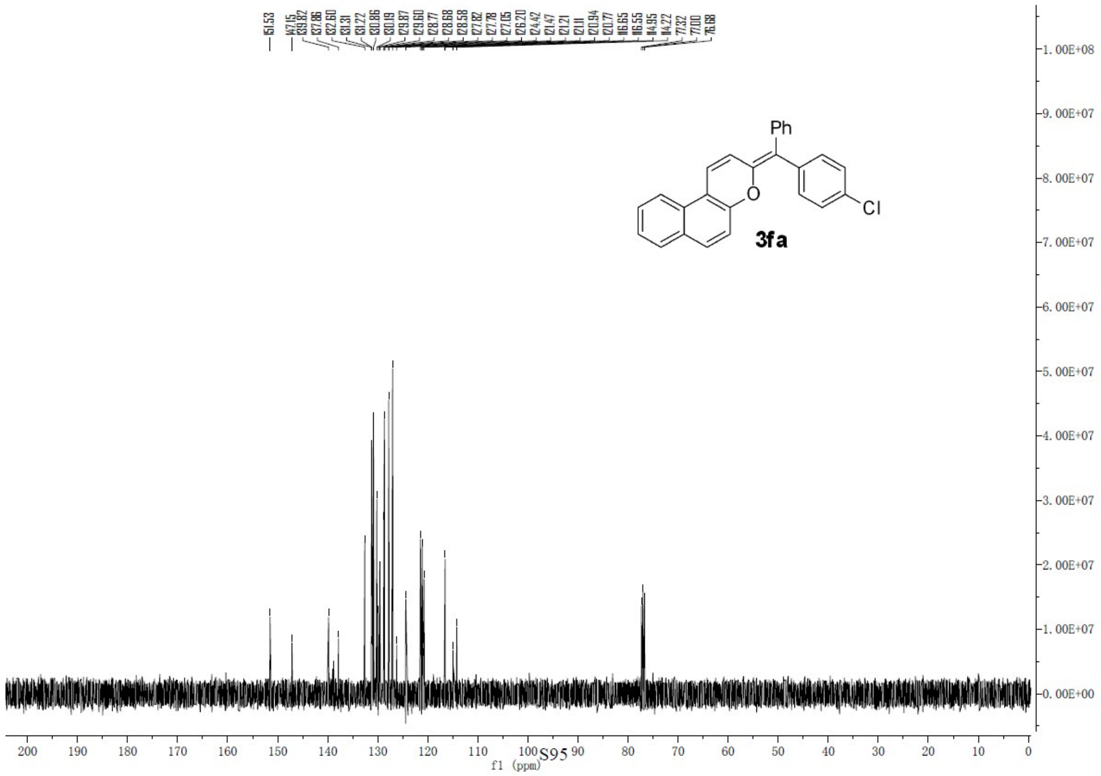
Ph
O
Cl
3fa
f1 (ppm)
S95

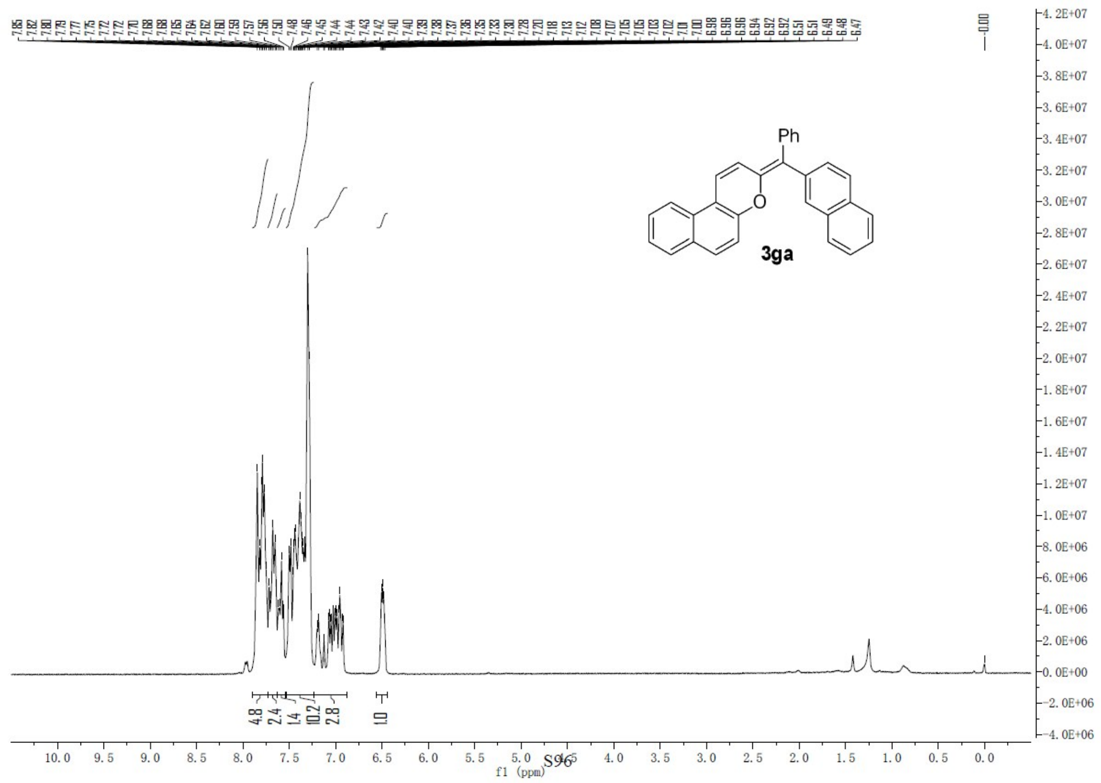
Ph
O
3ga
f1 (ppm)
S96

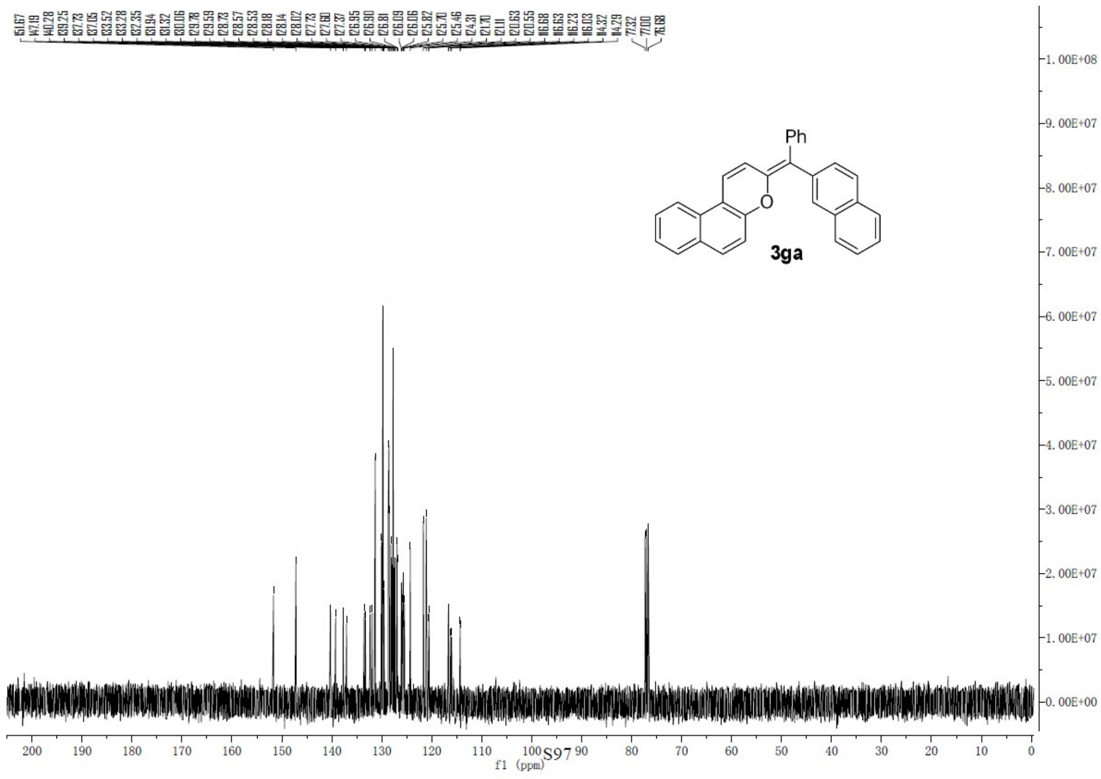
Ph
O
3ga
f1 (ppm)
S97

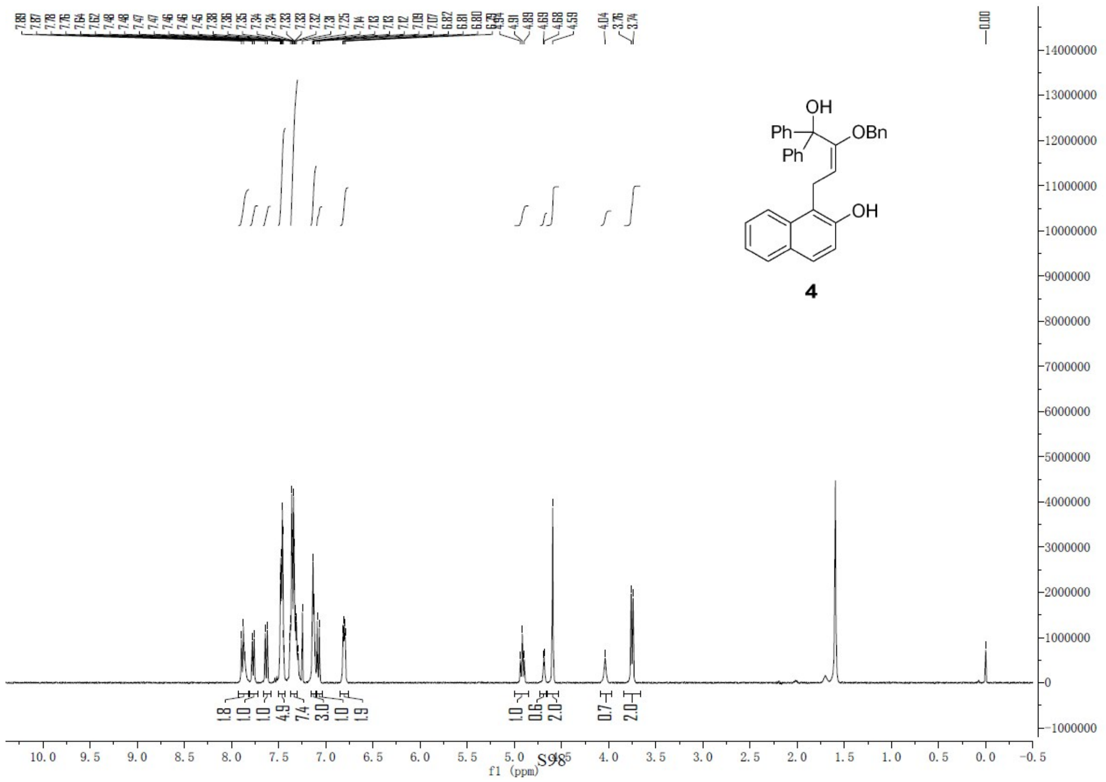

S98

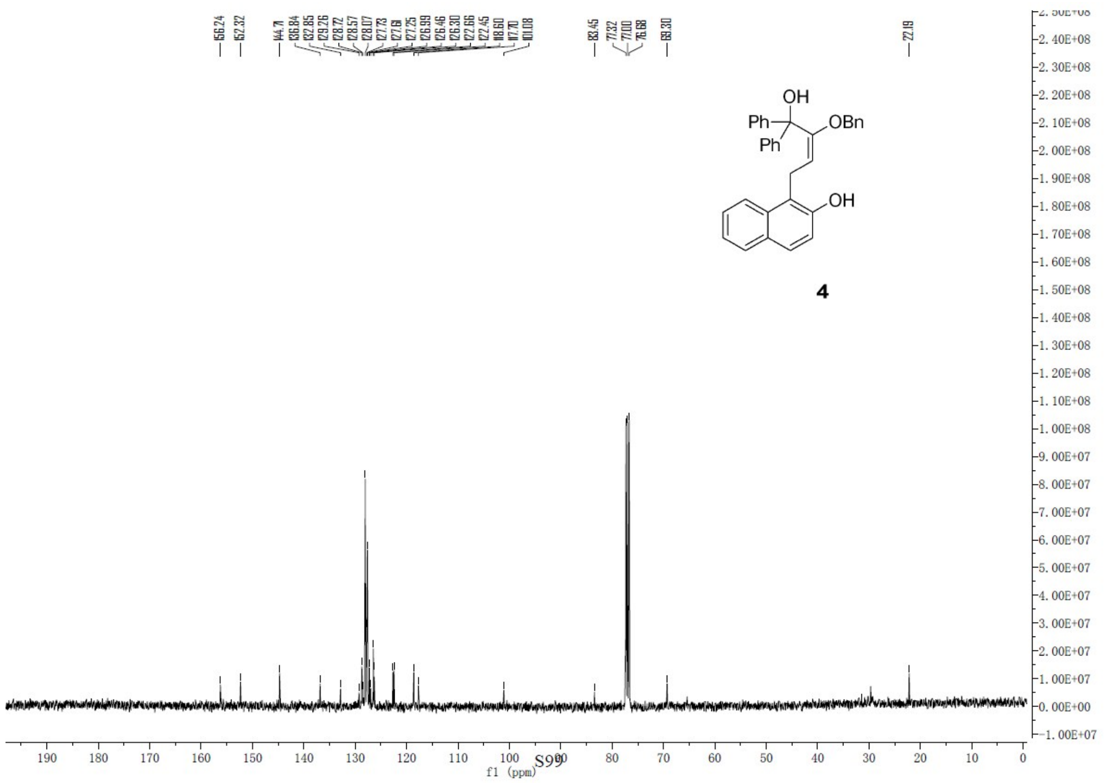

S99

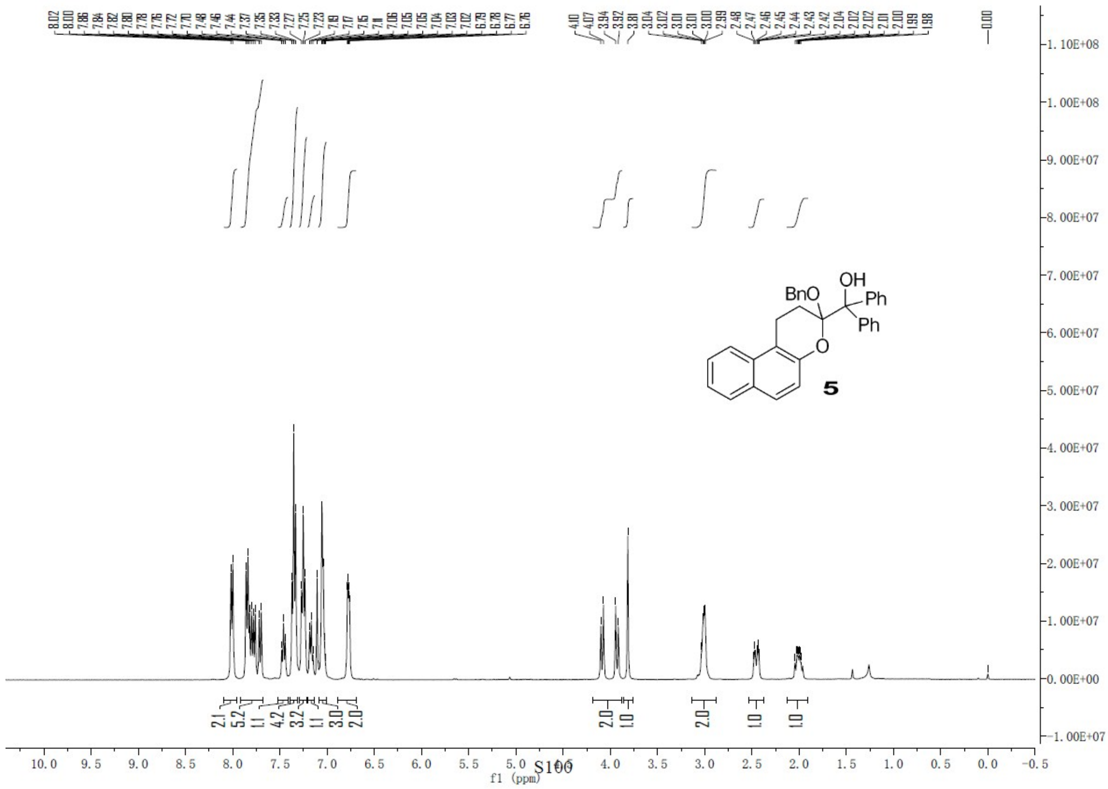

S100

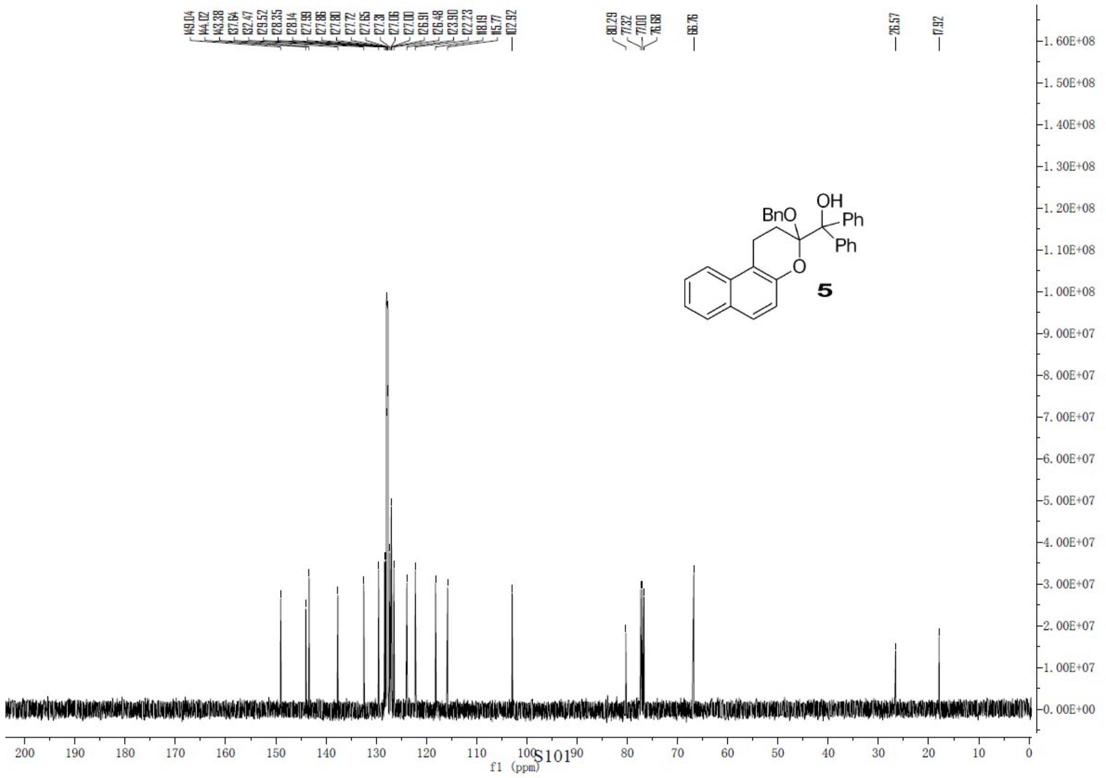

S101

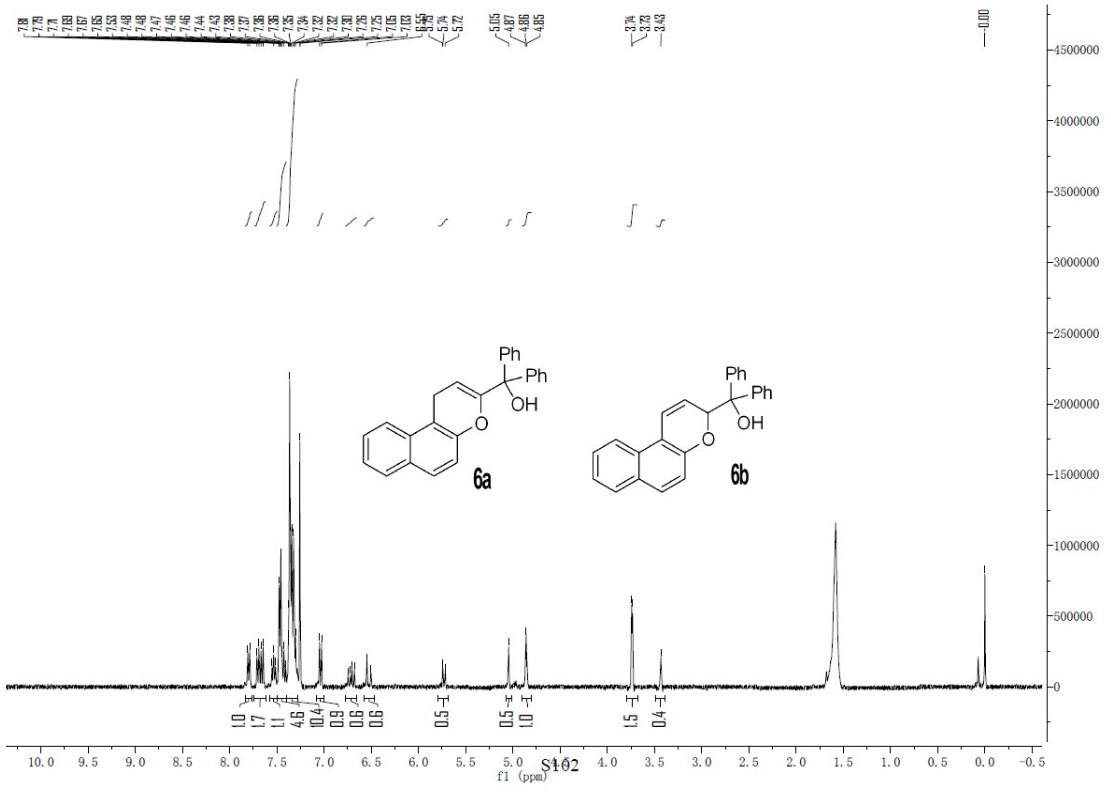

S102

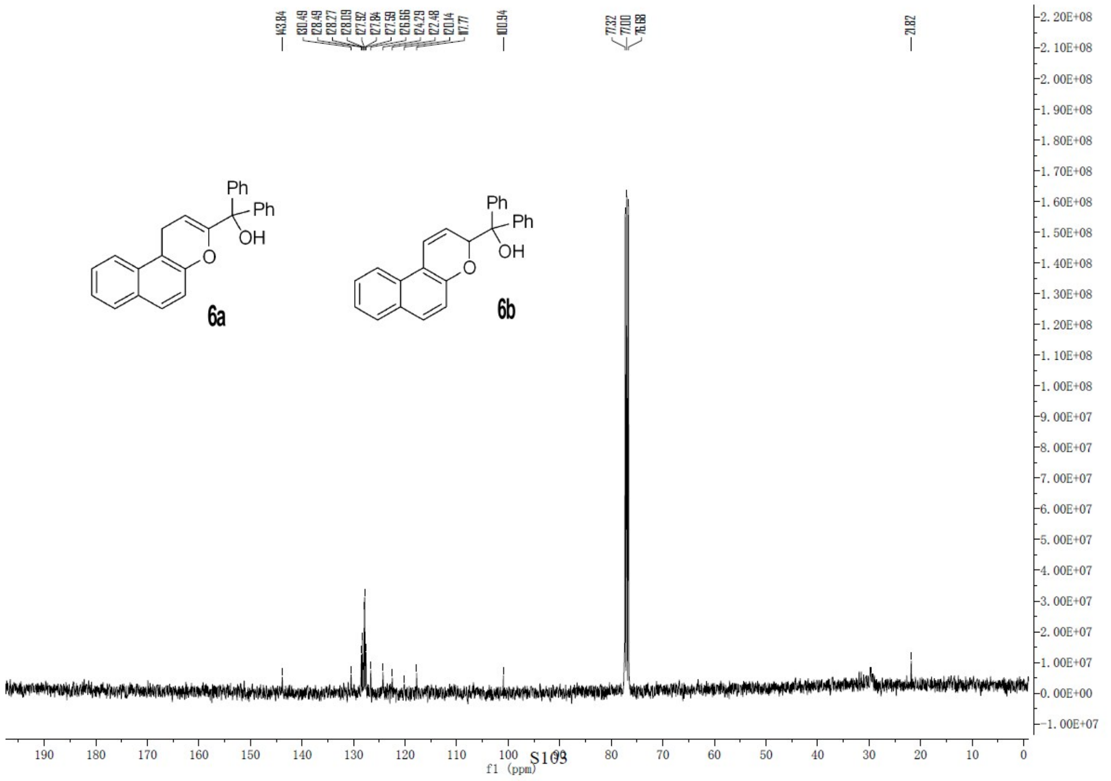

S103

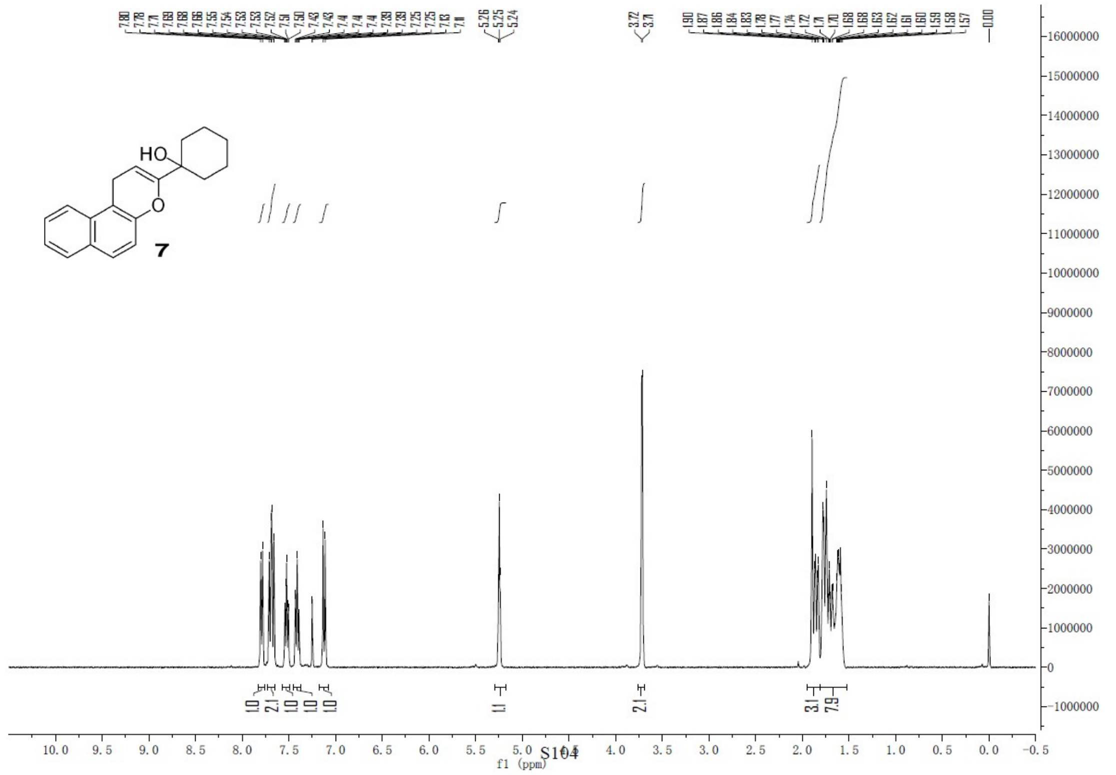
HO
O
7
f1 (ppm)

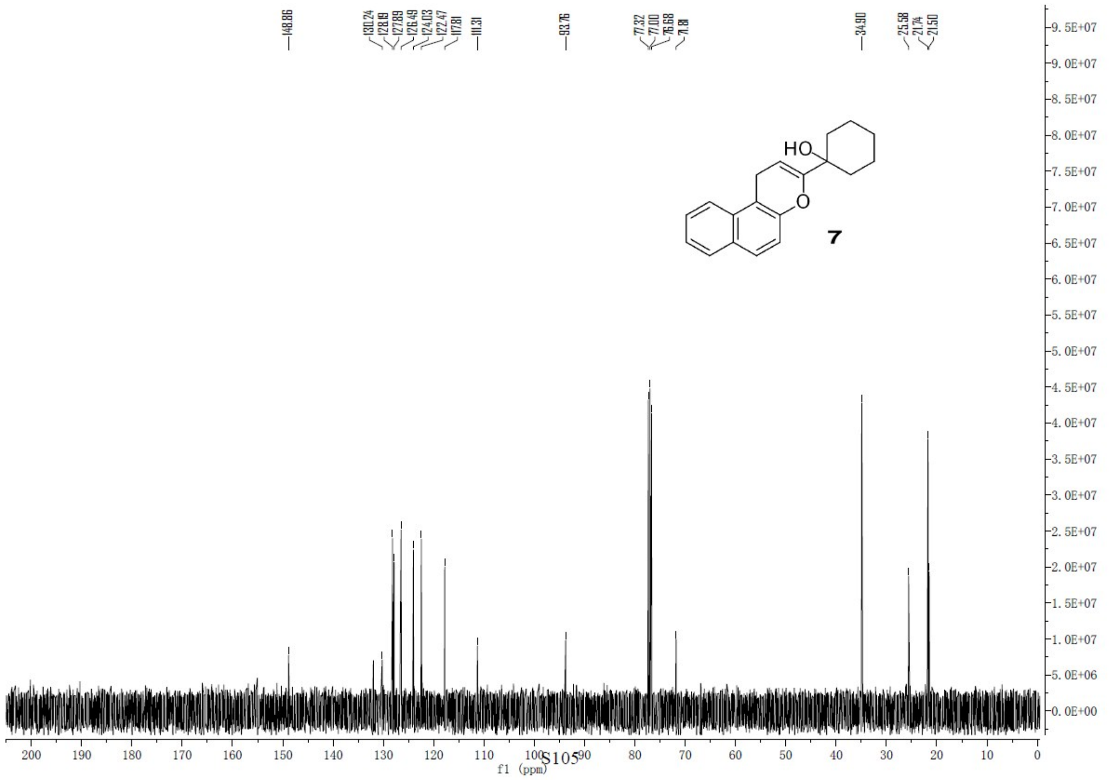
HO
O
7
f1 (ppm)

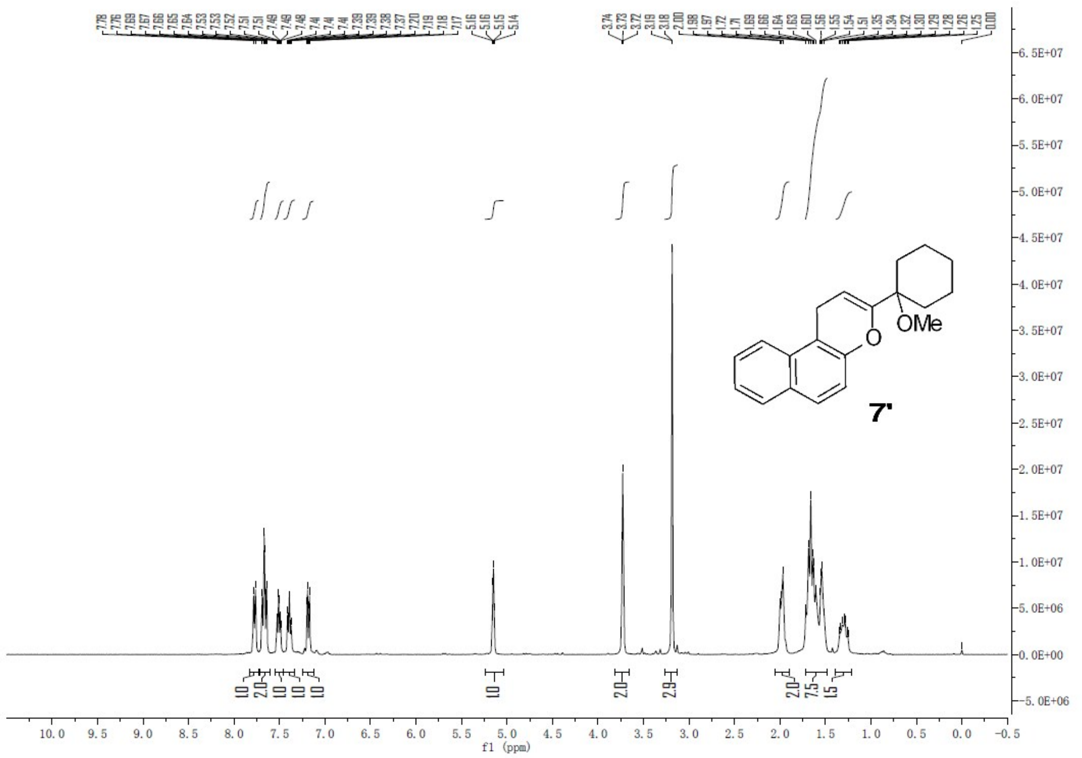

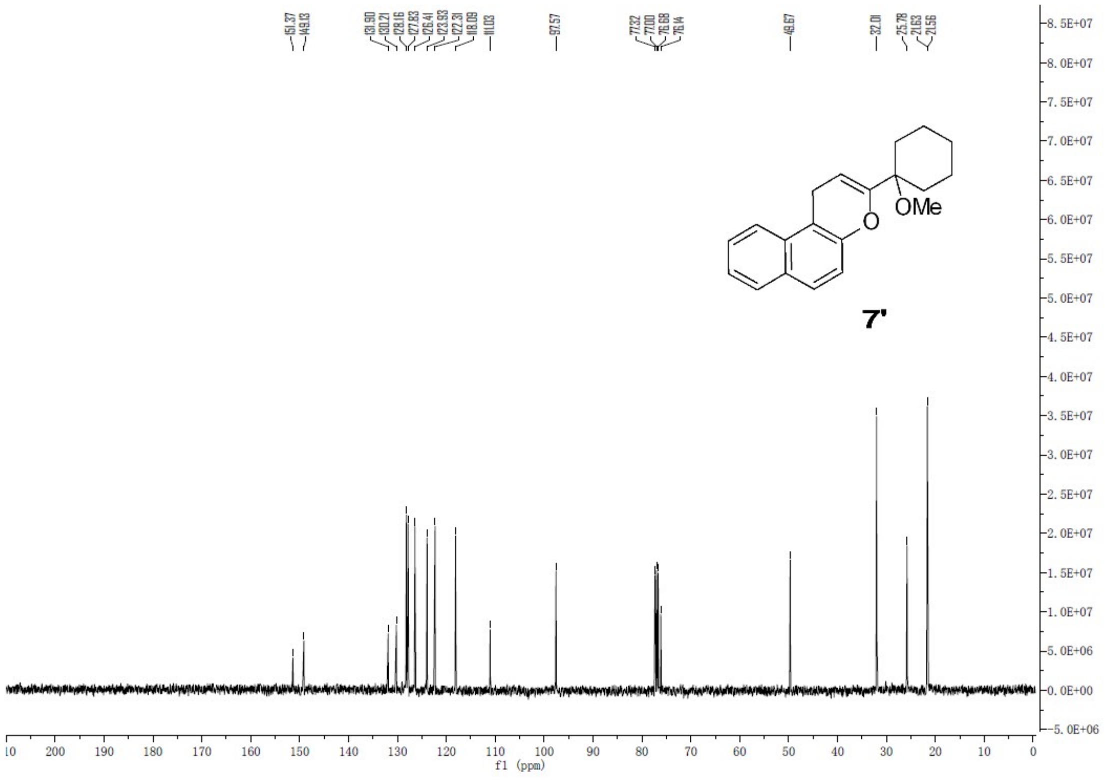

Supplement: Supplementary file 1 [file SC-010-C8SC03837F-s001.pdf]
